# Association of Inflammatory Factors and Calcium Metabolism With Arthritis in Patients With Inflammatory Bowel Disease: Evidence From Mediated Mendelian Randomization

**DOI:** 10.1155/mi/1675577

**Published:** 2025-07-28

**Authors:** Sinan Xiao, Kairong Su, Hongliang Gao, Chenyang Qiao, Sumei Sha, Xin Liu, Haitao Shi

**Affiliations:** ^1^Department of Gastroenterology, The Second Affiliated Hospital of Xi'an Jiaotong University, Xi'an, Shaanxi, China; ^2^The Second Department of Gastroenterology, The First Affiliated Hospital of Xinjiang Medical University, Urumqi, Xinjiang, China

**Keywords:** inflammatory bowel disease, Mendelian randomization, spondyloarthropathies

## Abstract

**Background:** Beyond intestinal inflammation, inflammatory bowel disease (IBD) is associated with many extraintestinal manifestations, particularly arthritis. However, systematic evidence regarding causal relationships between IBD and clinically prevalent arthritis subtypes remains limited.

**Methods:** We conducted bidirectional two-sample Mendelian randomization (MR) analyses to assess causal associations between IBD (Crohn's disease [CD], ulcerative colitis [UC]) and seven arthritis subtypes: rheumatoid arthritis (RA), ankylosing spondylitis (AS), psoriatic arthritis (PsA), osteoarthritis (OA), reactive arthritis (ReA), gout and pyogenic arthritis (PA). A two-step MR (TSMR) analyses was subsequently performed to evaluate potential mediators across four domains: metabolites of gut microbiota, serum biochemical indicators, inflammatory/immune factors, and nutritional/metabolic indicators in IBD-AS/PsA/ReA pathways.

**Results:** MR analysis revealed that IBD increased the risk of AS (OR = 1.21, 95% CI: 1.11–1.32, *P*_IVW_ < 0.001), PsA (OR = 1.18, 95% CI: 1.05–1.33, *P*_IVW_ = 0.007), and ReA (OR = 1.11, 95% CI: 1.04–1.18, *P*_IVW_ = 0.003). Subgroup analyses revealed CD increased the risk of AS (OR = 1.16, 95% CI: 1.07–1.27, *P*_IVW_ < 0.001) and UC increased the risk of ReA (OR = 1.14, 95% CI: 1.04–1.24, *P*_IVW_ = 0.005). The first step of the mediation MR showed that IBD was associated with increased butyrate levels, decreased serotonin levels, increased C-reactive protein (CRP), increased interleukin-6 (IL-6), increased percentage of neutrophils, decreased percentage of lymphocytes, and decreased total body bone mineral density, but the second step of the analysis revealed no significant evidence that the above factors were mediators of the causal effects of IBD on AS, PsA, and ReA.

**Conclusion:** This study establishes the causal effect of IBD on AS, PsA, and ReA. The absence of significant mediation effects suggests that IBD-associated gut dysbiosis, systemic inflammation, and calcium metabolic disturbances may not directly drive arthritis pathogenesis, challenging their utility as predictive biomarkers for arthritis development in IBD patients.

## 1. Introduction

Inflammatory bowel disease (IBD), encompassing ulcerative colitis (UC) and Crohn's disease (CD), is a chronic, nonspecific, recurrent, and inflammatory disease that primarily affects the intestinal tract. In addition to intestinal inflammation, IBD frequently manifests systemically, involving organs such as the joints (peripheral and axial arthritis), skin (erythema nodosum), liver (primary sclerosing cholangitis), and eyes (anterior uveitis) [1, 2]. These systemic features are collectively termed extraintestinal manifestations (EIMs) of IBD. Musculoskeletal involvement dominates EIMs, with joint disease prevalence ranging from 10% to 20% in CD and 4% to 14% in UC [3].

IBD-associated arthritis, classified as enteropathic arthritis under spondyloarthropathies (SpA), represents a distinct entity. However, clinical observational studies also suggest associations between IBD and other arthritis subtypes. Joint involvement necessitates therapeutic adjustments in IBD management. Both traditional nonsteroidal anti-inflammatory drugs (NSAIDs) and selective COX-2 inhibitors (COXIBs) are considered first-line therapeutic agents for SpA. However, their efficacy in reducing recurrent activity in patients with IBD remains a topic of debate. The majority of NSAID labels include recommendations from regulatory agencies against their use in IBD patients [4]. The contradictory applications of these drugs present a significant challenge for gastroenterologists.

Although several observational studies have identified such correlations in clinical practice, it is possible that the conclusions of observational studies are influenced by confounding factors and reverse causation. For ethical reasons, the conduct of clinical trials for these two types of diseases is also precluded. To date, no comprehensive investigation has been conducted into the potential causal relationship between IBD and various forms of arthritis.

To address this knowledge gap, we employed Mendelian randomization (MR). This method uses the random distribution of genetic variation as a natural experiment to explore causal relationships between specific exposure factors and outcomes. Genetic variation is randomly assigned to offspring during gamete formation, making it less susceptible to interference from confounding factors or reverse causation compared to traditional randomized controlled trials. In situations involving the interaction of environmental and genetic factors, the MR method utilizes single nucleotide polymorphisms (SNPs) as instrumental variables (IVs) to circumvent the influence of confounding factors and to make more accurate causal judgments than observational studies [5, 6]. Two-step MR (TSMR), a method of mediated MR, can help us identify potential intermediate variables that may exist between exposure and outcome [7].

## 2. Methods

### 2.1. Data Source and Ethics Approval

Summary statistics for IBD, CD, and UC in European populations were derived from the Genome-Wide Association Study (GWAS) data published by the International IBD Genetics Consortium (IIBDGC) and included in the IEU GWAS database (https://gwas.mrcieu.ac.uk/). The FinnGen consortium [8] (https://www.finngen.fi/en) provided summary statistics for rheumatoid arthritis (RA), ankylosing spondylitis (AS), psoriatic arthritis (PsA), osteoarthritis (OA), reactive arthritis (ReA), gout and pyogenic arthritis (PA) in the European population. Detailed characteristics of the study populations are provided in Table 1 and Table S1.

All original studies received ethical approval from relevant institutional review boards with written informed consent obtained from all participants. This study was exempt from institutional ethics review as it involved secondary analysis of de-identified, publicly available datasets.

### 2.2. Study Design

**Figure 1:**
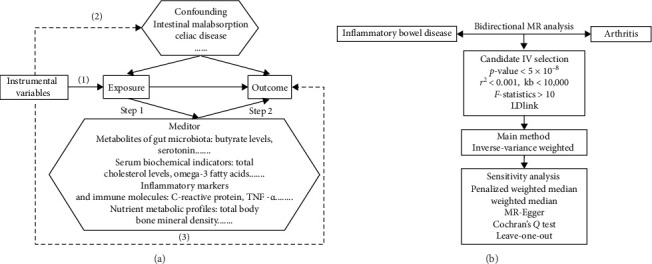
Workflow of two-step mendelian randomization (MR) and three basic assumptions of MR. The path of solid line is significant and dashed paths should not exist (A). Workflow of bidirectional two-sample MR (B).

The research flowchart and key assumptions of MR are shown in Figure 1. The initial objective was to investigate the causal relationships between IBD, CD, and UC with RA, AS, PsA, OA, ReA, gout and PA in European populations through a bidirectional MR. Our study adhered to the three key assumptions of MR: (1) IVs were strongly related to exposure; (2) IVs were independent of confounders affecting the exposure-outcome relationship; (3) IVs influenced the outcome solely through the exposure [9]. When selecting IVs, we followed specific criteria. First, SNPs with genome-wide significance (*p* < 5 × 10^−8^) for IBD/CD/UC; for arthritis phenotypes with limited SNPs (<10), a relaxed threshold (*p* < 5 ×  10^−6^) was applied. Next, Linkage disequilibrium (LD) clumping (*r*^2^  < 0.001, window size = 10,000 kb) to ensure independence. Then, Calculation of *R^2^* and *F*-statistics (*F* = [*R^2^*/*K*]/[ (1−*R^2^*)/(*N*−*K*−1)]), excluding SNPs with *F* <10 to mitigate weak instrument bias [10], where *R^2^* represents the cumulative variance explained by the IVs, *K* denotes the number of IVs, and *N* refers to the sample size. Through the aforementioned procedures, we ensured strong associations between the genetic instruments and the exposure (relevance assumption). Subsequently, we examined whether the selected SNPs were associated with other phenotypes (e.g., intestinal malabsorption and psoriasis) and excluded those correlated with known confounding factors through LDlink website (independence assumption). Notably, in PsA analyses, SNPs linked to psoriasis were retained due to their potential mediating role in the causal pathway between IBD and PsA [11].

We employed multiple MR analytical methods, including the inverse-variance weighted (IVW), weighted median (WM), MR-Egger, and penalized weighted median (PWM) approaches. After confirming that all IVs satisfied the MR assumptions, the main analysis was performed using the IVW method. MR-Egger regression [12] and WM were employed to assess pleiotropy. Specifically, MR-Egger regression evaluated the significance of the intercept term (*p* < 0.05 suggests directional pleiotropy). The WM method provided robust estimates by allowing ≤50% invalid IVs [13]. We employed a stepwise analytical approach to assess horizontal pleiotropy: initially examining the consistency in both effect direction and magnitude IVW and WM estimates; when the effect size difference between IVW and WM exceeded 20%, we further conducted MR-Egger regression to test for directional pleiotropy (significance threshold *p* < 0.05 for the intercept term); final determination of no significant horizontal pleiotropy required simultaneous fulfillment of two criteria: (a) Concordant effect directions between IVW and WM methods and (b) nonsignificant MR-Egger intercept (*p* ≥ 0.05). This protocol is to ensure the IVs primarily influenced the outcome through the exposure (exclusivity assumption).

We additionally performed supplementary sensitivity analyses. Heterogeneity was assessed using Cochran's *Q* statistic (calculated with the IVW method). Our study acknowledged the presence of potential heterogeneity (*Q* test *p* < 0.05), which is generally unavoidable in MR studies due to population stratification and other confounding factors. When heterogeneity was detected, the IVW method with multiplicative random effects was adopted as the primary approach. The leave-one-out method was systematically employed to iteratively exclude individual SNPs and subsequently re-estimate the effect sizes, thereby, enabling the identification of potentially influential outlier SNPs that might disproportionately impact the overall results, and presented the results through visualization methods. All analyses were conducted via the TwoSampleMR package in R software, version 4.3.1. As the exposures and outcomes were dichotomous, effect estimates were converted to odds ratios (ORs) to assess the strength of the causal associations. Complete SNP-level data, including genome-wide significance and *F*-statistics, are provided in Tables S2–S10. The results of MR analyses are presented in Tables S11–S19 and Figures S1–S5.

## 3. Results

### 3.1. The Causal Effect of IBD on Arthritis

**Figure 2:**
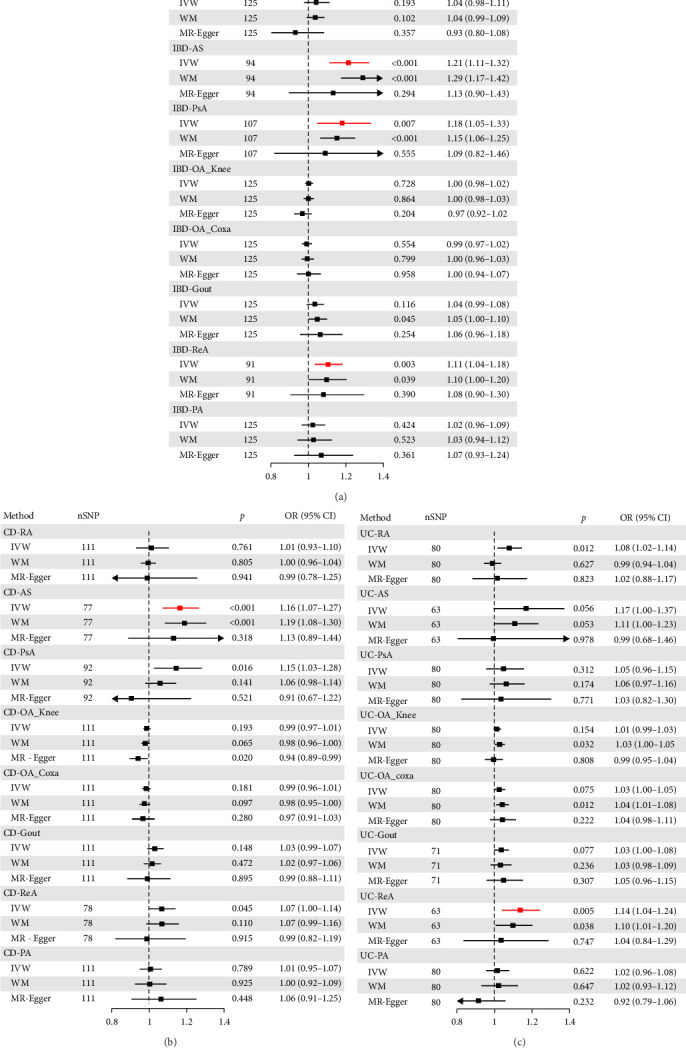
Odds ratios (ORs), 95% confidence intervals and *p* value for the IBD (A), CD (B), and UC (C) to seven arthritis based on the IVW, WM, and MR—Egger methods. AS, ankylosing spondylitis; CD, crohn's disease; Gout, gouty arthritis; IBD, inflammatory bowel disease; IVW, inverse variance weighted; OA_Coxa, osteoarthritis of the hip; OA_Knee, osteoarthritis of the knee; PA, pyogenic arthritis; PsA, psoriatic arthritis; RA, rheumatoid arthritis; ReA, reactive arthritis; UC, ulcerative colitis; WM, weighted median.

The findings of our comprehensive analysis are summarized in the forest plot below.  Figure 2 and Tables S11–S13 demonstrate that IBD is causally associated with AS (IVW: OR = 1.21, 95% CI 1.11–1.32, *p* < 0.001), PsA (IVW: OR = 1.18, 95% CI 1.05–1.33, *p*=0.007), and ReA (IVW: OR = 1.11, 95% CI 1.04–1.18, *p*=0.003). Specifically, CD is causally associated with AS (IVW: OR = 1.16, 95% CI 1.07–1.27, *p* < 0.001). while UC is causally associated with ReA (IVW: OR = 1.14, 95% CI 1.04–1.24, *p*=0.005). Preliminary analyses of CD with PsA, UC with RA, and UC with PsA initially yielded positive associations. However, these results were excluded because the directional consistency of the IVW, WM, and MR-Egger effect sizes was not satisfied.

In sensitivity analyses, MR-Egger intercept tests revealed no evidence of horizontal pleiotropy (*p* > 0.05 for all associations). Although heterogeneity was observed in the positive results (as indicated by Cochran's *Q* test), the use of a multiplicative random-effects model minimized its impact on the conclusions. Additionally, a leave-one-out sensitivity analysis confirmed that no single SNP disproportionately influenced the causal estimates, as the effect direction remained consistent across all iterations. We have presented these results in Figures S1–S5.

### 3.2. The Causal Effect of Arthritis on IBD

Our analyses found no statistically significant causal effects of arthritis on IBD in the European population. While the IVW method suggested a potential association between ReA and IBD (*p* < 0.05), this result was unreliable due to limited SNP availability in the ReA GWAS dataset. After relaxing the threshold (*p* < 1 ×  10^−5^), only 13 SNPs were included, of which merely 3 overlapped with the IBD dataset (a loss rate >70%). Further details are provided in Table S14. Thus, we conclude that no robust evidence supports a causal relationship between ReA and IBD.

### 3.3. TSMR Analysis

In step 1 of the TSMR, we evaluated the causal relationships between IBD, CD, and UC and the following indicators. The findings indicated that with respect to gut microbiota metabolites, IBD was associated with elevated butyrate levels and reduced serotonin levels. With respect to serum biochemical markers, UC correlated with decreased albumin levels. With respect to Inflammatory/immune markers, IBD and CD are associated with increased C-reactive protein (CRP), interleukin-6 (IL-6), neutrophil percentages, and decreased percentages of lymphocyte percentages, UC was associated with increased CRP and IL-6. With respect to nutrient metabolism, IBD and UC showed reduced total body bone mineral density, while IBD and CD were linked to higher urolithiasis rise. Detailed results are presented in Tables S15–S18.

**Figure 3:**
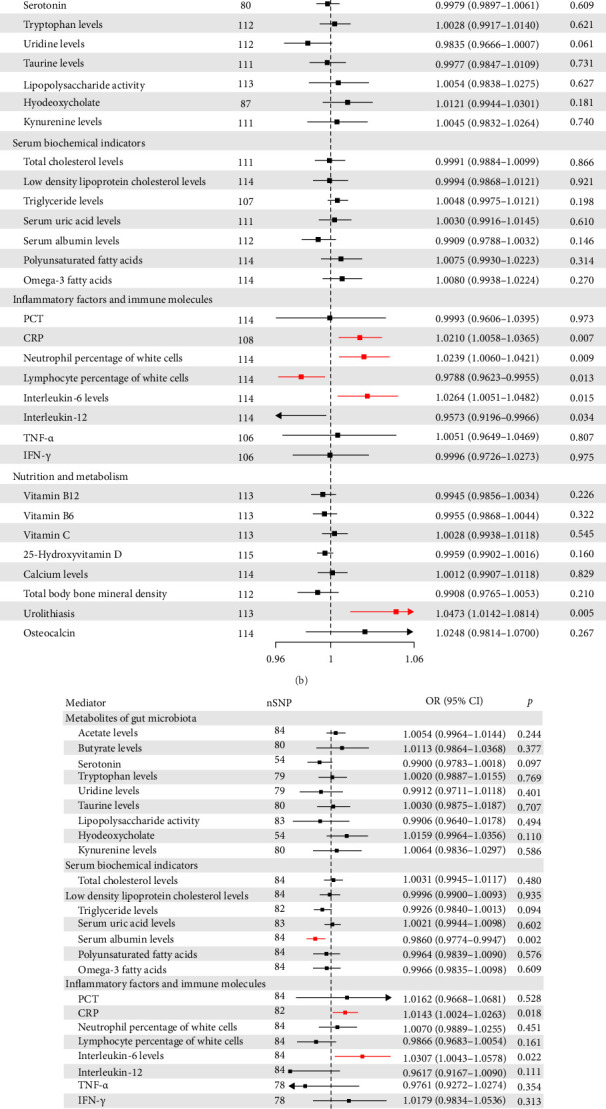
Odds ratios (ORs), 95% confidence intervals, and *p* value for the IBD (A), CD (B), and UC (C) to potential mediators based on the IVW method. Odds ratios (ORs), 95% confidence intervals, and *p* value for the aforementioned nine indicators to the AS, PsA, and ReA (D). AS, ankylosing spondylitis; CD, Crohn's disease; IBD, inflammatory bowel disease; IVW, inverse variance weighted; PsA, psoriatic arthritis; ReA, reactive arthritis; UC, ulcerative colitis.

In step 2 of the TSMR, we investigated the causal relationships between the aforementioned nine indicators and the AS, PsA, and ReA. Elevated neutrophil percentage and reduced lymphocyte percentage were nominally associated with PsA risk; however, conflicting effect directions between IVW and MR-Egger precluded statistical significance. Increased serum albumin and total bone mineral density were linked to PsA and ReA, respectively, with consistent IVW and MR-Egger directions. Nevertheless, this positive outcome could not account for the overall effect, given the contradiction in the mediating effect of the TSMR analysis and the original effect. The IVW analysis forest plot for TSMR is shown in Figure 3, and detailed results are presented in Table S19 in the Supporting Information. Collectively, no biomarkers mediated the causal relationships between IBD and AS, PsA, or ReA.

## 4. Discussion

**Figure 4:**
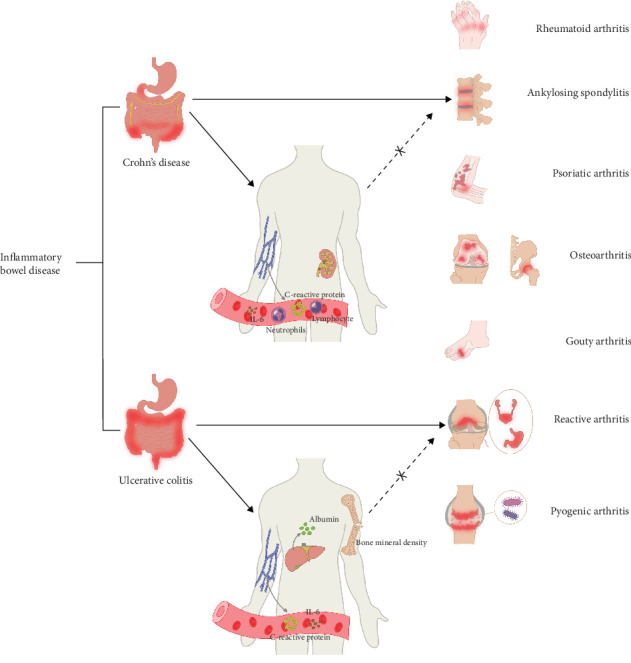
Summary of the results of two-step mediation mendelian randomization analysis. The solid line indicates that the causal relationship has been confirmed, while the dashed line indicates that the causal relationship does not exist.

To our knowledge, this is the first bidirectional two-sample MR study investigating the causal relationship between IBD and clinically observed arthritis risk using large-scale genetic datasets. The core findings of this study are summarized in Figure 4. Our MR analyses identify a positive causal effect of IBD on AS, PsA, and ReA. Although some OR values were close to 1, sensitivity analyses and heterogeneity testing (IVW random effects model) reduced the risk of false positives, and narrow confidence intervals supported the precision of OR estimates. Therefore, we believe that these are causal relationships that require cautious interpretation but cannot be ignored, their value for population-level disease prevention deserves attention. Studies by Yousaf et al. [14] and Zohar et al. [15] reported significant correlations between PsA and both CD and UC. Combined with our findings, these observational associations were likely caused by the positive causal effect of IBD on PsA. Similarly, previous reports indicated that the prevalence of AS in patients with IBD can reach 3.7%–10%, with no significant difference in incidence between UC and CD [16, 17], which aligns with our results. Kassem Sharif's retrospective cohort study demonstrated a markedly elevated overall incidence of IBD in the AS cohort relative to the control group [18]. However, our MR analyses did not support a statistically significant causal effect of AS on IBD. Furthermore, a population-based study conducted in southeastern Norway reported only two UC cases with ReA history (prevalence of 0.7%) [17], consistent with our MR results showing UC (but not CD) as causally linked to ReA. Although the relevance of IBD to ReA has been less frequently discussed in the literature, bioinformatics studies suggest that kynurenine enzymes represent an important host-microbe interaction target for the coevolved ReA of IBD. Consequently, kynurenine-related metabolites were included in our TSMR analyses.

Notably, our study found no causal relationships between IBD and RA, OA, gout, or PA. However, an elevated incidence of RA [19], OA [20], and gout [21] in patients with IBD can frequently be identified in observational studies. Additionally, there have been several reports of patients with IBD presenting with PA [22–24]. In light of the advantages of MR, unidentified confounding factors may have contributed to these observed correlations. Prospective studies are needed to clarify these associations.

The discovery of the association between IBD and arthritis has prompted further investigation into the potential mechanisms involved. Specifically, the gut microbiota is regarded as a crucial mediator [25]. Although previous MR studies showed that gut microbiota does not seem to play a significant mediating role in IBD-associated AS or PsA [26], some researchers still believe that certain metabolites produced by bacteria move from the “leaky gut” into the bloodstream, leading to systemic inflammation, which then reaches the joints, resulting in various types of arthritis [27]. Additionally, intestinal inflammation may trigger immune dysregulation and cytokine release, potentially driving arthritis [28]. Abnormal calcium metabolism in IBD patients [29], possibly linked to malabsorption or chronic inflammation, warrants exploration of an “IBD-calcium-arthritis” pathway. The causal relationships identified in this study, in conjunction with the existing research, guided the selection of 32 indicators from the IEU OpenGWAS database for TSMR analysis. These indicators can be categorized into four domains: gut microbiota metabolites (e.g., succinate), serum biochemical indicators (e.g., albumin), inflammatory factors and immune molecules (e.g., tumor necrosis factor alpha (TNF-α)), and nutrition and metabolism (e.g., blood calcium and vitamin D).

Although several potential mediators (e.g., butyrate, serotonin, CRP, and total body bone mineral density) were identified, no statistically significant mediating effects were observed in the association between IBD and SpA. Thus, these indicators cannot be considered reliable biomarkers for predicting arthritis development in IBD patients. Systematic inflammatory markers and metabolic abnormalities may merely reflect consequences of IBD rather than initiating factors for SpA. This suggests that IBD-associated SpA may develop insidiously, with conventional IBD monitoring (e.g., inflammatory activity, nutritional status, and calcium metabolism) being inadequate for early SpA detection. Even in remission, clinicians should remain vigilant. Previous studies have demonstrated that cytokine concentrations in the synovial fluid of AS patients are higher than serum levels [30], suggesting that joint-specific indicators may offer more sensitive early diagnostic clues than systemic biomarkers. The robust IBD-SpA association underscores the need to optimize IBD therapy by targeting shared immune pathways (e.g., TNF-α [31], interleukin-23 (IL-23)/interleukin-17 (IL-17) [32]) to mitigate joint risks.

Despite the absence of mediation findings, our analyses revealed the potential association between IBD and a range of indicators in the context of step 1 of the TSMR. For example, UC correlated with reduced bone density, while CD was linked to higher urolithiasis risk, highlighting abnormal calcium metabolism in IBD. Furthermore, previous studies have concluded that no significant correlation exists between butyrate and any of the IBD attributes tested in serum or feces (either the presence or absence of IBD or a high or low level of IBD activity) [33]. In contrast, our study demonstrated a positive correlation between IBD and butyrate levels in cerebrospinal fluid, which may provide data support for the mechanism chain of the gut-brain axis.

Notably, our null mediation findings for TNF-α contrast with clinical trials demonstrating TNF-α inhibitors' efficacy in simultaneously improving gut and joint inflammation [34]. To thoroughly discuss the possibility of false-negative results, we acknowledge that the absence of mediating effects could be attributed to methodological constraints and biological complexity. Small mediating effects often require larger sample sizes for detection [7], and our analysis of butyrate, serotonin, and IL-6 might have been underpowered due to the restricted sample sizes of the exposure and mediator datasets. Moreover, neutrophil percentage, CRP, and serum albumin reflect systemic inflammatory status and nutritional conditions, while TNF-α and IL-6 had been measured in serum samples. These factors may collectively limit the ability of IVs to precisely capture pathological changes in either the intestinal mucosa or synovium. Finally, both IBD and SpA are chronic relapsing-remitting diseases with dynamic fluctuations between active and remission phases. Since MR measures the lifelong cumulative effect of genetic predisposition [35], the mediating effects during active phases might have been diluted without stratification by disease activity.

Our findings highlight the need for deeper mechanistic exploration. Due to database constraints, other potential mediators might have been overlooked, such as IL-23R gene polymorphisms [36], fecal calprotectin (a gut-specific inflammatory marker that is also elevated in AS [37]), and matrix metalloproteinase-3 [38] (a biomarker of synovial inflammation). Single-cell sequencing of intestinal and joint biopsy tissues, combined with comparative analysis of cytokines present in serum and synovial fluid during presymptomatic and definitive disease stages, will fill a gap in current research. Further validation through animal models and well-characterized clinical cohorts remains essential.

Limitations of this study must be acknowledged. First, reliance on European GWAS data limits generalizability to other populations, as genetic background, environmental factors, and lifestyle of different populations may lead to significant differences in disease susceptibility. Extrapolation of our findings to Asian, African, or American populations may lead to erroneous conclusions. Second, the sample selection criteria and reference ranges for some indicators were unavailable from existing original studies. Although we provided GWAS IDs for reproducibility, this remains a methodological constraint. In summary, our conclusion—that SpA in IBD is unrelated to gut dysbiosis, disease activity, or calcium metabolism—applies only to the 32 factors analyzed. Future studies with expanded datasets should revisit these pathways.

## 5. Conclusions

Our study confirmed the causal effect of IBD on AS, PsA, and ReA, which provides new evidence for gastroenterologists to monitor joint symptoms during long-term IBD follow-up and highlights the need for multidisciplinary collaboration with rheumatologists.

Through mediation analyses, we concluded that SpA development in IBD patients is likely independent of gut dysbiosis, disease activity, and calcium metabolism. None of the 32 biomarkers analyzed predicted the likelihood of AS, PsA, or ReA in IBD patients. Future studies must identify clinical biomarkers to predict SpA risk and elucidate their mechanisms, which could inform the development of targeted therapies.

## Figures and Tables

**Table 1:** Detailed information related to the study population.

Trait	Population	Sex	Ncase	Ncontral
Exposure				
Inflammatory bowel disease	European	Males and females	31,665	33,977
Crohn's disease	European	Males and females	17,897	33,977
Ulcerative colitis	European	Males and females	13,768	33,977
Outcome				
Rheumatoid arthritis	European	Males and females	13,621	262,844
Ankylosing spondylitis	European	Males and females	3162	294,770
Psoriatic arthropathies	European	Males and females	3537	262,844
Gonarthrosis	European	Males and females	48,836	262,844
Coxarthrosis	European	Males and females	24,255	262,844
Reactive arthropathies	European	Males and females	3058	262,844
Gout	European	Males and females	9568	262,844
Pyogenic arthritis	European	Males and females	2207	262,844

## Data Availability

The primary data used to support the results of this study were obtained from the Genome-Wide Association Study (GWAS) initiated by the International IBD Genetics Consortium (IIBDGC) (https://gwas.mrcieu.ac.uk/) and The FinnGen consortium9 (https://www.finngen.fi/en). Secondary data processing of the primary data is included in the links to the electronic Supporting Information.

## Nomenclature

MR: Mendelian randomization
IVW: Inverse variance weighted
WM: Weighted median
IBD: Inflammatory bowel disease
UC: Ulcerative colitis
CD: Crohn's disease
SpA: Spondyloarthropathies
RA: Rheumatoid arthritis
AS: Ankylosing spondylitis
PsA: Psoriatic arthritis
OA_Knee: Osteoarthritis of the knee
OA_Coxa: Osteoarthritis of the hip
ReA: Reactive arthritis
PA: Pyogenic arthritis
SNPs: Single-nucleotide polymorphisms
IVs: Instrumental variables
TSMR: Two-step Mendelian randomization
GWAS: Genome-Wide Association Study
ORs: Odds ratios
CRP: C-reactive protein
IL-6: Interleukin-6
TNF-α: Tumor necrosis factor alpha
IL-23: Interleukin-23
IL-17: Interleukin-17.

## Supporting Information

Supporting Information Table S1: Details of the study population. Table S2: SNPs for IBD to RA, AS, and PSA identified from GWAS analysis. Table S3: SNPs for IBD to OA and gout identified from GWAS analysis. Table S4: SNPs for IBD to ReA and PA identified from GWAS analysis. Table S5: SNPs for CD to RA, AS, and PSA identified from GWAS analysis. Table S6: SNPs for CD to OA and gout identified from GWAS analysis. Table S7: SNPs for CD to ReA and PA identified from GWAS analysis. Table S8: SNPs for UC to RA, AS, and PSA identified from GWAS analysis. Table S9: SNPs for UC to OA and gout identified from GWAS analysis. Table S10: SNPs for UC to ReA and PA identified from GWAS analysis. Table S11: MR analysis for IBD to arthritis. Table S12: MR analysis for CD to arthritis. Table S13: MR analysis for UC to arthritis. Table S14: MR analysis for arthritis to IBD, CD, and UC. Table S15: MR analysis for IBD, CD, and UC to metabolites of gut microbiota. Table S16: MR analysis for IBD, CD, and UC to serum biochemical indicators. Table S17: MR analysis for IBD, CD, and UC to inflammatory factors and immune molecules. Table S18: MR analysis for IBD, CD, and UC to nutrition and metabolism. Table S19: MR analysis for mediators to arthritis. Figure S1: Figures for IBD to AS. Figure S2: Figures for IBD to PSA. Figure S3: Figures for IBD to ReA. Figure S4: Figures for CD to AS. Figure S5: Figures for UC to ReA.

## References

[1] Garber A., Regueiro M. (2019). Extraintestinal Manifestations of Inflammatory Bowel Disease: Epidemiology, Etiopathogenesis, and Management. *Current Gastroenterology Reports*.

[2] Vavricka S. R., Schoepfer A., Scharl M., Lakatos P. L., Navarini A., Rogler G. (2015). Extraintestinal Manifestations of Inflammatory Bowel Disease. *Inflammatory Bowel Diseases*.

[3] Rogler G., Singh A., Kavanaugh A., Rubin D. T. (2021). Extraintestinal Manifestations of Inflammatory Bowel Disease: Current Concepts, Treatment, and Implications for Disease Management. *Gastroenterology*.

[4] González-Lama Y., Sanz J., Bastida G. (2020). Recomendaciones del Grupo Español de Trabajo en Enfermedad de Crohn y Colitis Ulcerosa (GETECCU) Sobre el Tratamiento de Pacientes con Enfermedad Inflamatoria Intestinal Asociada a Espondiloartritis. *Gastroenterología y Hepatología*.

[5] Davies N. M., Holmes M. V., Davey Smith G. (2018). Reading Mendelian Randomisation Studies: A Guide, Glossary, and Checklist for Clinicians. *BMJ*.

[6] Sekula P., Del Greco M F., Pattaro C., Köttgen A. (2016). Mendelian Randomization as an Approach to Assess Causality Using Observational Data. *Journal of The American Society of Nephrology*.

[7] Carter A. R., Sanderson E., Hammerton G. (2021). Mendelian Randomisation for Mediation Analysis: Current Methods and Challenges for Implementation. *European Journal of Epidemiology*.

[8] Kurki M. I., Karjalainen J., Palta P. (2023). Author Correction: FinnGen Provides Genetic Insights From a Well-Phenotyped Isolated Population. *Nature*.

[9] Skrivankova V. W., Richmond R. C., Woolf B. A. R. (2021). Strengthening the Reporting of Observational Studies in Epidemiology Using Mendelian Randomization: The STROBE-MR Statement. *JAMA*.

[10] Burgess S., Davies N. M., Thompson S. G. (2016). Bias Due to Participant Overlap in Two-Sample Mendelian Randomization. *Genetic Epidemiology*.

[11] Ono K., Kishimoto M., Deshpande G. A. (2022). Clinical Characteristics of Patients With Spondyloarthritis and Inflammatory Bowel Disease Versus Inflammatory Bowel Disease-Related Arthritis. *Rheumatology International*.

[12] Bowden J., Davey Smith G., Burgess S. (2015). Mendelian Randomization With Invalid Instruments: Effect Estimation and Bias Detection Through Egger Regression. *International Journal of Epidemiology*.

[13] Bowden J., Davey Smith G., Haycock P. C., Burgess S. (2016). Consistent Estimation in Mendelian Randomization With Some Invalid Instruments Using a Weighted Median Estimator. *Genetic Epidemiology*.

[14] Yousaf A., Raiker R., Davis S. M., Gayam S., Zinn Z. (2021). Association Between Psoriasis, Psoriatic Arthritis and Gastrointestinal Disease: An Exploratory Nationwide Inpatient Sample Analysis. *Wiener klinische Wochenschrift*.

[15] Zohar A., Cohen A. D., Bitterman H. (2016). Gastrointestinal Comorbidities in Patients With Psoriatic Arthritis. *Clinical Rheumatology*.

[16] De Vlam K., Mielants H., Cuvelier C., De Keyser F., Veys E. M., De Vos M. (2000). Spondyloarthropathy is Underestimated in Inflammatory Bowel Disease: Prevalence and HLA Association. *The Journal of Rheumatology*.

[17] Palm O., Moum B., Ongre A., Gran J. T. (2002). Prevalence of Ankylosing Spondylitis and Other Spondyloarthropathies Among Patients With Inflammatory Bowel Disease: A Population Study (the IBSEN Study). *Journal of Rheumatology*.

[18] Sharif K., Lahat A., Patt Y. S. (2024). Exploring the Link Between Ankylosing Spondylitis and Inflammatory Bowel Disease: A Retrospective Cohort Study. *Israel Medical Association Journal*.

[19] Cohen R., Robinson D., Paramore C., Fraeman K., Renahan K., Bala M. (2008). Autoimmune Disease Concomitance Among Inflammatory Bowel Disease Patients in the United States, 2001–2002. *Inflammatory Bowel Diseases*.

[20] Fernandes B. M., Rosa-Gonçalves D., Magro F., Costa L., Bernardes M. (2022). Musculoskeletal Manifestations in a Portuguese Cohort of 235 Inflammatory Bowel Disease Patients. *ARP Rheumatol*.

[21] Hamid O., Alsabbagh Alchirazi K., Eltelbany A., Nanah R., Regueiro M. (2023). Increased Prevalence of Gout in Patients With Inflammatory Bowel Disease: A Population-Based Study. *JGH Open*.

[22] Barnes M., Bush C., Jones J. (2019). Pyogenic Sacroiliitis: A Rare Complication of Inflammatory Bowel Disease. *The American Journal of Emergency Medicine*.

[23] Özpolat H. T., Baran B., Akyüz F. (2022). Septic Arthritis: A Presentation of Crohn’s Disease. *The Lancet Gastroenterology & Hepatology*.

[24] Zaidi R., Dala-Ali B., Dalton D. J. N. (2011). Septic Arthritis as a Presentation of Crohn’s Disease. *BMJ Military Health*.

[25] Gracey E., Vereecke L., McGovern D. (2020). Revisiting the Gut—Joint Axis: Links Between Gut Inflammation and Spondyloarthritis. *Nature Reviews Rheumatology*.

[26] Lu W., Cen J., Dai Q., Tao H., Peng L. (2024). Gut Microbiota Does Not Play a Mediating Role in the Causal Association Between Inflammatory Bowel Disease and Several Its Associated Extraintestinal Manifestations: A Mendelian Randomization Study. *Frontiers in Immunology*.

[27] Longo U. G., Lalli A., Bandini B. (2024). Role of the Gut Microbiota in Osteoarthritis, Rheumatoid Arthritis, and Spondylarthritis: An Update on the Gut—Joint Axis. *International Journal of Molecular Sciences*.

[28] Felice C., Dal Buono A., Gabbiadini R., Rattazzi M., Armuzzi A. (2023). Cytokines in Spondyloarthritis and Inflammatory Bowel Diseases: From Pathogenesis to Therapeutic Implications. *International Journal of Molecular Sciences*.

[29] Jahnsen J., Falch J. A., Mowinckel P., Aadland E. (2009). Vitamin D Status, Parathyroid Hormone and Bone Mineral Density in Patients With Inflammatory Bowel Disease. *Scandinavian Journal of Gastroenterology*.

[30] Ugur M., Baygutalp N. K., Melikoglu M. A., Baygutalp F., Altas E. U., Seferoglu B. (2015). Elevated Serum Interleukin-23 Levels in Ankylosing Spondylitis Patients and the Relationship With Disease Activity. *Nagoya Journal of Medical Science*.

[31] Mihai I. R., Burlui A. M., Rezus M. (2023). Inflammatory Bowel Disease as a Paradoxical Reaction to Anti-TNF-*α* Treatment—A Review. *Life*.

[32] Wendling D., Prati C., Chouk M., Verhoeven F. (2020). Effects of Anti-IL-23 and Anti-IL-17: The Hidden Side of Spondyloarthritis Polymorphism?. *Joint Bone Spine*.

[33] Di’Narzo A. F., Houten S. M., Kosoy R. (2022). Integrative Analysis of the Inflammatory Bowel Disease Serum Metabolome Improves Our Understanding of Genetic Etiology and Points to Novel Putative Therapeutic Targets. *Gastroenterology*.

[34] Jang D. I., Lee A. H., Shin H. Y. (2021). The Role of Tumor Necrosis Factor Alpha (TNF-*α*) in Autoimmune Disease and Current TNF-*α* Inhibitors in Therapeutics. *International Journal of Molecular Sciences*.

[35] Gill D., Walker V. M., Martin R. M., Davies N. M., Tzoulaki I. (2020). Comparison With Randomized Controlled Trials as a Strategy for Evaluating Instruments in Mendelian Randomization. *International Journal of Epidemiology*.

[36] Küçükşahin O., Ateş A., Türkçapar N. (2020). Association Between Single Nucleotide Polymorphisms in Prospective Genes and Susceptibility to Ankylosing Spondylitis and Inflammatory Bowel Disease in a Single Centre in Turkey. *The Turkish Journal of Gastroenterology*.

[37] Klingberg E., Strid H., Ståhl A. (2017). A Longitudinal Study of Fecal Calprotectin and the Development of Inflammatory Bowel Disease in Ankylosing Spondylitis. *Arthritis Research & Therapy*.

[38] Ondrejčáková L., Gregová M., Bubová K., Šenolt L., Pavelka K. (2024). Serum Biomarkers and Their Relationship to Axial Spondyloarthritis Associated With Inflammatory Bowel Diseases. *Autoimmunity Reviews*.

