# Supplementary material for: Association of Inflammatory Factors and Calcium Metabolism With Arthritis in Patients With Inflammatory Bowel Disease: Evidence From Mediated Mendelian Randomization
Source: Mediators Inflamm. 2025 Jul 28;2025:1675577. doi: 10.1155/mi/1675577 (PMC12321438; doi:10.1155/mi/1675577)
Supplement: Supporting Information — Table S1: Details of the study population. Table S2: SNPs for IBD to RA, AS, and PSA identified from GWAS analysis. Table S3: SNPs for IBD to OA and gout identified from GWAS analysis. Table S4: SNPs for IBD to ReA and PA identified from GWAS analysis. Table S5: SNPs for CD to RA, AS, and PSA identified from GWAS analysis. Table S6: SNPs for CD to OA and gout identified from GWAS analysis. Table S7: SNPs for CD to ReA and PA identified from GWAS analysis. Table S8: SNPs for UC to RA, AS, and PSA identified from GWAS analysis. Table S9: SNPs for UC to OA and gout identified from GWAS analysis. Table S10: SNPs for UC to ReA and PA identified from GWAS analysis. Table S11: MR analysis for IBD to arthritis. Table S12: MR analysis for CD to arthritis. Table S13: MR analysis for UC to arthritis. Table S14: MR analysis for arthritis to IBD, CD, and UC. Table S15: MR analysis for IBD, CD, and UC to metabolites of gut microbiota. Table S16: MR analysis for IBD, CD, and UC to serum biochemical indicators. Table S17: MR analysis for IBD, CD, and UC to inflammatory factors and immune molecules. Table S18: MR analysis for IBD, CD, and UC to nutrition and metabolism. Table S19: MR analysis for mediators to arthritis. Figure S1: Figures for IBD to AS. Figure S2: Figures for IBD to PSA. Figure S3: Figures for IBD to ReA. Figure S4: Figures for CD to AS. Figure S5: Figures for UC to ReA. [file 1675577.f1.pdf]

Supplementary Table 1. Details of the study population

	GWAS ID/PMID	Trait	Year	Population	Sample Size	Number of SNPs	Sex	ncase	ncontrol	Author
Exposure	ieu-a-294/ 26192919	Inflammatory bowel disease	2015	European	65,642	157,116	Males and Females	31,665	33,977	Liu
	ieu-a-12/ 26192919	Crohn's disease	2015	European	51,874	124,888	Males and Females	17,897	33,977	Liu
	ieu-a-970/ 26192919	Ulcerative colitis	2015	European	47,745	156,116	Males and Females	13,768	33,977	Liu
Mediator	ebi-a-GCST90092803/35213538	Acetate levels	2022	European	115,050	11,590,399	NA	NA	NA	Richardson TG
	ebi-a-GCST90026310/ 33437055	Butyrate (4:0) levels	2021	European	291	6,865,381	Males and Females	NA	NA	Panyard DJ
	met-a-358/ 24816252	Serotonin (5HT)	2014	European	6,139	2,545,835	Males and Females	NA	NA	Shin
	ebi-a-GCST90026280/ 33437055	Tryptophan levels	2021	European	291	6,853,216	Males and Females	NA	NA	Panyard DJ
	ebi-a-GCST90026036/ 33437055	Uridine levels	2021	European	291	6,863,016	Males and Females	NA	NA	Panyard DJ
	ebi-a-GCST90026035/ 33437055	Taurine levels	2021	European	291	6,856,779	Males and Females	NA	NA	Panyard DJ
	ebi-a-GCST90032674/ 34668383	Serum lipopolysaccharide activity	2021	European	11,296	9,683,696	Males and Females	NA	NA	Jaakko L
	ebi-a-GCST90026011/ 33437055	Kynurenine levels	2021	European	291	6,864,325	Males and Females	NA	NA	Panyard DJ
	ebi-a-GCST90018974/ 34594039	Total cholesterol levels	2021	European	344,278	19,043,498	Males and Females	NA	NA	Sakaue S
	ebi-a-GCST90002412/32493714	Low density lipoprotein cholesterol levels	2020	European	431,167	16,293,344	Males and Females	NA	NA	Klimentidis YC
	ebi-a-GCST90014014/ 34017140	Triglyceride levels	2021	European	389,562	10,783,708	Males and Females	NA	NA	Mbatchou J
	ebi-a-GCST90018977/ 34594039	Serum uric acid levels	2021	European	343,836	19,041,286	Males and Females	NA	NA	Sakaue S
	ebi-a-GCST90018945/ 34594039	Serum albumin levels	2021	European	315,268	19,053,186	Males and Females	NA	NA	Sakaue S
	met-d-PUFA	Polyunsaturated fatty acids	2020	European	114,999	12,321,875	Males and Females	NA	NA	Borges CM
	met-d-Omega_3	Omega-3 fatty acids	2020	European	114,999	12,321,875	Males and Females	NA	NA	Borges CM
	prot-a-341/ 29875488	Procalcitonin	2018	European	3,301	10,534,735	Males and Females	NA	NA	Sun BB
	ebi-a-GCST90014002/ 34017140	C reactive protein levels	2021	European	389,057	10,783,679	Males and Females	NA	NA	Mbatchou J
	ebi-a-GCST90002399/ 32888494	Neutrophil percentage of white cells	2020	European	408,112	40,312,502	Males and Females	NA	NA	Vuckovic D
	ebi-a-GCST90002389/ 32888494	Lymphocyte percentage of white cells	2020	European	408,112	40,312,257	Males and Females	NA	NA	Vuckovic D
	ebi-a-GCST90012005/ 33067605	Interleukin-6 levels	2020	European	21,758	11,782,139	Males and Females	NA	NA	Folkersen L

	prot-a-1468/ 29875488	Interleukin-12	2018	European	3,301	10,534,735	Males and Females	NA	NA	Sun BB
	ebi-a-GCST004426 / 27989323	Tumor necrosis factor alpha levels	2016	European	3,454	9,500,449	Males and Females	NA	NA	Ahola-Olli AV
	ebi-a-GCST004456 / 27989323	Interferon gamma levels	2016	European	7,701	9,785,363	Males and Females	NA	NA	Ahola-Olli AV
	ukb-b-19524	Vitamin B12	2018	European	64,979	9,851,867	Males and Females	NA	NA	Ben Elsworth
	ukb-b-7864	Vitamin B6	2018	European	64,979	9,851,867	Males and Females	NA	NA	Ben Elsworth
	ukb-b-19390	Vitamin C	2018	European	64,979	9,851,867	Males and Females	NA	NA	Ben Elsworth
	ebi-a-GCST010144 / 32059762	Serum 25-Hydroxyvitamin D levels	2020	European	443,734	15,847,859	Males and Females	NA	NA	Manousaki D
	ebi-a-GCST90018951/ 34594039	Calcium levels	2021	European	315,153	19,052,100	Males and Females	NA	NA	Sakaue S
	ebi-a-GCST005348 / 29304378	Total body bone mineral density	2018	European	56,284	16,162,733	Males and Females	NA	NA	Medina-Gomez C
	ebi-a-GCST90018935/ 34594039	Urolithiasis	2021	European	488,346	24,183,273	Males and Females	NA	NA	Sakaue S
	prot-a-246/ 29875488	Osteocalcin	2018	European	3,301	10,534,735	Males and Females	NA	NA	Sun BB
	**Phenotype**	**Release**	**Get Link**					**ncase**	**ncontrol**	**number of genome-wide significant hits**
**Outcome**	Rheumatoid arthritis	R10	https://storage.googleapis.com/finngen-public-data-r10/summary_stats/finngen_R10_M13_RHEUMA.gz					13621	262844	31
	Ankylosing spondylitis	R10	https://storage.googleapis.com/finngen-public-data-r10/summary_stats/finngen_R10_M13_ANKYLOSPON.gz					3162	294770	22
	Psoriatic arthropathies	R10	https://storage.googleapis.com/finngen-public-data-r10/summary_stats/finngen_R10_M13_PSORIARTH.gz					3537	262844	16
	Gonarthrosis	R10	https://storage.googleapis.com/finngen-public-data-r10/summary_stats/finngen_R10_M13_ARTHROSIS_KNEE.gz					48836	262844	86
	Coxarthrosis	R10	https://storage.googleapis.com/finngen-public-data-r10/summary_stats/finngen_R10_M13_ARTHTROSIS_COX.gz					24255	262844	59
	Reactive arthropathies	R10	https://storage.googleapis.com/finngen-public-data-r10/summary_stats/finngen_R10_M13_REACTARTH.gz					3058	262844	7
	Gout	R10	https://storage.googleapis.com/finngen-public-data-r10/summary_stats/finngen_R10_M13_GOUT.gz					9568	262844	26
	Pyogenic arthritis	R10	https://storage.googleapis.com/finngen-public-data-r10/summary_stats/finngen_R10_M13_PYOGARTH.gz					2207	262844	3

Supplementary Table 2. SNPs for IBD to RA, AS and PSA identified from GWAS analysis

IBD to RA			IBD to AS			IBD to PSA		
SNPs	*F*-statistics	genome-wide significance	SNPs	*F*-statistics	genome-wide significance	SNPs	*F*-statistics	genome-wide significance
rs10142466	193.8873	1.08E-08	rs10758669	1169.886	4.70E-48	rs10758669	1169.886	4.70E-48
rs10758669	1169.886	4.70E-48	rs10761659	1368.642	4.97E-53	rs10761659	1368.642	4.97E-53
rs10761659	1368.642	4.97E-53	rs10800309	911.3654	6.15E-37	rs10800309	911.3654	6.15E-37
rs10800309	911.3654	6.15E-37	rs10878302	193.3339	5.26E-09	rs10878302	193.3339	5.26E-09
rs10878302	193.3339	5.26E-09	rs10956252	379.5455	2.26E-16	rs10956252	379.5455	2.26E-16
rs10956252	379.5455	2.26E-16	rs11152949	554.949	7.25E-23	rs11152949	554.949	7.25E-23
rs11152949	554.949	7.25E-23	rs11230563	345.2953	1.71E-14	rs11230563	345.2953	1.71E-14
rs11230563	345.2953	1.71E-14	rs11236797	1308.07	9.32E-52	rs11236797	1308.07	9.32E-52
rs11236797	1308.07	9.32E-52	rs11641016	453.2093	9.51E-17	rs11641016	453.2093	9.51E-17
rs11641016	453.2093	9.51E-17	rs11677953	345.3509	2.92E-15	rs11677953	345.3509	2.92E-15
rs11677953	345.3509	2.92E-15	rs11691685	255.3177	7.27E-11	rs11691685	255.3177	7.27E-11
rs11691685	255.3177	7.27E-11	rs11713774	250.8475	3.92E-11	rs11713774	250.8475	3.92E-11
rs11713774	250.8475	3.92E-11	rs11793497	1387.172	1.71E-54	rs11793497	1387.172	1.71E-54
rs11793497	1387.172	1.71E-54	rs1182188	209.8552	1.08E-09	rs1182188	209.8552	1.08E-09
rs1182188	209.8552	1.08E-09	rs12318183	657.7615	1.67E-27	rs12318183	657.7615	1.67E-27
rs12318183	657.7615	1.67E-27	rs12585310	247.8039	5.25E-11	rs12585310	247.8039	5.25E-11
rs12411259	188.2763	6.18E-09	rs1267499	239.5031	5.22E-11	rs1267499	239.5031	5.22E-11
rs1250566	508.7193	4.77E-20	rs12718244	323.4647	3.35E-14	rs12718244	323.4647	3.35E-14
rs12585310	247.8039	5.25E-11	rs12722515	307.5065	4.57E-12	rs12722515	307.5065	4.57E-12
rs1267499	239.5031	5.22E-11	rs12796489	3051.119	2.87E-69	rs12796489	3051.119	2.87E-69
rs12718244	323.4647	3.35E-14	rs1292053	279.6435	9.89E-13	rs1292053	279.6435	9.89E-13
rs12722515	307.5065	4.57E-12	rs1297258	742.3875	5.38E-30	rs1297258	742.3875	5.38E-30
rs12796489	3051.119	2.87E-69	rs13107612	258.4067	1.62E-11	rs13107612	258.4067	1.62E-11
rs1292053	279.6435	9.89E-13	rs13204742	214.166	5.39E-10	rs13204742	214.166	5.39E-10
rs1297258	742.3875	5.38E-30	rs13407913	476.5494	1.69E-20	rs13407913	476.5494	1.69E-20
rs13107612	258.4067	1.62E-11	rs1363907	373.9241	4.87E-15	rs1363907	373.9241	4.87E-15
rs13204742	214.166	5.39E-10	rs1388585	404.0949	6.85E-22	rs1388585	404.0949	6.85E-22
rs13407913	476.5494	1.69E-20	rs1569328	213.5132	3.21E-09	rs1569328	213.5132	3.21E-09
rs1363907	373.9241	4.87E-15	rs17293632	479.1136	2.71E-20	rs17293632	479.1136	2.71E-20
rs1388585	404.0949	6.85E-22	rs17651741	175.761	2.81E-08	rs17651741	175.761	2.81E-08
rs1420098	498.3018	1.83E-20	rs17694108	342.065	1.21E-14	rs17694108	342.065	1.21E-14
rs1517352	334.6373	3.87E-14	rs17780256	249.7389	3.19E-11	rs17780256	249.7389	3.19E-11
rs1569328	213.5132	3.21E-09	rs181826	363.6198	4.05E-15	rs181826	363.6198	4.05E-15
rs17293632	479.1136	2.71E-20	rs1990760	247.5031	3.56E-10	rs1990760	247.5031	3.56E-10
rs17651741	175.761	2.81E-08	rs2024092	446.6902	1.12E-18	rs2024092	446.6902	1.12E-18
rs17694108	342.065	1.21E-14	rs2050392	264.4197	1.87E-11	rs2050392	264.4197	1.87E-11
rs17780256	249.7389	3.19E-11	rs2143178	1002.376	4.80E-38	rs2143178	1002.376	4.80E-38
rs181826	363.6198	4.05E-15	rs2153283	289.7066	1.54E-11	rs2153283	289.7066	1.54E-11
rs1847472	234.9589	6.63E-10	rs2270395	253.7596	5.17E-11	rs2270395	253.7596	5.17E-11
rs1990760	247.5031	3.56E-10	rs2274351	209.8084	6.93E-09	rs2274351	209.8084	6.93E-09
rs2024092	446.6902	1.12E-18	rs2297559	275.1224	1.88E-11	rs2297559	275.1224	1.88E-11
rs2050392	264.4197	1.87E-11	rs2328546	324.6926	1.30E-13	rs2328546	324.6926	1.30E-13
rs2143178	1002.376	4.80E-38	rs2395022	300.3769	8.27E-15	rs2395022	300.3769	8.27E-15
rs2153283	289.7066	1.54E-11	rs2488397	365.647	4.55E-16	rs2488397	365.647	4.55E-16
rs2270395	253.7596	5.17E-11	rs2497318	230.5212	1.36E-10	rs2497318	230.5212	1.36E-10
rs2274351	209.8084	6.93E-09	rs2538470	243.1882	3.00E-11	rs2538470	243.1882	3.00E-11
rs2297559	275.1224	1.88E-11	rs259964	260.6283	6.93E-12	rs259964	260.6283	6.93E-12
rs2328546	324.6926	1.30E-13	rs2688608	221.5746	2.75E-10	rs2688608	221.5746	2.75E-10
rs2395022	300.3769	8.27E-15	rs272882	1414.182	1.47E-52	rs272882	1414.182	1.47E-52
rs2488397	365.647	4.55E-16	rs2836883	1309.722	3.38E-48	rs2836883	1309.722	3.38E-48
rs2497318	230.5212	1.36E-10	rs2974935	272.3777	8.87E-12	rs2974935	272.3777	8.87E-12
rs2538470	243.1882	3.00E-11	rs3024493	1194.719	1.65E-50	rs3024493	1194.719	1.65E-50
rs259964	260.6283	6.93E-12	rs34856868	269.111	9.80E-09	rs34779708	599.9239	2.07E-25
rs2688608	221.5746	2.75E-10	rs35256947	299.2726	3.87E-13	rs34804116	180.5181	3.62E-08
rs272882	1414.182	1.47E-52	rs3776414	324.0317	2.65E-14	rs34856868	269.111	9.80E-09
rs2836883	1309.722	3.38E-48	rs3801835	214.0495	1.47E-09	rs35164067	518.4696	2.66E-20
rs2847278	646.4646	8.33E-28	rs4692386	187.0051	1.21E-08	rs35256947	299.2726	3.87E-13
rs2974935	272.3777	8.87E-12	rs4703855	244.486	7.16E-11	rs36048684	201.1767	3.70E-09
rs3024493	1194.719	1.65E-50	rs4743820	197.2914	3.80E-09	rs3776414	324.0317	2.65E-14
rs3184504	207.6845	1.29E-09	rs4795397	1108.149	8.30E-44	rs3801835	214.0495	1.47E-09
rs34779708	599.9239	2.07E-25	rs4976646	276.5565	3.23E-12	rs4692386	187.0051	1.21E-08

rs34804116	180.5181	3.62E-08	rs55808324	389.1114	5.13E-17	rs4703855	244.486	7.16E-11
rs34856868	269.111	9.80E-09	rs6058869	171.2594	2.63E-08	rs4743820	197.2914	3.80E-09
rs35164067	518.4696	2.66E-20	rs6062496	863.1491	2.11E-33	rs4795397	1108.149	8.30E-44
rs35256947	299.2726	3.87E-13	rs6074022	238.6478	8.32E-11	rs4976646	276.5565	3.23E-12
rs35730213	1196.342	8.33E-45	rs6111031	2188.355	1.23E-71	rs516246	327.8416	1.15E-13
rs36048684	201.1767	3.70E-09	rs62037363	537.0167	6.36E-22	rs55808324	389.1114	5.13E-17
rs367569	435.908	1.93E-17	rs62434177	231.6466	1.14E-08	rs559928	312.8361	3.33E-13
rs3776414	324.0317	2.65E-14	rs6456426	238.6816	8.18E-11	rs56167332	1263.744	7.17E-50
rs3801835	214.0495	1.47E-09	rs6466198	387.3862	2.18E-16	rs6058869	171.2594	2.63E-08
rs4692386	187.0051	1.21E-08	rs648541	217.9667	1.22E-09	rs6062496	863.1491	2.11E-33
rs4703855	244.486	7.16E-11	rs6500315	235.7909	1.12E-10	rs6074022	238.6478	8.32E-11
rs4743820	197.2914	3.80E-09	rs6561151	400.8148	3.53E-17	rs6111031	2188.355	1.23E-71
rs4795397	1108.149	8.30E-44	rs6584281	1578.787	9.36E-62	rs62037363	537.0167	6.36E-22
rs4976646	276.5565	3.23E-12	rs6708373	1042.071	1.43E-41	rs62434177	231.6466	1.14E-08
rs516246	327.8416	1.15E-13	rs6745185	216.9043	1.37E-09	rs6456426	238.6816	8.18E-11
rs55808324	389.1114	5.13E-17	rs67643815	227.0924	6.42E-10	rs6466198	387.3862	2.18E-16
rs559928	312.8361	3.33E-13	rs7011507	178.3419	2.03E-08	rs648541	217.9667	1.22E-09
rs56167332	1263.744	7.17E-50	rs7015630	177.2513	2.90E-08	rs6500315	235.7909	1.12E-10
rs6058869	171.2594	2.63E-08	rs7194886	905.8114	2.53E-36	rs6561151	400.8148	3.53E-17
rs6062496	863.1491	2.11E-33	rs7240004	240.3247	1.01E-10	rs6584281	1578.787	9.36E-62
rs6074022	238.6478	8.32E-11	rs72924296	184.9984	1.44E-08	rs6588248	386.6904	1.38E-16
rs6111031	2188.355	1.23E-71	rs7523442	894.7631	2.76E-36	rs6708373	1042.071	1.43E-41
rs62037363	537.0167	6.36E-22	rs769177	390.3316	6.53E-20	rs6745185	216.9043	1.37E-09
rs62434177	231.6466	1.14E-08	rs780094	338.2113	3.88E-15	rs67643815	227.0924	6.42E-10
rs6456426	238.6816	8.18E-11	rs7848647	892.3676	3.16E-35	rs7011507	178.3419	2.03E-08
rs6466198	387.3862	2.18E-16	rs78487399	351.9813	7.71E-16	rs7015630	177.2513	2.90E-08
rs648541	217.9667	1.22E-09	rs913678	243.6786	5.35E-11	rs71593329	353.0646	1.19E-14
rs6500315	235.7909	1.12E-10	rs941823	297.4595	6.19E-13	rs7194886	905.8114	2.53E-36
rs6561151	400.8148	3.53E-17	rs9457247	456.2102	2.48E-18	rs7240004	240.3247	1.01E-10
rs6584281	1578.787	9.36E-62	rs9557207	308.6561	3.52E-13	rs7253253	181.5822	6.19E-09
rs6588248	386.6904	1.38E-16	rs974801	287.8439	7.07E-13	rs72924296	184.9984	1.44E-08
rs6651252	215.2402	9.08E-10	rs9836291	1233.881	9.61E-53	rs744166	563.7361	1.14E-22
rs6708373	1042.071	1.43E-41	rs9889296	504.9946	1.35E-20	rs7523442	894.7631	2.76E-36
rs6740462	285.3672	5.59E-12				rs7547569	6336.042	1.65E-170
rs6745185	216.9043	1.37E-09				rs769177	390.3316	6.53E-20
rs67643815	227.0924	6.42E-10				rs780094	338.2113	3.88E-15
rs6933404	352.122	5.84E-15				rs7848647	892.3676	3.16E-35
rs7011507	178.3419	2.03E-08				rs78487399	351.9813	7.71E-16
rs7015630	177.2513	2.90E-08				rs913678	243.6786	5.35E-11
rs71593329	353.0646	1.19E-14				rs9264942	473.202	1.55E-18
rs7194886	905.8114	2.53E-36				rs941823	297.4595	6.19E-13
rs7240004	240.3247	1.01E-10				rs9457247	456.2102	2.48E-18
rs7253253	181.5822	6.19E-09				rs9557207	308.6561	3.52E-13
rs72634258	539.0976	1.25E-19				rs974801	287.8439	7.07E-13
rs72924296	184.9984	1.44E-08				rs9836291	1233.881	9.61E-53
rs744166	563.7361	1.14E-22				rs9889296	504.9946	1.35E-20
rs7523442	894.7631	2.76E-36						
rs7547569	6336.042	1.65E-170						
rs7608910	882.8453	2.60E-36						
rs7657746	321.1577	1.83E-13						
rs769177	390.3316	6.53E-20						
rs7773324	211.4368	5.84E-09						
rs780094	338.2113	3.88E-15						
rs7848647	892.3676	3.16E-35						
rs78487399	351.9813	7.71E-16						
rs913678	243.6786	5.35E-11						
rs9264942	473.202	1.55E-18						
rs9273363	1736.557	3.30E-58						
rs941823	297.4595	6.19E-13						
rs9457247	456.2102	2.48E-18						
rs9557207	308.6561	3.52E-13						
rs974801	287.8439	7.07E-13						
rs9836291	1233.881	9.61E-53						
rs9889296	504.9946	1.35E-20						

Supplementary Table 3. SNPs for IBD to OA and gout identified from GWAS analysis

IBD to OA_Knee			IBD to OA_Coxa			IBD to gout		
SNPs	*F*-statistics	genome-wide significance	SNPs	*F*-statistics	genome-wide significance	SNPs	*F*-statistics	genome-wide significance
rs10142466	193.8873	1.08E-08	rs10142466	193.8873	1.08E-08	rs10142466	193.8873	1.08E-08
rs10758669	1169.886	4.70E-48	rs10758669	1169.886	4.70E-48	rs10758669	1169.886	4.70E-48
rs10761659	1368.642	4.97E-53	rs10761659	1368.642	4.97E-53	rs10761659	1368.642	4.97E-53
rs10800309	911.3654	6.15E-37	rs10800309	911.3654	6.15E-37	rs10800309	911.3654	6.15E-37
rs10878302	193.3339	5.26E-09	rs10878302	193.3339	5.26E-09	rs10878302	193.3339	5.26E-09
rs10956252	379.5455	2.26E-16	rs10956252	379.5455	2.26E-16	rs10956252	379.5455	2.26E-16
rs11152949	554.949	7.25E-23	rs11152949	554.949	7.25E-23	rs11152949	554.949	7.25E-23
rs11230563	345.2953	1.71E-14	rs11230563	345.2953	1.71E-14	rs11230563	345.2953	1.71E-14
rs11236797	1308.07	9.32E-52	rs11236797	1308.07	9.32E-52	rs11236797	1308.07	9.32E-52
rs11641016	453.2093	9.51E-17	rs11641016	453.2093	9.51E-17	rs11641016	453.2093	9.51E-17
rs11677953	345.3509	2.92E-15	rs11677953	345.3509	2.92E-15	rs11677953	345.3509	2.92E-15
rs11691685	255.3177	7.27E-11	rs11691685	255.3177	7.27E-11	rs11691685	255.3177	7.27E-11
rs11713774	250.8475	3.92E-11	rs11713774	250.8475	3.92E-11	rs11713774	250.8475	3.92E-11
rs11793497	1387.172	1.71E-54	rs11793497	1387.172	1.71E-54	rs11793497	1387.172	1.71E-54
rs1182188	209.8552	1.08E-09	rs1182188	209.8552	1.08E-09	rs1182188	209.8552	1.08E-09
rs12318183	657.7615	1.67E-27	rs12318183	657.7615	1.67E-27	rs12318183	657.7615	1.67E-27
rs12411259	188.2763	6.18E-09	rs12411259	188.2763	6.18E-09	rs12411259	188.2763	6.18E-09
rs1250566	508.7193	4.77E-20	rs1250566	508.7193	4.77E-20	rs1250566	508.7193	4.77E-20
rs12585310	247.8039	5.25E-11	rs12585310	247.8039	5.25E-11	rs12585310	247.8039	5.25E-11
rs1267499	239.5031	5.22E-11	rs1267499	239.5031	5.22E-11	rs1267499	239.5031	5.22E-11
rs12718244	323.4647	3.35E-14	rs12718244	323.4647	3.35E-14	rs12718244	323.4647	3.35E-14
rs12722515	307.5065	4.57E-12	rs12722515	307.5065	4.57E-12	rs12722515	307.5065	4.57E-12
rs12796489	3051.119	2.87E-69	rs12796489	3051.119	2.87E-69	rs12796489	3051.119	2.87E-69
rs1292053	279.6435	9.89E-13	rs1292053	279.6435	9.89E-13	rs1292053	279.6435	9.89E-13
rs1297258	742.3875	5.38E-30	rs1297258	742.3875	5.38E-30	rs1297258	742.3875	5.38E-30
rs13107612	258.4067	1.62E-11	rs13107612	258.4067	1.62E-11	rs13107612	258.4067	1.62E-11
rs13204742	214.166	5.39E-10	rs13204742	214.166	5.39E-10	rs13204742	214.166	5.39E-10
rs13407913	476.5494	1.69E-20	rs13407913	476.5494	1.69E-20	rs13407913	476.5494	1.69E-20
rs1363907	373.9241	4.87E-15	rs1363907	373.9241	4.87E-15	rs1363907	373.9241	4.87E-15
rs1388585	404.0949	6.85E-22	rs1388585	404.0949	6.85E-22	rs1388585	404.0949	6.85E-22
rs1420098	498.3018	1.83E-20	rs1420098	498.3018	1.83E-20	rs1420098	498.3018	1.83E-20
rs1517352	334.6373	3.87E-14	rs1517352	334.6373	3.87E-14	rs1517352	334.6373	3.87E-14
rs1569328	213.5132	3.21E-09	rs1569328	213.5132	3.21E-09	rs1569328	213.5132	3.21E-09
rs17293632	479.1136	2.71E-20	rs17293632	479.1136	2.71E-20	rs17293632	479.1136	2.71E-20
rs17651741	175.761	2.81E-08	rs17651741	175.761	2.81E-08	rs17651741	175.761	2.81E-08
rs17694108	342.065	1.21E-14	rs17694108	342.065	1.21E-14	rs17694108	342.065	1.21E-14
rs17780256	249.7389	3.19E-11	rs17780256	249.7389	3.19E-11	rs17780256	249.7389	3.19E-11
rs181826	363.6198	4.05E-15	rs181826	363.6198	4.05E-15	rs181826	363.6198	4.05E-15
rs1847472	234.9589	6.63E-10	rs1847472	234.9589	6.63E-10	rs1847472	234.9589	6.63E-10
rs1990760	247.5031	3.56E-10	rs1990760	247.5031	3.56E-10	rs1990760	247.5031	3.56E-10
rs2024092	446.6902	1.12E-18	rs2024092	446.6902	1.12E-18	rs2024092	446.6902	1.12E-18
rs2050392	264.4197	1.87E-11	rs2050392	264.4197	1.87E-11	rs2050392	264.4197	1.87E-11
rs2143178	1002.376	4.80E-38	rs2143178	1002.376	4.80E-38	rs2143178	1002.376	4.80E-38
rs2153283	289.7066	1.54E-11	rs2153283	289.7066	1.54E-11	rs2153283	289.7066	1.54E-11
rs2270395	253.7596	5.17E-11	rs2270395	253.7596	5.17E-11	rs2270395	253.7596	5.17E-11
rs2274351	209.8084	6.93E-09	rs2274351	209.8084	6.93E-09	rs2274351	209.8084	6.93E-09
rs2297559	275.1224	1.88E-11	rs2297559	275.1224	1.88E-11	rs2297559	275.1224	1.88E-11
rs2328546	324.6926	1.30E-13	rs2328546	324.6926	1.30E-13	rs2328546	324.6926	1.30E-13
rs2395022	300.3769	8.27E-15	rs2395022	300.3769	8.27E-15	rs2395022	300.3769	8.27E-15
rs2488397	365.647	4.55E-16	rs2488397	365.647	4.55E-16	rs2488397	365.647	4.55E-16
rs2497318	230.5212	1.36E-10	rs2497318	230.5212	1.36E-10	rs2497318	230.5212	1.36E-10
rs2538470	243.1882	3.00E-11	rs2538470	243.1882	3.00E-11	rs2538470	243.1882	3.00E-11
rs259964	260.6283	6.93E-12	rs259964	260.6283	6.93E-12	rs259964	260.6283	6.93E-12
rs2688608	221.5746	2.75E-10	rs2688608	221.5746	2.75E-10	rs2688608	221.5746	2.75E-10
rs272882	1414.182	1.47E-52	rs272882	1414.182	1.47E-52	rs272882	1414.182	1.47E-52
rs2836883	1309.722	3.38E-48	rs2836883	1309.722	3.38E-48	rs2836883	1309.722	3.38E-48
rs2847278	646.4646	8.33E-28	rs2847278	646.4646	8.33E-28	rs2847278	646.4646	8.33E-28
rs2974935	272.3777	8.87E-12	rs2974935	272.3777	8.87E-12	rs2974935	272.3777	8.87E-12
rs3024493	1194.719	1.65E-50	rs3024493	1194.719	1.65E-50	rs3024493	1194.719	1.65E-50
rs3184504	207.6845	1.29E-09	rs3184504	207.6845	1.29E-09	rs3184504	207.6845	1.29E-09
rs34779708	599.9239	2.07E-25	rs34779708	599.9239	2.07E-25	rs34779708	599.9239	2.07E-25

rs34804116	180.5181	3.62E-08	rs34804116	180.5181	3.62E-08	rs34804116	180.5181	3.62E-08
rs34856868	269.111	9.80E-09	rs34856868	269.111	9.80E-09	rs34856868	269.111	9.80E-09
rs35164067	518.4696	2.66E-20	rs35164067	518.4696	2.66E-20	rs35164067	518.4696	2.66E-20
rs35256947	299.2726	3.87E-13	rs35256947	299.2726	3.87E-13	rs35256947	299.2726	3.87E-13
rs35730213	1196.342	8.33E-45	rs35730213	1196.342	8.33E-45	rs35730213	1196.342	8.33E-45
rs36048684	201.1767	3.70E-09	rs36048684	201.1767	3.70E-09	rs36048684	201.1767	3.70E-09
rs367569	435.908	1.93E-17	rs367569	435.908	1.93E-17	rs367569	435.908	1.93E-17
rs3776414	324.0317	2.65E-14	rs3776414	324.0317	2.65E-14	rs3776414	324.0317	2.65E-14
rs3801835	214.0495	1.47E-09	rs3801835	214.0495	1.47E-09	rs3801835	214.0495	1.47E-09
rs4692386	187.0051	1.21E-08	rs4692386	187.0051	1.21E-08	rs4692386	187.0051	1.21E-08
rs4703855	244.486	7.16E-11	rs4703855	244.486	7.16E-11	rs4703855	244.486	7.16E-11
rs4743820	197.2914	3.80E-09	rs4743820	197.2914	3.80E-09	rs4743820	197.2914	3.80E-09
rs4795397	1108.149	8.30E-44	rs4795397	1108.149	8.30E-44	rs4795397	1108.149	8.30E-44
rs4976646	276.5565	3.23E-12	rs4976646	276.5565	3.23E-12	rs4976646	276.5565	3.23E-12
rs516246	327.8416	1.15E-13	rs516246	327.8416	1.15E-13	rs516246	327.8416	1.15E-13
rs55808324	389.1114	5.13E-17	rs55808324	389.1114	5.13E-17	rs55808324	389.1114	5.13E-17
rs559928	312.8361	3.33E-13	rs559928	312.8361	3.33E-13	rs559928	312.8361	3.33E-13
rs56167332	1263.744	7.17E-50	rs56167332	1263.744	7.17E-50	rs56167332	1263.744	7.17E-50
rs6058869	171.2594	2.63E-08	rs6058869	171.2594	2.63E-08	rs6058869	171.2594	2.63E-08
rs6062496	863.1491	2.11E-33	rs6062496	863.1491	2.11E-33	rs6062496	863.1491	2.11E-33
rs6074022	238.6478	8.32E-11	rs6074022	238.6478	8.32E-11	rs6074022	238.6478	8.32E-11
rs6111031	2188.355	1.23E-71	rs6111031	2188.355	1.23E-71	rs6111031	2188.355	1.23E-71
rs62037363	537.0167	6.36E-22	rs62037363	537.0167	6.36E-22	rs62037363	537.0167	6.36E-22
rs62434177	231.6466	1.14E-08	rs62434177	231.6466	1.14E-08	rs62434177	231.6466	1.14E-08
rs6456426	238.6816	8.18E-11	rs6456426	238.6816	8.18E-11	rs6456426	238.6816	8.18E-11
rs6466198	387.3862	2.18E-16	rs6466198	387.3862	2.18E-16	rs6466198	387.3862	2.18E-16
rs648541	217.9667	1.22E-09	rs648541	217.9667	1.22E-09	rs648541	217.9667	1.22E-09
rs6500315	235.7909	1.12E-10	rs6500315	235.7909	1.12E-10	rs6500315	235.7909	1.12E-10
rs6561151	400.8148	3.53E-17	rs6561151	400.8148	3.53E-17	rs6561151	400.8148	3.53E-17
rs6584281	1578.787	9.36E-62	rs6584281	1578.787	9.36E-62	rs6584281	1578.787	9.36E-62
rs6588248	386.6904	1.38E-16	rs6588248	386.6904	1.38E-16	rs6588248	386.6904	1.38E-16
rs6651252	215.2402	9.08E-10	rs6651252	215.2402	9.08E-10	rs6651252	215.2402	9.08E-10
rs6708373	1042.071	1.43E-41	rs6708373	1042.071	1.43E-41	rs6708373	1042.071	1.43E-41
rs6740462	285.3672	5.59E-12	rs6740462	285.3672	5.59E-12	rs6740462	285.3672	5.59E-12
rs6745185	216.9043	1.37E-09	rs6745185	216.9043	1.37E-09	rs6745185	216.9043	1.37E-09
rs67643815	227.0924	6.42E-10	rs67643815	227.0924	6.42E-10	rs67643815	227.0924	6.42E-10
rs6933404	352.122	5.84E-15	rs6933404	352.122	5.84E-15	rs6933404	352.122	5.84E-15
rs7011507	178.3419	2.03E-08	rs7011507	178.3419	2.03E-08	rs7011507	178.3419	2.03E-08
rs7015630	177.2513	2.90E-08	rs7015630	177.2513	2.90E-08	rs7015630	177.2513	2.90E-08
rs71593329	353.0646	1.19E-14	rs71593329	353.0646	1.19E-14	rs71593329	353.0646	1.19E-14
rs7194886	905.8114	2.53E-36	rs7194886	905.8114	2.53E-36	rs7194886	905.8114	2.53E-36
rs7240004	240.3247	1.01E-10	rs7240004	240.3247	1.01E-10	rs7240004	240.3247	1.01E-10
rs7253253	181.5822	6.19E-09	rs7253253	181.5822	6.19E-09	rs7253253	181.5822	6.19E-09
rs72634258	539.0976	1.25E-19	rs72634258	539.0976	1.25E-19	rs72634258	539.0976	1.25E-19
rs72924296	184.9984	1.44E-08	rs72924296	184.9984	1.44E-08	rs72924296	184.9984	1.44E-08
rs744166	563.7361	1.14E-22	rs744166	563.7361	1.14E-22	rs744166	563.7361	1.14E-22
rs7523442	894.7631	2.76E-36	rs7523442	894.7631	2.76E-36	rs7523442	894.7631	2.76E-36
rs7547569	6336.042	1.65E-170	rs7547569	6336.042	1.65E-170	rs7547569	6336.042	1.65E-170
rs7608910	882.8453	2.60E-36	rs7608910	882.8453	2.60E-36	rs7608910	882.8453	2.60E-36
rs7657746	321.1577	1.83E-13	rs7657746	321.1577	1.83E-13	rs7657746	321.1577	1.83E-13
rs769177	390.3316	6.53E-20	rs769177	390.3316	6.53E-20	rs769177	390.3316	6.53E-20
rs7773324	211.4368	5.84E-09	rs7773324	211.4368	5.84E-09	rs7773324	211.4368	5.84E-09
rs780094	338.2113	3.88E-15	rs780094	338.2113	3.88E-15	rs780094	338.2113	3.88E-15
rs7848647	892.3676	3.16E-35	rs7848647	892.3676	3.16E-35	rs7848647	892.3676	3.16E-35
rs78487399	351.9813	7.71E-16	rs78487399	351.9813	7.71E-16	rs78487399	351.9813	7.71E-16
rs913678	243.6786	5.35E-11	rs913678	243.6786	5.35E-11	rs913678	243.6786	5.35E-11
rs9264942	473.202	1.55E-18	rs9264942	473.202	1.55E-18	rs9264942	473.202	1.55E-18
rs9273363	1736.557	3.30E-58	rs9273363	1736.557	3.30E-58	rs9273363	1736.557	3.30E-58
rs941823	297.4595	6.19E-13	rs941823	297.4595	6.19E-13	rs941823	297.4595	6.19E-13
rs9457247	456.2102	2.48E-18	rs9457247	456.2102	2.48E-18	rs9457247	456.2102	2.48E-18
rs9557207	308.6561	3.52E-13	rs9557207	308.6561	3.52E-13	rs9557207	308.6561	3.52E-13
rs974801	287.8439	7.07E-13	rs974801	287.8439	7.07E-13	rs974801	287.8439	7.07E-13
rs9836291	1233.881	9.61E-53	rs9836291	1233.881	9.61E-53	rs9836291	1233.881	9.61E-53
rs9889296	504.9946	1.35E-20	rs9889296	504.9946	1.35E-20	rs9889296	504.9946	1.35E-20

Supplementary Table 4. SNPs for IBD to ReA and PA identified from GWAS analysis

IBD to ReA			IBD to PA		
SNPs	*F*-statistics	genome-wide significance	SNPs	*F*-statistics	genome-wide significance
rs10758669	1169.886	4.70E-48	rs10142466	v	1.08E-08
rs10761659	1368.642	4.97E-53	rs10758669	1169.886	4.70E-48
rs10800309	911.3654	6.15E-37	rs10761659	1368.642	4.97E-53
rs10878302	193.3339	5.26E-09	rs10800309	911.3654	6.15E-37
rs10956252	379.5455	2.26E-16	rs10878302	193.3339	5.26E-09
rs11152949	554.949	7.25E-23	rs10956252	379.5455	2.26E-16
rs11230563	345.2953	1.71E-14	rs11152949	554.949	7.25E-23
rs11236797	1308.07	9.32E-52	rs11230563	345.2953	1.71E-14
rs11641016	453.2093	9.51E-17	rs11236797	1308.07	9.32E-52
rs11677953	345.3509	2.92E-15	rs11641016	453.2093	9.51E-17
rs11691685	255.3177	7.27E-11	rs11677953	345.3509	2.92E-15
rs11713774	250.8475	3.92E-11	rs11691685	255.3177	7.27E-11
rs11793497	1387.172	1.71E-54	rs11713774	250.8475	3.92E-11
rs1182188	209.8552	1.08E-09	rs11793497	1387.172	1.71E-54
rs12318183	657.7615	1.67E-27	rs1182188	209.8552	1.08E-09
rs12585310	247.8039	5.25E-11	rs12318183	657.7615	1.67E-27
rs1267499	239.5031	5.22E-11	rs12411259	188.2763	6.18E-09
rs12718244	323.4647	3.35E-14	rs1250566	508.7193	4.77E-20
rs12722515	307.5065	4.57E-12	rs12585310	247.8039	5.25E-11
rs12796489	3051.119	2.87E-69	rs1267499	239.5031	5.22E-11
rs1292053	279.6435	9.89E-13	rs12718244	323.4647	3.35E-14
rs1297258	742.3875	5.38E-30	rs12722515	307.5065	4.57E-12
rs13107612	258.4067	1.62E-11	rs12796489	3051.119	2.87E-69
rs13204742	214.166	5.39E-10	rs1292053	279.6435	9.89E-13
rs13407913	476.5494	1.69E-20	rs1297258	742.3875	5.38E-30
rs1388585	404.0949	6.85E-22	rs13107612	258.4067	1.62E-11
rs1569328	213.5132	3.21E-09	rs13204742	214.166	5.39E-10
rs17293632	479.1136	2.71E-20	rs13407913	476.5494	1.69E-20
rs17651741	175.761	2.81E-08	rs1363907	373.9241	4.87E-15
rs17694108	342.065	1.21E-14	rs1388585	404.0949	6.85E-22
rs17780256	249.7389	3.19E-11	rs1420098	498.3018	1.83E-20
rs181826	363.6198	4.05E-15	rs1517352	334.6373	3.87E-14
rs2024092	446.6902	1.12E-18	rs1569328	213.5132	3.21E-09
rs2050392	264.4197	1.87E-11	rs17293632	479.1136	2.71E-20
rs2143178	1002.376	4.80E-38	rs17651741	175.761	2.81E-08
rs2153283	289.7066	1.54E-11	rs17694108	342.065	1.21E-14
rs2270395	253.7596	5.17E-11	rs17780256	249.7389	3.19E-11
rs2274351	209.8084	6.93E-09	rs181826	363.6198	4.05E-15
rs2297559	275.1224	1.88E-11	rs1847472	234.9589	6.63E-10
rs2328546	324.6926	1.30E-13	rs1990760	247.5031	3.56E-10
rs2395022	300.3769	8.27E-15	rs2024092	446.6902	1.12E-18
rs2488397	365.647	4.55E-16	rs2050392	264.4197	1.87E-11
rs2538470	243.1882	3.00E-11	rs2143178	1002.376	4.80E-38
rs259964	260.6283	6.93E-12	rs2153283	289.7066	1.54E-11
rs272882	1414.182	1.47E-52	rs2270395	253.7596	5.17E-11
rs2836883	1309.722	3.38E-48	rs2274351	209.8084	6.93E-09
rs2974935	272.3777	8.87E-12	rs2297559	275.1224	1.88E-11
rs3024493	1194.719	1.65E-50	rs2328546	324.6926	1.30E-13
rs34779708	599.9239	2.07E-25	rs2395022	300.3769	8.27E-15
rs34804116	180.5181	3.62E-08	rs2488397	365.647	4.55E-16
rs34856868	269.111	9.80E-09	rs2497318	230.5212	1.36E-10
rs35256947	299.2726	3.87E-13	rs2538470	243.1882	3.00E-11
rs3776414	324.0317	2.65E-14	rs259964	260.6283	6.93E-12
rs3801835	214.0495	1.47E-09	rs2688608	221.5746	2.75E-10
rs4692386	187.0051	1.21E-08	rs272882	1414.182	1.47E-52
rs4703855	244.486	7.16E-11	rs2836883	1309.722	3.38E-48
rs4743820	197.2914	3.80E-09	rs2847278	646.4646	8.33E-28
rs4795397	1108.149	8.30E-44	rs2974935	272.3777	8.87E-12
rs4976646	276.5565	3.23E-12	rs3024493	1194.719	1.65E-50
rs55808324	389.1114	5.13E-17	rs3184504	207.6845	1.29E-09
rs6058869	171.2594	2.63E-08	rs34779708	599.9239	2.07E-25

rs6062496	863.1491	2.11E-33	rs34804116	180.5181	3.62E-08
rs6074022	238.6478	8.32E-11	rs34856868	269.111	9.80E-09
rs6111031	2188.355	1.23E-71	rs35164067	518.4696	2.66E-20
rs62037363	537.0167	6.36E-22	rs35256947	299.2726	3.87E-13
rs62434177	231.6466	1.14E-08	rs35730213	1196.342	8.33E-45
rs6456426	238.6816	8.18E-11	rs36048684	201.1767	3.70E-09
rs6466198	387.3862	2.18E-16	rs367569	435.908	1.93E-17
rs648541	217.9667	1.22E-09	rs3776414	324.0317	2.65E-14
rs6500315	235.7909	1.12E-10	rs3801835	214.0495	1.47E-09
rs6561151	400.8148	3.53E-17	rs4692386	187.0051	1.21E-08
rs6584281	1578.787	9.36E-62	rs4703855	244.486	7.16E-11
rs6708373	1042.071	1.43E-41	rs4743820	197.2914	3.80E-09
rs6745185	216.9043	1.37E-09	rs4795397	1108.149	8.30E-44
rs67643815	227.0924	6.42E-10	rs4976646	276.5565	3.23E-12
rs7011507	178.3419	2.03E-08	rs516246	327.8416	1.15E-13
rs7015630	177.2513	2.90E-08	rs55808324	389.1114	5.13E-17
rs7194886	905.8114	2.53E-36	rs559928	312.8361	3.33E-13
rs7240004	240.3247	1.01E-10	rs56167332	1263.744	7.17E-50
rs72924296	184.9984	1.44E-08	rs6058869	171.2594	2.63E-08
rs7523442	894.7631	2.76E-36	rs6062496	863.1491	2.11E-33
rs769177	390.3316	6.53E-20	rs6074022	238.6478	8.32E-11
rs780094	338.2113	3.88E-15	rs6111031	2188.355	1.23E-71
rs7848647	892.3676	3.16E-35	rs62037363	537.0167	6.36E-22
rs913678	243.6786	5.35E-11	rs62434177	231.6466	1.14E-08
rs941823	297.4595	6.19E-13	rs6456426	238.6816	8.18E-11
rs9457247	456.2102	2.48E-18	rs6466198	387.3862	2.18E-16
rs9557207	308.6561	3.52E-13	rs648541	217.9667	1.22E-09
rs974801	287.8439	7.07E-13	rs6500315	235.7909	1.12E-10
rs9836291	1233.881	9.61E-53	rs6561151	400.8148	3.53E-17
rs9889296	504.9946	1.35E-20	rs6584281	1578.787	9.36E-62
			rs6588248	386.6904	1.38E-16
			rs6651252	215.2402	9.08E-10
			rs6708373	1042.071	1.43E-41
			rs6740462	285.3672	5.59E-12
			rs6745185	216.9043	1.37E-09
			rs67643815	227.0924	6.42E-10
			rs6933404	352.122	5.84E-15
			rs7011507	178.3419	2.03E-08
			rs7015630	177.2513	2.90E-08
			rs71593329	353.0646	1.19E-14
			rs7194886	905.8114	2.53E-36
			rs7240004	240.3247	1.01E-10
			rs7253253	181.5822	6.19E-09
			rs72634258	539.0976	1.25E-19
			rs72924296	184.9984	1.44E-08
			rs744166	563.7361	1.14E-22
			rs7523442	894.7631	2.76E-36
			rs7547569	6336.042	1.65E-170
			rs7608910	882.8453	2.60E-36
			rs7657746	321.1577	1.83E-13
			rs769177	390.3316	6.53E-20
			rs7773324	211.4368	5.84E-09
			rs780094	338.2113	3.88E-15
			rs7848647	892.3676	3.16E-35
			rs78487399	351.9813	7.71E-16
			rs913678	243.6786	5.35E-11
			rs9264942	473.202	1.55E-18
			rs9273363	1736.557	3.30E-58
			rs941823	297.4595	6.19E-13
			rs9457247	456.2102	2.48E-18
			rs9557207	308.6561	3.52E-13
			rs974801	287.8439	7.07E-13
			rs9836291	1233.881	9.61E-53
			rs9889296	504.9946	1.35E-20

Supplementary Table 5. SNPs for CD to RA, AS and PSA identified from GWAS analysis

CD to RA			CD to AS			CD to PSA		
SNPs	*F*-statistics	genome-wide significance	SNPs	*F*-statistics	genome-wide significance	SNPs	*F*-statistics	genome-wide significance
rs10758669	1185.638	4.19E-34	rs10758669	1185.638	4.19E-34	rs10758669	1185.638	4.19E-34
rs10798069	285.7057	4.25E-09	rs10798069	285.7057	4.25E-09	rs10798069	285.7057	4.25E-09
rs10800309	425.0198	8.48E-13	rs10800309	425.0198	8.48E-13	rs10800309	425.0198	8.48E-13
rs10878302	376.5016	4.20E-11	rs10878302	376.5016	4.20E-11	rs10878302	376.5016	4.20E-11
rs10956252	769.1961	8.34E-22	rs10956252	769.1961	8.34E-22	rs10956252	769.1961	8.34E-22
rs10995271	1961.281	4.92E-53	rs10995271	1961.281	4.92E-53	rs10995271	1961.281	4.92E-53
rs11117431	819.5031	1.09E-19	rs11117431	819.5031	1.09E-19	rs11117431	819.5031	1.09E-19
rs11152949	903.5258	2.18E-25	rs11152949	903.5258	2.18E-25	rs11152949	903.5258	2.18E-25
rs11159833	441.3087	7.59E-14	rs11159833	441.3087	7.59E-14	rs11159833	441.3087	7.59E-14
rs11236797	1886.722	8.54E-51	rs11236797	1886.722	8.54E-51	rs11236797	1886.722	8.54E-51
rs11691685	423.9053	1.35E-11	rs11691685	423.9053	1.35E-11	rs11691685	423.9053	1.35E-11
rs11713774	497.6669	1.09E-14	rs11713774	497.6669	1.09E-14	rs11713774	497.6669	1.09E-14
rs11793497	1625.531	9.80E-44	rs11793497	1625.531	9.80E-44	rs11793497	1625.531	9.80E-44
rs12411259	763.1321	1.43E-22	rs1267501	269.1763	9.69E-09	rs1267501	269.1763	9.69E-09
rs1250573	1006.623	5.86E-26	rs12694846	591.3455	2.50E-17	rs12694846	591.3455	2.50E-17
rs1267501	269.1763	9.69E-09	rs12796489	3315.756	4.96E-51	rs12796489	3315.756	4.96E-51
rs12694846	591.3455	2.50E-17	rs1292053	473.9219	1.75E-14	rs1292053	473.9219	1.75E-14
rs12796489	3315.756	4.96E-51	rs1297258	917.8748	2.11E-25	rs12949918	611.088	3.47E-17
rs1292053	473.9219	1.75E-14	rs13407913	744.6965	9.64E-22	rs1297258	917.8748	2.11E-25
rs12949918	611.088	3.47E-17	rs1456896	472.4196	1.03E-13	rs13407913	744.6965	9.64E-22
rs1297258	917.8748	2.11E-25	rs1569328	389.0255	6.47E-11	rs1363907	593.4012	3.89E-16
rs13001325	818.3211	1.68E-22	rs17129991	410.3462	2.81E-10	rs1456896	472.4196	1.03E-13
rs13407913	744.6965	9.64E-22	rs17293632	688.8775	3.70E-20	rs1569328	389.0255	6.47E-11
rs1363907	593.4012	3.89E-16	rs17622378	2062.584	7.17E-56	rs17129991	410.3462	2.81E-10
rs1456896	472.4196	1.03E-13	rs17694108	294.4707	3.29E-09	rs17293632	688.8775	3.70E-20
rs1517352	353.2318	1.31E-10	rs181826	536.628	4.53E-15	rs17388425	769.2465	6.54E-20
rs1569328	389.0255	6.47E-11	rs1927681	449.4354	2.42E-13	rs17391694	351.9615	2.62E-09
rs1646019	606.1476	8.62E-17	rs2024092	856.7346	7.13E-25	rs17622378	2062.584	7.17E-56
rs17129991	410.3462	2.81E-10	rs2153283	464.8112	2.39E-12	rs17694108	294.4707	3.29E-09
rs17293632	688.8775	3.70E-20	rs2270395	649.5767	8.93E-18	rs181826	536.628	4.53E-15
rs17388425	769.2465	6.54E-20	rs2395022	285.6961	3.13E-10	rs1927681	449.4354	2.42E-13
rs17391694	351.9615	2.62E-09	rs2413583	1423.934	7.72E-36	rs2024092	856.7346	7.13E-25
rs17622378	2062.584	7.17E-56	rs2538470	299.5613	1.05E-09	rs2153283	464.8112	2.39E-12
rs17694108	294.4707	3.29E-09	rs259964	291.5153	2.08E-09	rs2227551	451.6324	4.72E-13
rs181826	536.628	4.53E-15	rs2641348	326.5512	9.65E-10	rs2270395	649.5767	8.93E-18
rs1847472	378.8982	1.09E-10	rs26528	826.3572	1.29E-22	rs2284553	595.7384	5.63E-17
rs1927681	449.4354	2.42E-13	rs2974935	329.3259	5.80E-10	rs2395022	285.6961	3.13E-10
rs2024092	856.7346	7.13E-25	rs3024505	839.8039	3.95E-25	rs2413583	1423.934	7.72E-36
rs212388	579.723	1.80E-16	rs303429	322.3447	8.38E-10	rs2538470	299.5613	1.05E-09
rs2153283	464.8112	2.39E-12	rs3197999	1130.277	2.05E-33	rs259964	291.5153	2.08E-09
rs2227551	451.6324	4.72E-13	rs34779708	955.9602	1.90E-27	rs2641348	326.5512	9.65E-10
rs2270395	649.5767	8.93E-18	rs34787213	624.9867	2.85E-16	rs26528	826.3572	1.29E-22
rs2284553	595.7384	5.63E-17	rs34804116	483.3849	1.27E-13	rs2974935	329.3259	5.80E-10
rs2395022	285.6961	3.13E-10	rs35320439	349.1255	9.89E-10	rs3024505	839.8039	3.95E-25
rs2413583	1423.934	7.72E-36	rs3776414	427.5481	5.04E-13	rs303429	322.3447	8.38E-10
rs2538470	299.5613	1.05E-09	rs3801810	456.9041	6.63E-14	rs3197999	1130.277	2.05E-33
rs259964	291.5153	2.08E-09	rs438475	672.0539	3.42E-20	rs34779708	955.9602	1.90E-27
rs2641348	326.5512	9.65E-10	rs4703855	259.8926	3.03E-08	rs34787213	624.9867	2.85E-16
rs26528	826.3572	1.29E-22	rs4795397	1013.206	3.84E-28	rs34804116	483.3849	1.27E-13
rs2847293	866.4858	6.14E-26	rs56163845	417.0559	9.40E-12	rs35164067	773.1118	3.19E-20
rs28999107	417.3493	1.29E-11	rs6062496	819.4354	3.82E-22	rs35320439	349.1255	9.89E-10
rs2974935	329.3259	5.80E-10	rs6074022	402.0877	2.70E-12	rs3776414	427.5481	5.04E-13
rs3024505	839.8039	3.95E-25	rs6111031	2508.531	9.61E-55	rs3801810	456.9041	6.63E-14
rs303429	322.3447	8.38E-10	rs61839660	414.5495	3.19E-13	rs438475	672.0539	3.42E-20
rs3129871	400.5714	1.80E-11	rs640466	310.4759	1.31E-09	rs4703855	259.8926	3.03E-08
rs3184504	263.8778	1.71E-08	rs6456426	568.7683	1.37E-16	rs4795397	1013.206	3.84E-28
rs3197999	1130.277	2.05E-33	rs6500315	855.5855	2.18E-23	rs516246	756.6226	1.33E-20
rs34779708	955.9602	1.90E-27	rs6561151	871.4024	4.68E-25	rs559928	345.1139	3.75E-10
rs34787213	624.9867	2.85E-16	rs6702421	490.1676	6.53E-15	rs56163845	417.0559	9.40E-12
rs34804116	483.3849	1.27E-13	rs6738394	345.5531	8.98E-11	rs6062496	819.4354	3.82E-22
rs35164067	773.1118	3.19E-20	rs6738490	3011.015	4.26E-78	rs6074022	402.0877	2.70E-12

rs35320439	349.1255	9.89E-10	rs7015630	319.6113	9.00E-10	rs6111031	2508.531	9.61E-55
rs35730213	1070.897	7.84E-28	rs7085798	1773.754	1.53E-47	rs61839660	414.5495	3.19E-13
rs36016881	396.5737	1.60E-10	rs71624119	361.1734	6.57E-10	rs640466	310.4759	1.31E-09
rs3776414	427.5481	5.04E-13	rs7194886	2991.268	1.42E-77	rs6456426	568.7683	1.37E-16
rs3801810	456.9041	6.63E-14	rs7236492	298.1178	9.09E-09	rs6500315	855.5855	2.18E-23
rs438475	672.0539	3.42E-20	rs72727394	394.8176	5.28E-12	rs6561151	871.4024	4.68E-25
rs4703855	259.8926	3.03E-08	rs727563	316.5484	1.88E-10	rs6702421	490.1676	6.53E-15
rs4795397	1013.206	3.84E-28	rs7438704	372.882	3.42E-11	rs6738394	345.5531	8.98E-11
rs516246	756.6226	1.33E-20	rs76906269	665.0524	1.75E-26	rs6738490	3011.015	4.26E-78
rs559928	345.1139	3.75E-10	rs780094	751.0683	4.56E-22	rs6908425	422.5766	4.81E-12
rs56163845	417.0559	9.40E-12	rs7848647	1017.738	1.55E-27	rs7015630	319.6113	9.00E-10
rs6062496	819.4354	3.82E-22	rs7969592	308.2446	1.04E-09	rs7085798	1773.754	1.53E-47
rs6074022	402.0877	2.70E-12	rs915286	253.6819	2.59E-08	rs71624119	361.1734	6.57E-10
rs6111031	2508.531	9.61E-55	rs9491892	559.253	3.80E-17	rs7194886	2991.268	1.42E-77
rs61839660	414.5495	3.19E-13	rs9554587	363.6165	8.29E-11	rs7236492	298.1178	9.09E-09
rs640466	310.4759	1.31E-09	rs9594766	311.0598	1.39E-09	rs72727394	394.8176	5.28E-12
rs6456426	568.7683	1.37E-16	rs9889296	941.4874	2.96E-25	rs727563	316.5484	1.88E-10
rs6500315	855.5855	2.18E-23				rs7438704	372.882	3.42E-11
rs6561151	871.4024	4.68E-25				rs7517847	6754.072	1.38E-159
rs6651252	581.0403	3.86E-16				rs76906269	665.0524	1.75E-26
rs6679677	698.7974	4.67E-17				rs7786444	297.9321	9.83E-10
rs6702421	490.1676	6.53E-15				rs77981966	520.0566	2.19E-16
rs6738394	345.5531	8.98E-11				rs780094	751.0683	4.56E-22
rs6738490	3011.015	4.26E-78				rs7848647	1017.738	1.55E-27
rs6740462	444.604	1.74E-12				rs7969592	308.2446	1.04E-09
rs6827756	337.2986	3.27E-10				rs915286	253.6819	2.59E-08
rs6908425	422.5766	4.81E-12				rs9264942	1207.646	6.78E-32
rs7015630	319.6113	9.00E-10				rs9457247	881.0207	2.08E-23
rs7085798	1773.754	1.53E-47				rs9491892	559.253	3.80E-17
rs71624119	361.1734	6.57E-10				rs9554587	363.6165	8.29E-11
rs7194886	2991.268	1.42E-77				rs9594766	311.0598	1.39E-09
rs7236492	298.1178	9.09E-09				rs9889296	941.4874	2.96E-25
rs72727394	394.8176	5.28E-12						
rs727563	316.5484	1.88E-10						
rs7438704	372.882	3.42E-11						
rs7517847	6754.072	1.38E-159						
rs7608910	802.6889	2.95E-23						
rs76906269	665.0524	1.75E-26						
rs7773324	343.4652	1.06E-09						
rs7786444	297.9321	9.83E-10						
rs77981966	520.0566	2.19E-16						
rs780094	751.0683	4.56E-22						
rs7848647	1017.738	1.55E-27						
rs7969592	308.2446	1.04E-09						
rs915286	253.6819	2.59E-08						
rs9264942	1207.646	6.78E-32						
rs9457247	881.0207	2.08E-23						
rs9491892	559.253	3.80E-17						
rs9554587	363.6164	8.29E-11						
rs9594766	311.0598	1.39E-09						
rs9889296	941.4874	2.96E-25						

Supplementary Table 6. SNPs for CD to OA and gout identified from GWAS analysis

CD to OA_Knee			CD to OA_Coxa			CD to gout		
SNPs	*F*-statistics	genome-wide significance	SNPs	*F*-statistics	genome-wide significance	SNPs	*F*-statistics	genome-wide significance
rs10758669	1185.638	4.19E-34	rs10758669	1185.638	4.19E-34	rs10758669	1185.638	4.19E-34
rs10798069	285.7057	4.25E-09	rs10798069	285.7057	4.25E-09	rs10798069	285.7057	4.25E-09
rs10800309	425.0198	8.48E-13	rs10800309	425.0198	8.48E-13	rs10800309	425.0198	8.48E-13
rs10878302	376.5016	4.20E-11	rs10878302	376.5016	4.20E-11	rs10878302	376.5016	4.20E-11
rs10956252	769.1961	8.34E-22	rs10956252	769.1961	8.34E-22	rs10956252	769.1961	8.34E-22
rs10995271	1961.281	4.92E-53	rs10995271	1961.281	4.92E-53	rs10995271	1961.281	4.92E-53
rs11117431	819.5031	1.09E-19	rs11117431	819.5031	1.09E-19	rs11117431	819.5031	1.09E-19
rs11152949	903.5258	2.18E-25	rs11152949	903.5258	2.18E-25	rs11152949	903.5258	2.18E-25
rs11159833	441.3087	7.59E-14	rs11159833	441.3087	7.59E-14	rs11159833	441.3087	7.59E-14
rs11236797	1886.722	8.54E-51	rs11236797	1886.722	8.54E-51	rs11236797	1886.722	8.54E-51
rs11691685	423.9053	1.35E-11	rs11691685	423.9053	1.35E-11	rs11691685	423.9053	1.35E-11
rs11713774	497.6669	1.09E-14	rs11713774	497.6669	1.09E-14	rs11713774	497.6669	1.09E-14
rs11793497	1625.531	9.80E-44	rs11793497	1625.531	9.80E-44	rs11793497	1625.531	9.80E-44
rs12411259	763.1321	1.43E-22	rs12411259	763.1321	1.43E-22	rs12411259	763.1321	1.43E-22
rs1250573	1006.623	5.86E-26	rs1250573	1006.623	5.86E-26	rs1250573	1006.623	5.86E-26
rs1267501	269.1763	9.69E-09	rs1267501	269.1763	9.69E-09	rs1267501	269.1763	9.69E-09
rs12694846	591.3455	2.50E-17	rs12694846	591.3455	2.50E-17	rs12694846	591.3455	2.50E-17
rs12796489	3315.756	4.96E-51	rs12796489	3315.756	4.96E-51	rs12796489	3315.756	4.96E-51
rs1292053	473.9219	1.75E-14	rs1292053	473.9219	1.75E-14	rs1292053	473.9219	1.75E-14
rs12949918	611.088	3.47E-17	rs12949918	611.088	3.47E-17	rs12949918	611.088	3.47E-17
rs1297258	917.8748	2.11E-25	rs1297258	917.8748	2.11E-25	rs1297258	917.8748	2.11E-25
rs13001325	818.3211	1.68E-22	rs13001325	818.3211	1.68E-22	rs13001325	818.3211	1.68E-22
rs13407913	744.6965	9.64E-22	rs13407913	744.6965	9.64E-22	rs13407913	744.6965	9.64E-22
rs1363907	593.4012	3.89E-16	rs1363907	593.4012	3.89E-16	rs1363907	593.4012	3.89E-16
rs1456896	472.4196	1.03E-13	rs1456896	472.4196	1.03E-13	rs1456896	472.4196	1.03E-13
rs1517352	353.2318	1.31E-10	rs1517352	353.2318	1.31E-10	rs1517352	353.2318	1.31E-10
rs1569328	389.0255	6.47E-11	rs1569328	389.0255	6.47E-11	rs1569328	389.0255	6.47E-11
rs1646019	606.1476	8.62E-17	rs1646019	606.1476	8.62E-17	rs1646019	606.1476	8.62E-17
rs17129991	410.3462	2.81E-10	rs17129991	410.3462	2.81E-10	rs17129991	410.3462	2.81E-10
rs17293632	688.8775	3.70E-20	rs17293632	688.8775	3.70E-20	rs17293632	688.8775	3.70E-20
rs17388425	769.2465	6.54E-20	rs17388425	769.2465	6.54E-20	rs17388425	769.2465	6.54E-20
rs17391694	351.9615	2.62E-09	rs17391694	351.9615	2.62E-09	rs17391694	351.9615	2.62E-09
rs17622378	2062.584	7.17E-56	rs17622378	2062.584	7.17E-56	rs17622378	2062.584	7.17E-56
rs17694108	294.4707	3.29E-09	rs17694108	294.4707	3.29E-09	rs17694108	294.4707	3.29E-09
rs181826	536.628	4.53E-15	rs181826	536.628	4.53E-15	rs181826	536.628	4.53E-15
rs1847472	378.8982	1.09E-10	rs1847472	378.8982	1.09E-10	rs1847472	378.8982	1.09E-10
rs1927681	449.4354	2.42E-13	rs1927681	449.4354	2.42E-13	rs1927681	449.4354	2.42E-13
rs2024092	856.7346	7.13E-25	rs2024092	856.7346	7.13E-25	rs2024092	856.7346	7.13E-25
rs212388	579.723	1.80E-16	rs212388	579.723	1.80E-16	rs212388	579.723	1.80E-16
rs2153283	464.8112	2.39E-12	rs2153283	464.8112	2.39E-12	rs2153283	464.8112	2.39E-12
rs2227551	451.6324	4.72E-13	rs2227551	451.6324	4.72E-13	rs2227551	451.6324	4.72E-13
rs2270395	649.5767	8.93E-18	rs2270395	649.5767	8.93E-18	rs2270395	649.5767	8.93E-18
rs2284553	595.7384	5.63E-17	rs2284553	595.7384	5.63E-17	rs2284553	595.7384	5.63E-17
rs2395022	285.6961	3.13E-10	rs2395022	285.6961	3.13E-10	rs2395022	285.6961	3.13E-10
rs2413583	1423.934	7.72E-36	rs2413583	1423.934	7.72E-36	rs2413583	1423.934	7.72E-36
rs2538470	299.5613	1.05E-09	rs2538470	299.5613	1.05E-09	rs2538470	299.5613	1.05E-09
rs259964	291.5153	2.08E-09	rs259964	291.5153	2.08E-09	rs259964	291.5153	2.08E-09
rs2641348	326.5512	9.65E-10	rs2641348	326.5512	9.65E-10	rs2641348	326.5512	9.65E-10
rs26528	826.3572	1.29E-22	rs26528	826.3572	1.29E-22	rs26528	826.3572	1.29E-22
rs2847293	866.4858	6.14E-26	rs2847293	866.4858	6.14E-26	rs2847293	866.4858	6.14E-26
rs28999107	417.3493	1.29E-11	rs28999107	417.3493	1.29E-11	rs28999107	417.3493	1.29E-11
rs2974935	329.3259	5.80E-10	rs2974935	329.3259	5.80E-10	rs2974935	329.3259	5.80E-10
rs3024505	839.8039	3.95E-25	rs3024505	839.8039	3.95E-25	rs3024505	839.8039	3.95E-25
rs303429	322.3447	8.38E-10	rs303429	322.3447	8.38E-10	rs303429	322.3447	8.38E-10
rs3129871	400.5714	1.80E-11	rs3129871	400.5714	1.80E-11	rs3129871	400.5714	1.80E-11
rs3184504	263.8778	1.71E-08	rs3184504	263.8778	1.71E-08	rs3184504	263.8778	1.71E-08
rs3197999	1130.277	2.05E-33	rs3197999	1130.277	2.05E-33	rs3197999	1130.277	2.05E-33
rs34779708	955.9602	1.90E-27	rs34779708	955.9602	1.90E-27	rs34779708	955.9602	1.90E-27
rs34787213	624.9867	2.85E-16	rs34787213	624.9867	2.85E-16	rs34787213	624.9867	2.85E-16
rs34804116	483.3849	1.27E-13	rs34804116	483.3849	1.27E-13	rs34804116	483.3849	1.27E-13
rs35164067	773.1118	3.19E-20	rs35164067	773.1118	3.19E-20	rs35164067	773.1118	3.19E-20

rs35320439	349.1255	9.89E-10	rs35320439	349.1255	9.89E-10	rs35320439	349.1255	9.89E-10
rs35730213	1070.897	7.84E-28	rs35730213	1070.897	7.84E-28	rs35730213	1070.897	7.84E-28
rs36016881	396.5737	1.60E-10	rs36016881	396.5737	1.60E-10	rs36016881	396.5737	1.60E-10
rs3776414	427.5481	5.04E-13	rs3776414	427.5481	5.04E-13	rs3776414	427.5481	5.04E-13
rs3801810	456.9041	6.63E-14	rs3801810	456.9041	6.63E-14	rs3801810	456.9041	6.63E-14
rs438475	672.0539	3.42E-20	rs438475	672.0539	3.42E-20	rs438475	672.0539	3.42E-20
rs4703855	259.8926	3.03E-08	rs4703855	259.8926	3.03E-08	rs4703855	259.8926	3.03E-08
rs4795397	1013.206	3.84E-28	rs4795397	1013.206	3.84E-28	rs4795397	1013.206	3.84E-28
rs516246	756.6226	1.33E-20	rs516246	756.6226	1.33E-20	rs516246	756.6226	1.33E-20
rs559928	345.1139	3.75E-10	rs559928	345.1139	3.75E-10	rs559928	345.1139	3.75E-10
rs56163845	417.0559	9.40E-12	rs56163845	417.0559	9.40E-12	rs56163845	417.0559	9.40E-12
rs6062496	819.4354	3.82E-22	rs6062496	819.4354	3.82E-22	rs6062496	819.4354	3.82E-22
rs6074022	402.0877	2.70E-12	rs6074022	402.0877	2.70E-12	rs6074022	402.0877	2.70E-12
rs6111031	2508.531	9.61E-55	rs6111031	2508.531	9.61E-55	rs6111031	2508.531	9.61E-55
rs61839660	414.5495	3.19E-13	rs61839660	414.5495	3.19E-13	rs61839660	414.5495	3.19E-13
rs640466	310.4759	1.31E-09	rs640466	310.4759	1.31E-09	rs640466	310.4759	1.31E-09
rs6456426	568.7683	1.37E-16	rs6456426	568.7683	1.37E-16	rs6456426	568.7683	1.37E-16
rs6500315	855.5855	2.18E-23	rs6500315	855.5855	2.18E-23	rs6500315	855.5855	2.18E-23
rs6561151	871.4024	4.68E-25	rs6561151	871.4024	4.68E-25	rs6561151	871.4024	4.68E-25
rs6651252	581.0403	3.86E-16	rs6651252	581.0403	3.86E-16	rs6651252	581.0403	3.86E-16
rs6679677	698.7974	4.67E-17	rs6679677	698.7974	4.67E-17	rs6679677	698.7974	4.67E-17
rs6702421	490.1676	6.53E-15	rs6702421	490.1676	6.53E-15	rs6702421	490.1676	6.53E-15
rs6738394	345.5531	8.98E-11	rs6738394	345.5531	8.98E-11	rs6738394	345.5531	8.98E-11
rs6738490	3011.015	4.26E-78	rs6738490	3011.015	4.26E-78	rs6738490	3011.015	4.26E-78
rs6740462	444.604	1.74E-12	rs6740462	444.604	1.74E-12	rs6740462	444.604	1.74E-12
rs6827756	337.2986	3.27E-10	rs6827756	337.2986	3.27E-10	rs6827756	337.2986	3.27E-10
rs6908425	422.5766	4.81E-12	rs6908425	422.5766	4.81E-12	rs6908425	422.5766	4.81E-12
rs7015630	319.6113	9.00E-10	rs7015630	319.6113	9.00E-10	rs7015630	319.6113	9.00E-10
rs7085798	1773.754	1.53E-47	rs7085798	1773.754	1.53E-47	rs7085798	1773.754	1.53E-47
rs71624119	361.1734	6.57E-10	rs71624119	361.1734	6.57E-10	rs71624119	361.1734	6.57E-10
rs7194886	2991.268	1.42E-77	rs7194886	2991.268	1.42E-77	rs7194886	2991.268	1.42E-77
rs7236492	298.1178	9.09E-09	rs7236492	298.1178	9.09E-09	rs7236492	298.1178	9.09E-09
rs72727394	394.8176	5.28E-12	rs72727394	394.8176	5.28E-12	rs72727394	394.8176	5.28E-12
rs727563	316.5484	1.88E-10	rs727563	316.5484	1.88E-10	rs727563	316.5484	1.88E-10
rs7438704	372.882	3.42E-11	rs7438704	372.882	3.42E-11	rs7438704	372.882	3.42E-11
rs7517847	6754.072	1.38E-159	rs7517847	6754.072	1.38E-159	rs7517847	6754.072	1.38E-159
rs7608910	802.6889	2.95E-23	rs7608910	802.6889	2.95E-23	rs7608910	802.6889	2.95E-23
rs76906269	665.0524	1.75E-26	rs76906269	665.0524	1.75E-26	rs76906269	665.0524	1.75E-26
rs7773324	343.4652	1.06E-09	rs7773324	343.4652	1.06E-09	rs7773324	343.4652	1.06E-09
rs7786444	297.9321	9.83E-10	rs7786444	297.9321	9.83E-10	rs7786444	297.9321	9.83E-10
rs77981966	520.0566	2.19E-16	rs77981966	520.0566	2.19E-16	rs77981966	520.0566	2.19E-16
rs780094	751.0683	4.56E-22	rs780094	751.0683	4.56E-22	rs780094	751.0683	4.56E-22
rs7848647	1017.738	1.55E-27	rs7848647	1017.738	1.55E-27	rs7848647	1017.738	1.55E-27
rs7969592	308.2446	1.04E-09	rs7969592	308.2446	1.04E-09	rs7969592	308.2446	1.04E-09
rs915286	253.6819	2.59E-08	rs915286	253.6819	2.59E-08	rs915286	253.6819	2.59E-08
rs9264942	1207.646	6.78E-32	rs9264942	1207.646	6.78E-32	rs9264942	1207.646	6.78E-32
rs9457247	881.0207	2.08E-23	rs9457247	881.0207	2.08E-23	rs9457247	881.0207	2.08E-23
rs9491892	559.253	3.80E-17	rs9491892	559.253	3.80E-17	rs9491892	559.253	3.80E-17
rs9554587	363.6164	8.29E-11	rs9554587	363.6164	8.29E-11	rs9554587	363.6164	8.29E-11
rs9594766	311.0598	1.39E-09	rs9594766	311.0598	1.39E-09	rs9594766	311.0598	1.39E-09
rs9889296	941.4874	2.96E-25	rs9889296	941.4874	2.96E-25	rs9889296	941.4874	2.96E-25

Supplementary Table 7. SNPs for CD to ReA and PA identified from GWAS analysis

CD to ReA			CD to PA		
SNPs	*F*-statistics	genome-wide significance	SNPs	*F*-statistics	genome-wide significance
rs10758669	1185.637839	4.19E-34	rs10758669	1185.638	4.19E-34
rs10798069	285.7056681	4.25E-09	rs10798069	285.7057	4.25E-09
rs10800309	425.0198142	8.48E-13	rs10800309	425.0198	8.48E-13
rs10878302	376.5015663	4.20E-11	rs10878302	376.5016	4.20E-11
rs10956252	769.196064	8.34E-22	rs10956252	769.1961	8.34E-22
rs10995271	1961.281492	4.92E-53	rs10995271	1961.281	4.92E-53
rs11117431	819.503082	1.09E-19	rs11117431	819.5031	1.09E-19
rs11152949	903.5258418	2.18E-25	rs11152949	903.5258	2.18E-25
rs11159833	441.3087002	7.59E-14	rs11159833	441.3087	7.59E-14
rs11236797	1886.721889	8.54E-51	rs11236797	1886.722	8.54E-51
rs11691685	423.9052973	1.35E-11	rs11691685	423.9053	1.35E-11
rs11713774	497.6669108	1.09E-14	rs11713774	497.6669	1.09E-14
rs11793497	1625.530632	9.80E-44	rs11793497	1625.531	9.80E-44
rs1267501	269.1762782	9.69E-09	rs12411259	763.1321	1.43E-22
rs12694846	591.3454994	2.50E-17	rs1250573	1006.623	5.86E-26
rs12796489	3315.755971	4.96E-51	rs1267501	269.1763	9.69E-09
rs1292053	473.9218533	1.75E-14	rs12694846	591.3455	2.50E-17
rs1297258	917.8747624	2.11E-25	rs12796489	3315.756	4.96E-51
rs13407913	744.6965038	9.64E-22	rs1292053	473.9219	1.75E-14
rs1456896	472.4195616	1.03E-13	rs12949918	611.088	3.47E-17
rs1569328	389.0254536	6.47E-11	rs1297258	917.8748	2.11E-25
rs17129991	410.3461763	2.81E-10	rs13001325	818.3211	1.68E-22
rs17293632	688.8775149	3.70E-20	rs13407913	744.6965	9.64E-22
rs17622378	2062.583504	7.17E-56	rs1363907	593.4012	3.89E-16
rs17694108	294.4707036	3.29E-09	rs1456896	472.4196	1.03E-13
rs181826	536.6279644	4.53E-15	rs1517352	353.2318	1.31E-10
rs1927681	449.4353737	2.42E-13	rs1569328	389.0255	6.47E-11
rs2024092	856.7345506	7.13E-25	rs1646019	606.1476	8.62E-17
rs2153283	464.8112465	2.39E-12	rs17129991	410.3462	2.81E-10
rs2270395	649.5766881	8.93E-18	rs17293632	688.8775	3.70E-20
rs2395022	285.6960657	3.13E-10	rs17388425	769.2465	6.54E-20
rs2413583	1423.934229	7.72E-36	rs17391694	351.9615	2.62E-09
rs2538470	299.5613393	1.05E-09	rs17622378	2062.584	7.17E-56
rs259964	291.5153495	2.08E-09	rs17694108	294.4707	3.29E-09
rs2641348	326.5512492	9.65E-10	rs181826	536.628	4.53E-15
rs26528	826.3571713	1.29E-22	rs1847472	378.8982	1.09E-10
rs2974935	329.3258833	5.80E-10	rs1927681	449.4354	2.42E-13
rs3024505	839.8038661	3.95E-25	rs2024092	856.7346	7.13E-25
rs303429	322.3446591	8.38E-10	rs212388	579.723	1.80E-16
rs3197999	1130.27719	2.05E-33	rs2153283	464.8112	2.39E-12
rs34779708	955.9601909	1.90E-27	rs2227551	451.6324	4.72E-13
rs34787213	624.9867235	2.85E-16	rs2270395	649.5767	8.93E-18
rs34804116	483.3849071	1.27E-13	rs2284553	595.7384	5.63E-17
rs3776414	427.5480652	5.04E-13	rs2395022	285.6961	3.13E-10
rs3801810	456.9041325	6.63E-14	rs2413583	1423.934	7.72E-36
rs438475	672.0539199	3.42E-20	rs2538470	299.5613	1.05E-09
rs4703855	259.8925896	3.03E-08	rs259964	291.5153	2.08E-09
rs4795397	1013.205924	3.84E-28	rs2641348	326.5512	9.65E-10
rs56163845	417.0558661	9.40E-12	rs26528	826.3572	1.29E-22
rs6062496	819.4353717	3.82E-22	rs2847293	866.4858	6.14E-26
rs6074022	402.0877174	2.70E-12	rs28999107	417.3493	1.29E-11
rs6111031	2508.531295	9.61E-55	rs2974935	329.3259	5.80E-10
rs61839660	414.5495385	3.19E-13	rs3024505	839.8039	3.95E-25
rs640466	310.4759048	1.31E-09	rs303429	322.3447	8.38E-10
rs6456426	568.7682912	1.37E-16	rs3129871	400.5714	1.80E-11
rs6500315	855.5854698	2.18E-23	rs3184504	263.8778	1.71E-08
rs6561151	871.4023641	4.68E-25	rs3197999	1130.277	2.05E-33
rs6702421	490.1675666	6.53E-15	rs34779708	955.9602	1.90E-27
rs6738394	345.5531286	8.98E-11	rs34787213	624.9867	2.85E-16
rs6738490	3011.015123	4.26E-78	rs34804116	483.3849	1.27E-13
rs7015630	319.6113015	9.00E-10	rs35164067	773.1118	3.19E-20

rs7085798	1773.753654	1.53E-47	rs35320439	349.1255	9.89E-10
rs71624119	361.1734093	6.57E-10	rs35730213	1070.897	7.84E-28
rs7194886	2991.267564	1.42E-77	rs36016881	396.5737	1.60E-10
rs7236492	298.1178291	9.09E-09	rs3776414	427.5481	5.04E-13
rs72727394	394.8176132	5.28E-12	rs3801810	456.9041	6.63E-14
rs727563	316.5484276	1.88E-10	rs438475	672.0539	3.42E-20
rs7438704	372.881987	3.42E-11	rs4703855	259.8926	3.03E-08
rs76906269	665.0524081	1.75E-26	rs4795397	1013.206	3.84E-28
rs7786444	297.9320685	9.83E-10	rs516246	756.6226	1.33E-20
rs780094	751.0683151	4.56E-22	rs559928	345.1139	3.75E-10
rs7848647	1017.738437	1.55E-27	rs56163845	417.0559	9.40E-12
rs7969592	308.2445664	1.04E-09	rs6062496	819.4354	3.82E-22
rs915286	253.6818686	2.59E-08	rs6074022	402.0877	2.70E-12
rs9457247	881.0206637	2.08E-23	rs6111031	2508.531	9.61E-55
rs9491892	559.2530314	3.80E-17	rs61839660	414.5495	3.19E-13
rs9554587	363.61645	8.29E-11	rs640466	310.4759	1.31E-09
rs9594766	311.0597506	1.39E-09	rs6456426	568.7683	1.37E-16
rs9889296	941.4874291	2.96E-25	rs6500315	855.5855	2.18E-23
			rs6561151	871.4024	4.68E-25
			rs6651252	581.0403	3.86E-16
			rs6679677	698.7974	4.67E-17
			rs6702421	490.1676	6.53E-15
			rs6738394	345.5531	8.98E-11
			rs6738490	3011.015	4.26E-78
			rs6740462	444.604	1.74E-12
			rs6827756	337.2986	3.27E-10
			rs6908425	422.5766	4.81E-12
			rs7015630	319.6113	9.00E-10
			rs7085798	1773.754	1.53E-47
			rs71624119	361.1734	6.57E-10
			rs7194886	2991.268	1.42E-77
			rs7236492	298.1178	9.09E-09
			rs72727394	394.8176	5.28E-12
			rs727563	316.5484	1.88E-10
			rs7438704	372.882	3.42E-11
			rs7517847	6754.072	1.38E-159
			rs7608910	802.6889	2.95E-23
			rs76906269	665.0524	1.75E-26
			rs7773324	343.4652	1.06E-09
			rs7786444	297.9321	9.83E-10
			rs77981966	520.0566	2.19E-16
			rs780094	751.0683	4.56E-22
			rs7848647	1017.738	1.55E-27
			rs7969592	308.2446	1.04E-09
			rs915286	253.6819	2.59E-08
			rs9264942	1207.646	6.78E-32
			rs9457247	881.0207	2.08E-23
			rs9491892	559.253	3.80E-17
			rs9554587	363.6164	8.29E-11
			rs9594766	311.0598	1.39E-09
			rs9889296	941.4874	2.96E-25

Supplementary Table 8. SNPs for UC to RA, AS and PSA identified from GWAS analysis

UC to RA			UC to AS			UC to PSA		
SNPs	*F*-statistics	genome-wide significance	SNPs	*F*-statistics	genome-wide significance	SNPs	*F*-statistics	genome-wide significance
rs10185424	1.47E-14	536.0311644	rs10185424	536.0311644	1.47E-14	rs10185424	536.0311644	1.47E-14
rs10460566	1.60E-08	280.4717644	rs10460566	280.4717644	1.60E-08	rs10460566	280.4717644	1.60E-08
rs10748783	7.73E-39	1579.725723	rs10748783	1579.725723	7.73E-39	rs10748783	1579.725723	7.73E-39
rs10758669	1.04E-28	1083.507504	rs10758669	1083.507504	1.04E-28	rs10758669	1083.507504	1.04E-28
rs10761659	1.50E-20	792.2589013	rs10761659	792.2589013	1.50E-20	rs10761659	792.2589013	1.50E-20
rs1077773	5.96E-09	299.4390498	rs1077773	299.4390498	5.96E-09	rs1077773	299.4390498	5.96E-09
rs10870077	5.77E-27	1047.516559	rs10870077	1047.516559	5.77E-27	rs10870077	1047.516559	5.77E-27
rs10910092	1.42E-11	428.8183541	rs11083840	265.1375489	3.41E-08	rs10910092	428.8183541	1.42E-11
rs11083840	3.41E-08	265.1375489	rs11150589	366.9955254	3.28E-10	rs11083840	265.1375489	3.41E-08
rs11150589	3.28E-10	366.9955254	rs111830527	425.3387319	5.09E-11	rs11150589	366.9955254	3.28E-10
rs111830527	5.09E-11	425.3387319	rs11229555	294.4587362	1.21E-08	rs111830527	425.3387319	5.09E-11
rs11229555	1.21E-08	294.4587362	rs11230563	295.0170037	1.90E-08	rs11229555	294.4587362	1.21E-08
rs11230563	1.90E-08	295.0170037	rs11641184	350.5403988	4.24E-10	rs11230563	295.0170037	1.90E-08
rs11641184	4.24E-10	350.5403988	rs11676348	318.9689538	2.08E-09	rs11641184	350.5403988	4.24E-10
rs11676348	2.08E-09	318.9689538	rs1182188	561.279738	5.03E-15	rs11676348	318.9689538	2.08E-09
rs1182188	5.03E-15	561.279738	rs12318183	1451.938448	1.44E-37	rs1182188	561.279738	5.03E-15
rs12132349	3.64E-31	1310.401826	rs12718244	287.1262315	1.41E-08	rs12132349	1310.401826	3.64E-31
rs12318183	1.44E-37	1451.938448	rs12796489	2401.826763	1.22E-33	rs12318183	1451.938448	1.44E-37
rs12718244	1.41E-08	287.1262315	rs1297256	577.2668078	2.10E-15	rs12718244	287.1262315	1.41E-08
rs12720356	1.67E-11	425.3473632	rs13255292	289.7937554	3.82E-08	rs12720356	425.3473632	1.67E-11
rs12796489	1.22E-33	2401.826763	rs16841904	276.0034419	1.90E-08	rs12796489	2401.826763	1.22E-33
rs1297256	2.10E-15	577.2668078	rs17694108	427.5280488	6.17E-12	rs1297256	577.2668078	2.10E-15
rs13136827	2.35E-10	392.3526041	rs17780256	478.6996336	6.13E-13	rs13136827	392.3526041	2.35E-10
rs13255292	3.82E-08	289.7937554	rs1801274	1705.203046	1.43E-41	rs13255292	289.7937554	3.82E-08
rs13430791	1.39E-08	273.5340214	rs1927681	14670.26865	1.00E-200	rs13430791	273.5340214	1.39E-08
rs16841904	1.90E-08	276.0034419	rs2274351	289.959649	4.90E-08	rs16841904	276.0034419	1.90E-08
rs17694108	6.17E-12	427.5280488	rs2395022	307.9217522	2.88E-10	rs17694108	427.5280488	6.17E-12
rs17780256	6.13E-13	478.6996336	rs2516440	502.5432368	4.40E-13	rs17780256	478.6996336	6.13E-13
rs1801274	1.43E-41	1705.203046	rs272882	1087.188544	6.67E-26	rs1801274	1705.203046	1.43E-41
rs1927681	1.00E-200	14670.26865	rs2836883	2404.689557	1.47E-53	rs1927681	14670.26865	1.00E-200
rs1990760	1.78E-10	402.9030358	rs3024493	1582.737896	1.42E-43	rs1990760	402.9030358	1.78E-10
rs2274351	4.90E-08	289.959649	rs36070529	310.3975134	1.04E-08	rs2274351	289.959649	4.90E-08
rs2395022	2.88E-10	307.9217522	rs3776414	268.7482377	4.10E-08	rs2395022	307.9217522	2.88E-10
rs2497318	1.15E-08	291.4815089	rs4366152	730.2382511	7.79E-19	rs2497318	291.4815089	1.15E-08
rs2516440	4.40E-13	502.5432368	rs4656958	339.9816522	2.82E-09	rs2516440	502.5432368	4.40E-13
rs272882	6.67E-26	1087.188544	rs4676410	758.2362378	1.85E-19	rs272882	1087.188544	6.67E-26
rs2836883	1.47E-53	2404.689557	rs4728142	535.4352814	1.92E-14	rs2836883	2404.689557	1.47E-53
rs3024493	1.42E-43	1582.737896	rs4743820	316.2172337	4.05E-09	rs3024493	1582.737896	1.42E-43
rs34659678	5.95E-17	550.9614625	rs4747886	303.9846872	9.58E-09	rs34659678	550.9614625	5.95E-17
rs35223180	1.04E-15	676.7117494	rs4795397	1132.710699	1.01E-28	rs35223180	676.7117494	1.04E-15
rs36070529	1.04E-08	310.3975134	rs4812833	616.9497889	1.87E-16	rs36070529	310.3975134	1.04E-08
rs3774937	4.61E-14	500.3320946	rs483905	342.638878	3.16E-10	rs3774937	500.3320946	4.61E-14
rs3776414	4.10E-08	268.7482377	rs4947328	305.6542221	3.38E-10	rs3776414	268.7482377	4.10E-08
rs4366152	7.79E-19	730.2382511	rs4973341	278.389582	2.25E-08	rs4366152	730.2382511	7.79E-19
rs4656958	2.82E-09	339.9816522	rs4976646	322.0695341	2.52E-09	rs4656958	339.9816522	2.82E-09
rs4676410	1.85E-19	758.2362378	rs55808324	315.5611412	1.47E-09	rs4676410	758.2362378	1.85E-19
rs4712520	2.21E-08	296.7917155	rs59418206	284.2529611	1.45E-08	rs4712520	296.7917155	2.21E-08
rs4728142	1.92E-14	535.4352814	rs6062496	736.7327734	9.14E-19	rs4728142	535.4352814	1.92E-14
rs4743820	4.05E-09	316.2172337	rs6111031	2134.19894	1.33E-42	rs4743820	316.2172337	4.05E-09
rs4747886	9.58E-09	303.9846872	rs61893460	839.8313958	4.60E-22	rs4747886	303.9846872	9.58E-09
rs4795397	1.01E-28	1132.710699	rs6426833	3176.484188	3.77E-76	rs4795397	1132.710699	1.01E-28
rs4812833	1.87E-16	616.9497889	rs6466198	986.708138	1.90E-25	rs4812833	616.9497889	1.87E-16
rs483905	3.16E-10	342.638878	rs661054	811.7401409	3.18E-20	rs483905	342.638878	3.16E-10
rs4947328	3.38E-10	305.6542221	rs7240004	368.8491616	2.50E-10	rs4947328	305.6542221	3.38E-10
rs4973341	2.25E-08	278.389582	rs7404095	289.5784751	1.52E-08	rs4973341	278.389582	2.25E-08
rs4976646	2.52E-09	322.0695341	rs76546301	289.7544335	1.05E-10	rs4976646	322.0695341	2.52E-09
rs55808324	1.47E-09	315.5611412	rs76904798	298.2628091	2.78E-09	rs55808324	315.5611412	1.47E-09
rs56167332	7.27E-27	1037.708512	rs7738430	801.7825415	3.51E-27	rs56167332	1037.708512	7.27E-27
rs59418206	1.45E-08	284.2529611	rs79045992	298.0808335	1.43E-08	rs59418206	284.2529611	1.45E-08
rs6062496	9.14E-19	736.7327734	rs913678	292.4778966	1.23E-08	rs6062496	736.7327734	9.14E-19
rs6111031	1.33E-42	2134.19894	rs9271255	3786.928027	1.31E-94	rs6111031	2134.19894	1.33E-42

rs61893460	4.60E-22	839.8313958	rs941823	510.6281625	1.39E-13	rs61893460	839.8313958	4.60E-22
rs6426833	3.77E-76	3176.484188	rs9611131	592.9847186	3.84E-15	rs6426833	3176.484188	3.77E-76
rs6466198	1.90E-25	986.708138	rs9836291	1383.914227	8.20E-38	rs6466198	986.708138	1.90E-25
rs661054	3.18E-20	811.7401409	rs9941524	547.7129778	2.15E-14	rs661054	811.7401409	3.18E-20
rs6920220	4.78E-22	826.2062476				rs6920220	826.2062476	4.78E-22
rs7240004	2.50E-10	368.8491616				rs7240004	368.8491616	2.50E-10
rs7404095	1.52E-08	289.5784751				rs7404095	289.5784751	1.52E-08
rs7547569	8.71E-65	3634.183314				rs7547569	3634.183314	8.71E-65
rs7608910	1.25E-23	892.0866176				rs7608910	892.0866176	1.25E-23
rs76546301	1.05E-10	289.7544335				rs76546301	289.7544335	1.05E-10
rs76904798	2.78E-09	298.2628091				rs76904798	298.2628091	2.78E-09
rs7738430	3.51E-27	801.7825415				rs7738430	801.7825415	3.51E-27
rs79045992	1.43E-08	298.0808335				rs79045992	298.0808335	1.43E-08
rs8096327	2.24E-13	481.0130501				rs8096327	481.0130501	2.24E-13
rs913678	1.23E-08	292.4778966				rs913678	292.4778966	1.23E-08
rs9271255	1.31E-94	3786.928027				rs9271255	3786.928027	1.31E-94
rs941823	1.39E-13	510.6281625				rs941823	510.6281625	1.39E-13
rs9611131	3.84E-15	592.9847186				rs9611131	592.9847186	3.84E-15
rs9836291	8.20E-38	1383.914227				rs9836291	1383.914227	8.20E-38
rs9891119	1.72E-11	423.3722562				rs9891119	423.3722562	1.72E-11
rs9941524	2.15E-14	547.7129778				rs9941524	547.7129778	2.15E-14

Supplementary Table 9. SNPs for UC to OA and gout identified from GWAS analysis

UC to OA_Knee			UC to OA_Coxa			UC to gout		
SNPs	*F*-statistics	genome-wide significance	SNPs	*F*-statistics	genome-wide significance	SNPs	*F*-statistics	genome-wide significance
rs10185424	1.47E-14	536.0311644	rs10185424	1.47E-14	536.0311644	rs10185424	536.0311644	1.47E-14
rs10460566	1.60E-08	280.4717644	rs10460566	1.60E-08	280.4717644	rs10460566	280.4717644	1.60E-08
rs10748783	7.73E-39	1579.725723	rs10748783	7.73E-39	1579.725723	rs10748783	1579.725723	7.73E-39
rs10758669	1.04E-28	1083.507504	rs10758669	1.04E-28	1083.507504	rs10758669	1083.507504	1.04E-28
rs10761659	1.50E-20	792.2589013	rs10761659	1.50E-20	792.2589013	rs10761659	792.2589013	1.50E-20
rs1077773	5.96E-09	299.4390498	rs1077773	5.96E-09	299.4390498	rs1077773	299.4390498	5.96E-09
rs10870077	5.77E-27	1047.516559	rs10870077	5.77E-27	1047.516559	rs10870077	1047.516559	5.77E-27
rs10910092	1.42E-11	428.8183541	rs10910092	1.42E-11	428.8183541	rs10910092	428.8183541	1.42E-11
rs11083840	3.41E-08	265.1375489	rs11083840	3.41E-08	265.1375489	rs11150589	366.9955254	3.28E-10
rs11150589	3.28E-10	366.9955254	rs11150589	3.28E-10	366.9955254	rs111830527	425.3387319	5.09E-11
rs111830527	5.09E-11	425.3387319	rs111830527	5.09E-11	425.3387319	rs11229555	294.4587362	1.21E-08
rs11229555	1.21E-08	294.4587362	rs11229555	1.21E-08	294.4587362	rs11230563	295.0170037	1.90E-08
rs11230563	1.90E-08	295.0170037	rs11230563	1.90E-08	295.0170037	rs11641184	350.5403988	4.24E-10
rs11641184	4.24E-10	350.5403988	rs11641184	4.24E-10	350.5403988	rs11676348	318.9689538	2.08E-09
rs11676348	2.08E-09	318.9689538	rs11676348	2.08E-09	318.9689538	rs1182188	561.279738	5.03E-15
rs1182188	5.03E-15	561.279738	rs1182188	5.03E-15	561.279738	rs12132349	1310.401826	3.64E-31
rs12132349	3.64E-31	1310.401826	rs12132349	3.64E-31	1310.401826	rs12318183	1451.938448	1.44E-37
rs12318183	1.44E-37	1451.938448	rs12318183	1.44E-37	1451.938448	rs12718244	287.1262315	1.41E-08
rs12718244	1.41E-08	287.1262315	rs12718244	1.41E-08	287.1262315	rs12720356	425.3473632	1.67E-11
rs12720356	1.67E-11	425.3473632	rs12720356	1.67E-11	425.3473632	rs12796489	2401.826763	1.22E-33
rs12796489	1.22E-33	2401.826763	rs12796489	1.22E-33	2401.826763	rs13136827	392.3526041	2.35E-10
rs1297256	2.10E-15	577.2668078	rs1297256	2.10E-15	577.2668078	rs13255292	289.7937554	3.82E-08
rs13136827	2.35E-10	392.3526041	rs13136827	2.35E-10	392.3526041	rs13430791	273.5340214	1.39E-08
rs13255292	3.82E-08	289.7937554	rs13255292	3.82E-08	289.7937554	rs16841904	276.0034419	1.90E-08
rs13430791	1.39E-08	273.5340214	rs13430791	1.39E-08	273.5340214	rs17780256	478.6996336	6.13E-13
rs16841904	1.90E-08	276.0034419	rs16841904	1.90E-08	276.0034419	rs1801274	1705.203046	1.43E-41
rs17694108	6.17E-12	427.5280488	rs17694108	6.17E-12	427.5280488	rs1927681	14670.26865	1.00E-200
rs17780256	6.13E-13	478.6996336	rs17780256	6.13E-13	478.6996336	rs1990760	402.9030358	1.78E-10
rs1801274	1.43E-41	1705.203046	rs1801274	1.43E-41	1705.203046	rs2274351	289.959649	4.90E-08
rs1927681	1.00E-200	14670.26865	rs1927681	1.00E-200	14670.26865	rs2395022	307.9217522	2.88E-10
rs1990760	1.78E-10	402.9030358	rs1990760	1.78E-10	402.9030358	rs2497318	291.4815089	1.15E-08
rs2274351	4.90E-08	289.959649	rs2274351	4.90E-08	289.959649	rs2516440	502.5432368	4.40E-13
rs2395022	2.88E-10	307.9217522	rs2395022	2.88E-10	307.9217522	rs272882	1087.188544	6.67E-26
rs2497318	1.15E-08	291.4815089	rs2497318	1.15E-08	291.4815089	rs3024493	1582.737896	1.42E-43
rs2516440	4.40E-13	502.5432368	rs2516440	4.40E-13	502.5432368	rs34659678	550.9614625	5.95E-17
rs272882	6.67E-26	1087.188544	rs272882	6.67E-26	1087.188544	rs35223180	676.7117494	1.04E-15
rs2836883	1.47E-53	2404.689557	rs2836883	1.47E-53	2404.689557	rs36070529	310.3975134	1.04E-08
rs3024493	1.42E-43	1582.737896	rs3024493	1.42E-43	1582.737896	rs3774937	500.3320946	4.61E-14

rs34659678	5.95E-17	550.9614625	rs34659678	5.95E-17	550.9614625	rs3776414	268.7482377	4.10E-08
rs35223180	1.04E-15	676.7117494	rs35223180	1.04E-15	676.7117494	rs4366152	730.2382511	7.79E-19
rs36070529	1.04E-08	310.3975134	rs36070529	1.04E-08	310.3975134	rs4656958	339.9816522	2.82E-09
rs3774937	4.61E-14	500.3320946	rs3774937	4.61E-14	500.3320946	rs4676410	758.2362378	1.85E-19
rs3776414	4.10E-08	268.7482377	rs3776414	4.10E-08	268.7482377	rs4712520	296.7917155	2.21E-08
rs4366152	7.79E-19	730.2382511	rs4366152	7.79E-19	730.2382511	rs4728142	535.4352814	1.92E-14
rs4656958	2.82E-09	339.9816522	rs4656958	2.82E-09	339.9816522	rs4743820	316.2172337	4.05E-09
rs4676410	1.85E-19	758.2362378	rs4676410	1.85E-19	758.2362378	rs4747886	303.9846872	9.58E-09
rs4712520	2.21E-08	296.7917155	rs4712520	2.21E-08	296.7917155	rs4795397	1132.710699	1.01E-28
rs4728142	1.92E-14	535.4352814	rs4728142	1.92E-14	535.4352814	rs483905	342.638878	3.16E-10
rs4743820	4.05E-09	316.2172337	rs4743820	4.05E-09	316.2172337	rs4947328	305.6542221	3.38E-10
rs4747886	9.58E-09	303.9846872	rs4747886	9.58E-09	303.9846872	rs4973341	278.389582	2.25E-08
rs4795397	1.01E-28	1132.710699	rs4795397	1.01E-28	1132.710699	rs4976646	322.0695341	2.52E-09
rs4812833	1.87E-16	616.9497889	rs4812833	1.87E-16	616.9497889	rs55808324	315.5611412	1.47E-09
rs483905	3.16E-10	342.638878	rs483905	3.16E-10	342.638878	rs56167332	1037.708512	7.27E-27
rs4947328	3.38E-10	305.6542221	rs4947328	3.38E-10	305.6542221	rs59418206	284.2529611	1.45E-08
rs4973341	2.25E-08	278.389582	rs4973341	2.25E-08	278.389582	rs61893460	839.8313958	4.60E-22
rs4976646	2.52E-09	322.0695341	rs4976646	2.52E-09	322.0695341	rs6426833	3176.484188	3.77E-76
rs55808324	1.47E-09	315.5611412	rs55808324	1.47E-09	315.5611412	rs6466198	986.708138	1.90E-25
rs56167332	7.27E-27	1037.708512	rs56167332	7.27E-27	1037.708512	rs661054	811.7401409	3.18E-20
rs59418206	1.45E-08	284.2529611	rs59418206	1.45E-08	284.2529611	rs6920220	826.2062476	4.78E-22
rs6062496	9.14E-19	736.7327734	rs6062496	9.14E-19	736.7327734	rs7240004	368.8491616	2.50E-10
rs6111031	1.33E-42	2134.19894	rs6111031	1.33E-42	2134.19894	rs7404095	289.5784751	1.52E-08
rs61893460	4.60E-22	839.8313958	rs61893460	4.60E-22	839.8313958	rs7547569	3634.183314	8.71E-65
rs6426833	3.77E-76	3176.484188	rs6426833	3.77E-76	3176.484188	rs7608910	892.0866176	1.25E-23
rs6466198	1.90E-25	986.708138	rs6466198	1.90E-25	986.708138	rs76546301	289.7544335	1.05E-10
rs661054	3.18E-20	811.7401409	rs661054	3.18E-20	811.7401409	rs76904798	298.2628091	2.78E-09
rs6920220	4.78E-22	826.2062476	rs6920220	4.78E-22	826.2062476	rs7738430	801.7825415	3.51E-27
rs7240004	2.50E-10	368.8491616	rs7240004	2.50E-10	368.8491616	rs79045992	298.0808335	1.43E-08
rs7404095	1.52E-08	289.5784751	rs7404095	1.52E-08	289.5784751	rs8096327	481.0130501	2.24E-13
rs7547569	8.71E-65	3634.183314	rs7547569	8.71E-65	3634.183314	rs9271255	3786.928027	1.31E-94
rs7608910	1.25E-23	892.0866176	rs7608910	1.25E-23	892.0866176	rs941823	510.6281625	1.39E-13
rs76546301	1.05E-10	289.7544335	rs76546301	1.05E-10	289.7544335	rs9836291	1383.914227	8.20E-38
rs76904798	2.78E-09	298.2628091	rs76904798	2.78E-09	298.2628091	rs9891119	423.3722562	1.72E-11
rs7738430	3.51E-27	801.7825415	rs7738430	3.51E-27	801.7825415	rs9941524	547.7129778	2.15E-14
rs79045992	1.43E-08	298.0808335	rs79045992	1.43E-08	298.0808335			
rs8096327	2.24E-13	481.0130501	rs8096327	2.24E-13	481.0130501			
rs913678	1.23E-08	292.4778966	rs913678	1.23E-08	292.4778966			
rs9271255	1.31E-94	3786.928027	rs9271255	1.31E-94	3786.928027			
rs941823	1.39E-13	510.6281625	rs941823	1.39E-13	510.6281625			
rs9611131	3.84E-15	592.9847186	rs9611131	3.84E-15	592.9847186			
rs9836291	8.20E-38	1383.914227	rs9836291	8.20E-38	1383.914227			
rs9891119	1.72E-11	423.3722562	rs9891119	1.72E-11	423.3722562			
rs9941524	2.15E-14	547.7129778	rs9941524	2.15E-14	547.7129778			

Supplementary Table 10. SNPs for UC to ReA and PA identified from GWAS analysis

UC to ReA			UC to PA		
SNPs	*F*-statistics	genome-wide significance	SNPs	*F*-statistics	genome-wide significance
rs10185424	536.0311644	1.47E-14	rs10185424	536.0311644	1.47E-14
rs10460566	280.4717644	1.60E-08	rs10460566	280.4717644	1.60E-08
rs10748783	1579.725723	7.73E-39	rs10748783	1579.725723	7.73E-39
rs10758669	1083.507504	1.04E-28	rs10758669	1083.507504	1.04E-28
rs10761659	792.2589013	1.50E-20	rs10761659	792.2589013	1.50E-20
rs1077773	299.4390498	5.96E-09	rs1077773	299.4390498	5.96E-09
rs10870077	1047.516559	5.77E-27	rs10870077	1047.516559	5.77E-27
rs11083840	265.1375489	3.41E-08	rs10910092	428.8183541	1.42E-11
rs11150589	366.9955254	3.28E-10	rs11083840	265.1375489	3.41E-08
rs111830527	425.3387319	5.09E-11	rs11150589	366.9955254	3.28E-10
rs11229555	294.4587362	1.21E-08	rs111830527	425.3387319	5.09E-11
rs11230563	295.0170037	1.90E-08	rs11229555	294.4587362	1.21E-08
rs11641184	350.5403988	4.24E-10	rs11230563	295.0170037	1.90E-08
rs11676348	318.9689538	2.08E-09	rs11641184	350.5403988	4.24E-10
rs1182188	561.279738	5.03E-15	rs11676348	318.9689538	2.08E-09
rs12318183	1451.938448	1.44E-37	rs1182188	561.279738	5.03E-15

rs12718244	287.1262315	1.41E-08	rs12132349	1310.401826	3.64E-31
rs12796489	2401.826763	1.22E-33	rs12318183	1451.938448	1.44E-37
rs1297256	577.2668078	2.10E-15	rs12718244	287.1262315	1.41E-08
rs13255292	289.7937554	3.82E-08	rs12720356	425.3473632	1.67E-11
rs16841904	276.0034419	1.90E-08	rs12796489	2401.826763	1.22E-33
rs17694108	427.5280488	6.17E-12	rs1297256	577.2668078	2.10E-15
rs17780256	478.6996336	6.13E-13	rs13136827	392.3526041	2.35E-10
rs1801274	1705.203046	1.43E-41	rs13255292	289.7937554	3.82E-08
rs1927681	14670.26865	1.00E-200	rs13430791	273.5340214	1.39E-08
rs2274351	289.959649	4.90E-08	rs16841904	276.0034419	1.90E-08
rs2395022	307.9217522	2.88E-10	rs17694108	427.5280488	6.17E-12
rs2516440	502.5432368	4.40E-13	rs17780256	478.6996336	6.13E-13
rs272882	1087.188544	6.67E-26	rs1801274	1705.203046	1.43E-41
rs2836883	2404.689557	1.47E-53	rs1927681	14670.26865	1.00E-200
rs3024493	1582.737896	1.42E-43	rs1990760	402.9030358	1.78E-10
rs36070529	310.3975134	1.04E-08	rs2274351	289.959649	4.90E-08
rs3776414	268.7482377	4.10E-08	rs2395022	307.9217522	2.88E-10
rs4366152	730.2382511	7.79E-19	rs2497318	291.4815089	1.15E-08
rs4656958	339.9816522	2.82E-09	rs2516440	502.5432368	4.40E-13
rs4676410	758.2362378	1.85E-19	rs272882	1087.188544	6.67E-26
rs4728142	535.4352814	1.92E-14	rs2836883	2404.689557	1.47E-53
rs4743820	316.2172337	4.05E-09	rs3024493	1582.737896	1.42E-43
rs4747886	303.9846872	9.58E-09	rs34659678	550.9614625	5.95E-17
rs4795397	1132.710699	1.01E-28	rs35223180	676.7117494	1.04E-15
rs4812833	616.9497889	1.87E-16	rs36070529	310.3975134	1.04E-08
rs483905	342.638878	3.16E-10	rs3774937	500.3320946	4.61E-14
rs4947328	305.6542221	3.38E-10	rs3776414	268.7482377	4.10E-08
rs4973341	278.389582	2.25E-08	rs4366152	730.2382511	7.79E-19
rs4976646	322.0695341	2.52E-09	rs4656958	339.9816522	2.82E-09
rs55808324	315.5611412	1.47E-09	rs4676410	758.2362378	1.85E-19
rs59418206	284.2529611	1.45E-08	rs4712520	296.7917155	2.21E-08
rs6062496	736.7327734	9.14E-19	rs4728142	535.4352814	1.92E-14
rs6111031	2134.19894	1.33E-42	rs4743820	316.2172337	4.05E-09
rs61893460	839.8313958	4.60E-22	rs4747886	303.9846872	9.58E-09
rs6426833	3176.484188	3.77E-76	rs4795397	1132.710699	1.01E-28
rs6466198	986.708138	1.90E-25	rs4812833	616.9497889	1.87E-16
rs661054	811.7401409	3.18E-20	rs483905	342.638878	3.16E-10
rs7240004	368.8491616	2.50E-10	rs4947328	305.6542221	3.38E-10
rs7404095	289.5784751	1.52E-08	rs4973341	278.389582	2.25E-08
rs76546301	289.7544335	1.05E-10	rs4976646	322.0695341	2.52E-09
rs76904798	298.2628091	2.78E-09	rs55808324	315.5611412	1.47E-09
rs7738430	801.7825415	3.51E-27	rs56167332	1037.708512	7.27E-27
rs79045992	298.0808335	1.43E-08	rs59418206	284.2529611	1.45E-08
rs913678	292.4778966	1.23E-08	rs6062496	736.7327734	9.14E-19
rs9271255	3786.928027	1.31E-94	rs6111031	2134.19894	1.33E-42
rs941823	510.6281625	1.39E-13	rs61893460	839.8313958	4.60E-22
rs9611131	592.9847186	3.84E-15	rs6426833	3176.484188	3.77E-76
rs9836291	1383.914227	8.20E-38	rs6466198	986.708138	1.90E-25
rs9941524	547.7129778	2.15E-14	rs661054	811.7401409	3.18E-20
			rs6920220	826.2062476	4.78E-22
			rs7240004	368.8491616	2.50E-10
			rs7404095	289.5784751	1.52E-08
			rs7547569	3634.183314	8.71E-65
			rs7608910	892.0866176	1.25E-23
			rs76546301	289.7544335	1.05E-10
			rs76904798	298.2628091	2.78E-09
			rs7738430	801.7825415	3.51E-27
			rs79045992	298.0808335	1.43E-08
			rs8096327	481.0130501	2.24E-13
			rs913678	292.4778966	1.23E-08
			rs9271255	3786.928027	1.31E-94
			rs941823	510.6281625	1.39E-13
			rs9611131	592.9847186	3.84E-15
			rs9836291	1383.914227	8.20E-38
			rs9891119	423.3722562	1.72E-11
			rs9941524	547.7129778	2.15E-14

Supplementary Table 11. MR analysis for IBD to arthritis

Exposure	Outcome	Methods	SNP	beta	se	pval	lo_ci	up_ci	or	or_ci	or_hi	Pleiotropy (MR-Egger)	heterogeneity
IBD	RA	Inverse variance weighted	125	0.041677	0.032052	0.193498	-0.02114	0.1045	1.042558	0.979077	1.110155		
		Weighted median	125	0.037492	0.022945	0.102262	-0.00748	0.082464	1.038203	0.992547	1.085959		
		MR Egger	125	-0.07093	0.076744	0.357173	-0.22135	0.079489	0.931527	0.801438	1.082733		
		Penalised weighted median	125	0.039797	0.023757	0.093901	-0.00677	0.08636	1.040599	0.993256	1.090199		
		Weighted mode	125	0.008553	0.032694	0.794055	-0.05553	0.072633	1.00859	0.945986	1.075336		
		Inverse variance weighted (multiplicative random effects)	125	0.041677	0.032052	0.193498	-0.02114	0.1045	1.042558	0.979077	1.110155		
	AS	Inverse variance weighted	94	0.19406	0.044469	1.28E-05	0.1069	0.28122	1.214169	1.112823	1.324745	0.540494	7.57E-12
		Weighted median	94	0.256109	0.048923	1.65E-07	0.160221	0.351997	1.291893	1.17377	1.421905		
		MR Egger	94	0.126066	0.119331	0.293535	-0.10782	0.359955	1.134357	0.897786	1.433264		
		Penalised weighted median	94	0.281076	0.050078	1.99E-08	0.182924	0.379229	1.324555	1.200723	1.461157		
		Weighted mode	94	0.307378	0.101606	0.003213	0.10823	0.506526	1.359855	1.114304	1.659516		
		Inverse variance weighted (multiplicative random effects)	94	0.19406	0.044469	1.28E-05	0.1069	0.28122	1.214169	1.112823	1.324745		
	PSA	Inverse variance weighted	107	0.166802	0.061321	0.006525	0.046613	0.28699	1.18152	1.047717	1.332411	0.553241	7.11E-77
		Weighted median	107	0.142092	0.040873	0.000508	0.061982	0.222202	1.152683	1.063943	1.248824		
		MR Egger	107	0.087181	0.147311	0.555243	-0.20155	0.375911	1.091095	0.817465	1.456317		
		Penalised weighted median	107	0.146603	0.039573	0.000212	0.069039	0.224167	1.157894	1.071478	1.25128		
		Weighted mode	107	0.195415	0.068223	0.005039	0.061697	0.329132	1.215815	1.06364	1.389762		
		Inverse variance weighted (multiplicative random effects)	107	0.166802	0.061321	0.006525	0.046613	0.28699	1.18152	1.047717	1.332411		
	OA_Knee	Inverse variance weighted	125	0.003537	0.010186	0.728374	-0.01643	0.023501	1.003544	0.983708	1.02378		
		Weighted median	125	0.002228	0.013037	0.864335	-0.02333	0.02778	1.00223	0.976944	1.02817		
		MR Egger	125	-0.03118	0.024424	0.204141	-0.07905	0.016691	0.969301	0.923993	1.016831		
		Penalised weighted median	125	0.002259	0.012518	0.856797	-0.02228	0.026793	1.002261	0.977971	1.027155		
		Weighted mode	125	-0.00137	0.01783	0.938854	-0.03632	0.033576	0.99863	0.964334	1.034146		
		Inverse variance weighted (multiplicative random effects)	125	0.003537	0.010186	0.728374	-0.01643	0.023501	1.003544	0.983708	1.02378		
	OA_Coxa	Inverse variance weighted	125	-0.00776	0.013105	0.553709	-0.03345	0.017925	0.992269	0.967106	1.018087		
		Weighted median	125	-0.00421	0.016508	0.798895	-0.03656	0.028149	0.995803	0.9641	1.028549		
		MR Egger	125	0.001687	0.03176	0.957723	-0.06056	0.063936	1.001688	0.941235	1.066024		

		Penalised weighted median	125	-0.0042	0.017012	0.804856	-0.03755	0.02914	0.995806	0.96315	1.029569		
		Weighted mode	125	0.005849	0.024426	0.811158	-0.04203	0.053723	1.005866	0.958845	1.055193		
		Inverse variance weighted (multiplicative random effects)	125	-0.00776	0.013105	0.553709	-0.03345	0.017925	0.992269	0.967106	1.018087		
	Gout	Inverse variance weighted	125	0.035052	0.022318	0.116288	-0.00869	0.078796	1.035674	0.991346	1.081983		
		Weighted median	125	0.047976	0.023899	0.0447	0.001134	0.094818	1.049145	1.001135	1.099458		
		MR Egger	125	0.06191	0.053982	0.253662	-0.04389	0.167714	1.063866	0.957055	1.182598		
		Penalised weighted median	125	0.06283	0.023291	0.006983	0.01718	0.108479	1.064845	1.017328	1.114582		
		Weighted mode	125	0.090287	0.036192	0.013923	0.01935	0.161224	1.094489	1.019539	1.174948		
		Inverse variance weighted (multiplicative random effects)	125	0.035052	0.022318	0.116288	-0.00869	0.078796	1.035674	0.991346	1.081983		
	ReA	Inverse variance weighted	91	0.101321	0.03403	0.002907	0.034622	0.16802	1.106632	1.035229	1.18296	0.79846	0.019522
		Weighted median	91	0.094579	0.045908	0.03938	0.0046	0.184559	1.099196	1.00461	1.202687		
		MR Egger	91	0.079454	0.09198	0.390008	-0.10083	0.259735	1.082696	0.90409	1.296586		
		Penalised weighted median	91	0.094766	0.04626	0.040507	0.004096	0.185436	1.099401	1.004104	1.203743		
		Weighted mode	91	0.090696	0.067927	0.185176	-0.04244	0.223833	1.094936	0.958448	1.250862		
		Inverse variance weighted (multiplicative random effects)	91	0.101321	0.03403	0.002907	0.034622	0.16802	1.106632	1.035229	1.18296		
	PA	Inverse variance weighted	125	0.024543	0.030712	0.424212	-0.03565	0.08474	1.024847	0.964975	1.088434		
		Weighted median	125	0.028505	0.044655	0.523252	-0.05902	0.11603	1.028916	0.942689	1.12303		
		MR Egger	125	0.068109	0.074269	0.360909	-0.07746	0.213676	1.070482	0.925465	1.238222		
		Penalised weighted median	125	0.026942	0.045034	0.549664	-0.06132	0.115208	1.027308	0.940518	1.122107		
		Weighted mode	125	0.077619	0.089781	0.388964	-0.09835	0.253591	1.080711	0.906329	1.288644		
		Inverse variance weighted (multiplicative random effects)	125	0.024543	0.030712	0.424212	-0.03565	0.08474	1.024847	0.964975	1.088434		

Supplementary Table 12. MR analysis for CD to arthritis

Exposure	Outcome	Methods	SNP	beta	se	pval	lo_ci	up_ci	or	or_ci	or_hi	pleiotropy (MR-Egger)	heterogeneity
CD	RA	Inverse variance weighted	111	0.013136	0.043107	0.760575	-0.07135	0.097625	1.013222	0.931133	1.102549		
		Weighted median	111	-0.00501	0.020296	0.805033	-0.04479	0.034771	0.995003	0.956198	1.035382		
		MR Egger	111	-0.009	0.12046	0.940597	-0.2451	0.227104	0.991043	0.782627	1.25496		
		Penalised weighted median	111	-0.02469	0.020479	0.227946	-0.06483	0.015448	0.975612	0.937228	1.015568		
		Weighted mode	111	-0.0148	0.026093	0.571747	-0.06594	0.036343	0.985309	0.936185	1.037012		
		Inverse variance weighted (multiplicative random effects)	111	0.013136	0.043107	0.760575	-0.07135	0.097625	1.013222	0.931133	1.102549		
	AS	Inverse variance weighted	77	0.152296	0.042686	0.00036	0.068632	0.23596	1.164505	1.071042	1.266123	0.809403	1.42E-12
		Weighted median	77	0.172875	0.046655	0.000211	0.081431	0.264319	1.188718	1.084839	1.302543		
		MR Egger	77	0.124225	0.12367	0.318374	-0.11817	0.366619	1.132271	0.888546	1.442848		
		Penalised weighted median	77	0.198495	0.043665	5.47E-06	0.112912	0.284078	1.219566	1.119533	1.328537		
		Weighted mode	77	0.16539	0.085751	0.0575	-0.00268	0.333462	1.179853	0.997321	1.395792		
		Inverse variance weighted (multiplicative random effects)	77	0.152296	0.042686	0.00036	0.068632	0.23596	1.164505	1.071042	1.266123		
	PSA	Inverse variance weighted	92	0.135625	0.056556	0.016482	0.024775	0.246475	1.145252	1.025084	1.279507	0.101462	1.74E-71
		Weighted median	92	0.056989	0.038738	0.14125	-0.01894	0.132916	1.058645	0.981241	1.142154		
		MR Egger	92	-0.09762	0.151682	0.521477	-0.39492	0.199676	0.906992	0.673735	1.221007		
		Penalised weighted median	92	0.059877	0.039493	0.129479	-0.01753	0.137283	1.061706	0.982624	1.147152		
		Weighted mode	92	-0.07887	0.052293	0.134962	-0.18136	0.023625	0.924161	0.834133	1.023906		
		Inverse variance weighted (multiplicative random effects)	92	0.135625	0.056556	0.016482	0.024775	0.246475	1.145252	1.025084	1.279507		
	OA_Knee	Inverse variance weighted	111	-0.01218	0.009349	0.19271	-0.0305	0.006146	0.987896	0.969959	1.006165		
		Weighted median	111	-0.02013	0.010888	0.064501	-0.04147	0.001212	0.980072	0.959378	1.001213		
		MR Egger	111	-0.06074	0.025626	0.019525	-0.11097	-0.01052	0.941064	0.894965	0.989538		
		Penalised weighted median	111	-0.02122	0.011378	0.062244	-0.04352	0.001086	0.979008	0.957416	1.001087		
		Weighted mode	111	-0.04027	0.019326	0.039504	-0.07815	-0.00239	0.96053	0.924827	0.997612		
		Inverse variance weighted (multiplicative random effects)	111	-0.01218	0.009349	0.19271	-0.0305	0.006146	0.987896	0.969959	1.006165		
	OA_Coxa	Inverse variance weighted	111	-0.01498	0.011196	0.180878	-0.03693	0.006963	0.98513	0.963747	1.006988		
		Weighted median	111	-0.02431	0.014657	0.097142	-0.05304	0.004414	0.97598	0.948341	1.004423		
		MR Egger	111	-0.03386	0.031203	0.280193	-0.09502	0.027294	0.966703	0.909354	1.02767		
		Penalised	111	-	0.01442	0.09350	-	0.00407	0.97609	0.94888	1.00408		

		weighted median		0.02419	4	3	0.05246	9	8	9	7		
		Weighted mode	111	-0.02445	0.019264	0.207091	-0.06221	0.01331	0.975848	0.939689	1.013399		
		Inverse variance weighted (multiplicative random effects)	111	-0.01498	0.011196	0.180878	-0.03693	0.006963	0.98513	0.963747	1.006988		
	Gout	Inverse variance weighted	111	0.030559	0.021121	0.147945	-0.01084	0.071957	1.031031	0.98922	1.074609		
		Weighted median	111	0.016096	0.022362	0.471632	-0.02773	0.059925	1.016227	0.972649	1.061757		
		MR Egger	111	-0.00777	0.058854	0.895173	-0.12313	0.107581	0.992257	0.884152	1.113581		
		Penalised weighted median	111	0.011124	0.021353	0.6024	-0.03073	0.052977	1.011186	0.969739	1.054405		
		Weighted mode	111	-0.01673	0.039729	0.674447	-0.0946	0.061136	0.983406	0.909735	1.063044		
		Inverse variance weighted (multiplicative random effects)	111	0.030559	0.021121	0.147945	-0.01084	0.071957	1.031031	0.98922	1.074609		
	ReA	Inverse variance weighted	78	0.065579	0.032785	0.045466	0.001322	0.129837	1.067777	1.001323	1.138643	0.402088	0.002837
		Weighted median	78	0.065602	0.040999	0.109575	-0.01476	0.145959	1.067802	0.985353	1.157149		
		MR Egger	78	-0.01026	0.095809	0.915013	-0.19805	0.177528	0.989794	0.820333	1.194261		
		Penalised weighted median	78	0.077622	0.042041	0.064842	-0.00478	0.160021	1.080714	0.995233	1.173536		
		Weighted mode	78	0.084127	0.072171	0.247353	-0.05733	0.225582	1.087767	0.944283	1.253052		
		Inverse variance weighted (multiplicative random effects)	78	0.065579	0.032785	0.045466	0.001322	0.129837	1.067777	1.001323	1.138643		
	PA	Inverse variance weighted	111	0.007895	0.029508	0.78905	-0.04994	0.065731	1.007926	0.951285	1.067939		
		Weighted median	111	0.003998	0.042318	0.924727	-0.07894	0.086941	1.004006	0.924091	1.090833		
		MR Egger	111	0.062719	0.08229	0.447603	-0.09857	0.224007	1.064728	0.906133	1.251079		
		Penalised weighted median	111	-0.00384	0.040134	0.923829	-0.0825	0.074825	0.99617	0.920812	1.077695		
		Weighted mode	111	-0.15669	0.092247	0.092228	-0.33749	0.024117	0.854971	0.713557	1.02441		
		Inverse variance weighted (multiplicative random effects)	111	0.007895	0.029508	0.78905	-0.04994	0.065731	1.007926	0.951285	1.067939		

Supplementary Table 13. MR analysis for UC to arthritis

Exposure	Outcome	Methods	SNP	beta	se	pval	lo_ci	up_ci	or	or_ci	or_hi	pleiotropy (MR-Egger)	heterogeneity
UC	RA	Inverse variance weighted	80	0.075982	0.030213	0.011907	0.016765	0.1352	1.078944	1.016906	1.144765		
		Weighted median	80	-0.0115	0.023653	0.62693	-0.05786	0.034863	0.988569	0.943786	1.035478		
		MR Egger	80	0.016206	0.072016	0.822537	-0.12494	0.157357	1.016338	0.882546	1.170413		
		Penalised weighted median	80	-0.01672	0.023266	0.472422	-0.06232	0.028884	0.983421	0.939582	1.029305		
		Weighted mode	80	-0.00813	0.027961	0.772018	-0.06293	0.046675	0.991904	0.939006	1.047782		
		Inverse variance weighted (multiplicative random effects)	80	0.075982	0.030213	0.011907	0.016765	0.1352	1.078944	1.016906	1.144765		
	AS	Inverse variance weighted	63	0.156013	0.081678	0.056119	-0.00407	0.316101	1.168842	0.995934	1.371769		
		Weighted median	63	0.10387	0.05362	0.052725	-0.00122	0.208965	1.109456	0.998776	1.232402		
		MR Egger	63	-0.00549	0.197298	0.977891	-0.39219	0.381214	0.994525	0.675573	1.464061		
		Penalised weighted median	63	0.1191	0.056634	0.035467	0.008098	0.230103	1.126483	1.008131	1.258729		
		Weighted mode	63	0.217844	0.098231	0.030252	0.02531	0.410377	1.243393	1.025633	1.507386		
		Inverse variance weighted (multiplicative random effects)	63	0.156013	0.081678	0.056119	-0.00407	0.316101	1.168842	0.995934	1.371769		
	PSA	Inverse variance weighted	80	0.049038	0.048481	0.311782	-0.04598	0.144061	1.05026	0.955057	1.154954		
		Weighted median	80	0.060842	0.044709	0.173564	-0.02679	0.148473	1.062731	0.973568	1.160061		
		MR Egger	80	0.03416	0.117021	0.77113	-0.1952	0.263521	1.03475	0.822669	1.301504		
		Penalised weighted median	80	0.071438	0.044031	0.104702	-0.01486	0.157738	1.074052	0.985248	1.17086		
		Weighted mode	80	0.071061	0.0991	0.475449	-0.12317	0.265297	1.073647	0.884109	1.303818		
		Inverse variance weighted (multiplicative random effects)	80	0.049038	0.048481	0.311782	-0.04598	0.144061	1.05026	0.955057	1.154954		
	OA_Knee	Inverse variance weighted	80	0.014215	0.00998	0.154333	-0.00535	0.033776	1.014317	0.994669	1.034353		
		Weighted median	80	0.026745	0.012466	0.031923	0.002311	0.051178	1.027106	1.002314	1.052511		
		MR Egger	80	-0.00581	0.023822	0.8079	-0.0525	0.04088	0.994205	0.948851	1.041727		
		Penalised weighted median	80	0.03171	0.012081	0.008668	0.008032	0.055388	1.032218	1.008064	1.056951		
		Weighted mode	80	0.035368	0.021023	0.09646	-0.00584	0.076574	1.036001	0.994179	1.079582		
		Inverse variance weighted (multiplicative random effects)	80	0.014215	0.00998	0.154333	-0.00535	0.033776	1.014317	0.994669	1.034353		
	OA_Coxa	Inverse variance weighted	80	0.02504	0.014088	0.075498	-0.00257	0.052652	1.025356	0.997431	1.054063		
		Weighted median	80	0.040799	0.016158	0.011571	0.009129	0.072469	1.041643	1.009171	1.075159		
		MR Egger	80	0.041575	0.033772	0.221995	-0.02462	0.107768	1.042452	0.975683	1.113789		
		Penalised	80	0.05517	0.01703	0.00119	0.02179	0.08855	1.05672	1.02203	1.09259		

		weighted median		5	1	6	5	6	6	4	5		
		Weighted mode	80	0.058872	0.023663	0.014955	0.012492	0.105252	1.060639	1.01257	1.11099		
		Inverse variance weighted (multiplicative random effects)	80	0.02504	0.014088	0.075498	-0.00257	0.052652	1.025356	0.997431	1.054063		
	Gout	Inverse variance weighted	71	0.034353	0.019448	0.077331	-0.00377	0.072471	1.03495	0.996242	1.075162		
		Weighted median	71	0.031971	0.026992	0.236231	-0.02093	0.084876	1.032488	0.979284	1.088582		
		MR Egger	71	0.048174	0.046787	0.306772	-0.04353	0.139877	1.049353	0.957405	1.150132		
		Penalised weighted median	71	0.032402	0.026412	0.219914	-0.01937	0.08417	1.032932	0.98082	1.087814		
		Weighted mode	71	0.030704	0.035323	0.387693	-0.03853	0.099938	1.03118	0.962203	1.105102		
		Inverse variance weighted (multiplicative random effects)	71	0.034353	0.019448	0.077331	-0.00377	0.072471	1.03495	0.996242	1.075162		
	ReA	Inverse variance weighted	63	0.127498	0.045549	0.005124	0.038221	0.216774	1.135982	1.038961	1.242064	0.361903	5.69E-09
		Weighted median	63	0.094282	0.045502	0.038261	0.005099	0.183465	1.09887	1.005112	1.201373		
		MR Egger	63	0.035617	0.109925	0.747037	-0.17984	0.251071	1.036259	0.835407	1.285401		
		Penalised weighted median	63	0.094209	0.046481	0.042683	0.003105	0.185312	1.098789	1.00311	1.203594		
		Weighted mode	63	0.072093	0.060391	0.237122	-0.04627	0.19046	1.074755	0.95478	1.209806		
		Inverse variance weighted (multiplicative random effects)	63	0.127498	0.045549	0.005124	0.038221	0.216774	1.135982	1.038961	1.242064		
	PA	Inverse variance weighted	80	0.015223	0.030836	0.621543	-0.04522	0.075662	1.015339	0.95579	1.078598		
		Weighted median	80	0.022067	0.048141	0.646686	-0.07229	0.116423	1.022312	0.930261	1.123471		
		MR Egger	80	-0.08852	0.073562	0.232492	-0.2327	0.055663	0.915286	0.792391	1.057241		
		Penalised weighted median	80	0.017447	0.048286	0.717865	-0.07719	0.112088	1.0176	0.92571	1.118611		
		Weighted mode	80	-0.06945	0.079154	0.382897	-0.2246	0.085688	0.932902	0.798838	1.089466		
		Inverse variance weighted (multiplicative random effects)	80	0.015223	0.029598	0.607033	-0.04279	0.073236	1.015339	0.958113	1.075984		

Supplementary Table 14. MR analysis for arthritis to IBD, CD and UC

Exposure	Outcome	Methods	SNP	beta	se	pval	lo_ci	up_ci	or	or_ci	or_hi	pleiotropy (MR-Egger)
RA	IBD	Inverse variance weighted	14	-0.01963	0.063788	0.758301	-0.14465	0.105395	0.980563	0.865324	1.11115	
		Weighted median	14	-0.02849	0.0315	0.365754	-0.09023	0.03325	0.971912	0.913721	1.033809	
		MR Egger	14	-0.01294	0.096602	0.895672	-0.20228	0.176402	0.987145	0.816867	1.192917	
		Penalised weighted median	14	-0.00168	0.027158	0.950605	-0.05491	0.051548	0.998319	0.946568	1.0529	
		Weighted mode	14	-0.03595	0.02797	0.221155	-0.09077	0.018875	0.964692	0.913231	1.019054	
		Inverse variance weighted (multiplicative random effects)	14	-0.01963	0.063788	0.758301	-0.14465	0.105395	0.980563	0.865324	1.11115	
	CD	Inverse variance weighted	14	-0.06791	0.101213	0.502213	-0.26629	0.130462	0.93434	0.766216	1.139355	
		Weighted median	14	-0.01433	0.039205	0.714652	-0.09118	0.062508	0.985768	0.912857	1.064503	
		MR Egger	14	-0.0338	0.152423	0.828245	-0.33255	0.26495	0.966767	0.717095	1.303366	
		Penalised weighted median	14	0.01858	0.032656	0.569387	-0.04543	0.082585	1.018753	0.955591	1.086091	
		Weighted mode	14	0.000752	0.037744	0.984408	-0.07323	0.074731	1.000752	0.92939	1.077594	
		Inverse variance weighted (multiplicative random effects)	14	-0.06791	0.101213	0.502213	-0.26629	0.130462	0.93434	0.766216	1.139355	
	UC	Inverse variance weighted	14	0.043866	0.053932	0.416007	-0.06184	0.149572	1.044843	0.940034	1.161337	
		Weighted median	14	0.040623	0.035996	0.259091	-0.02993	0.111176	1.04146	0.970514	1.117592	
		MR Egger	14	0.058117	0.08156	0.489737	-0.10174	0.217973	1.059839	0.903264	1.243554	
		Penalised weighted median	14	0.036608	0.035603	0.303834	-0.03317	0.10639	1.037287	0.967371	1.112255	
		Weighted mode	14	0.029438	0.034294	0.406228	-0.03778	0.096654	1.029875	0.962926	1.10148	
		Inverse variance weighted (multiplicative random effects)	14	0.043866	0.053932	0.416007	-0.06184	0.149572	1.044843	0.940034	1.161337	
AS	IBD	Inverse variance weighted	5	0.052016	0.110966	0.639242	-0.16548	0.26951	1.053393	0.847489	1.309323	
		Weighted median	5	-0.02686	0.019954	0.178348	-0.06596	0.012255	0.973502	0.936164	1.01233	
		MR Egger	5	-0.14298	0.162203	0.442963	-0.4609	0.174938	0.866772	0.630717	1.191173	
		Penalised weighted median	5	-	0.01822	0.01947	-	-	0.95832	0.92469	0.99316	

				0.04257	2	6	0.07829	0.00686	2	9	7	
		Weighted mode	5	-0.03377	0.018236	0.137682	-0.06952	0.001971	0.966791	0.932845	1.001973	
		Inverse variance weighted (multiplicative random effects)	5	0.052016	0.110966	0.639242	-0.16548	0.26951	1.053393	0.847489	1.309323	
	CD	Inverse variance weighted	5	0.093048	0.115539	0.420624	-0.13341	0.319504	1.097514	0.875108	1.376445	
		Weighted median	5	0.00176	0.025737	0.945471	-0.04869	0.052206	1.001762	0.952481	1.053593	
		MR Egger	5	-0.16094	0.128882	0.300325	-0.41355	0.091666	0.851342	0.661299	1.095999	
		Penalised weighted median	5	-0.02173	0.022274	0.329317	-0.06538	0.021929	0.978506	0.936707	1.022171	
		Weighted mode	5	-0.02277	0.025113	0.415892	-0.07199	0.026454	0.97749	0.930541	1.026807	
		Inverse variance weighted (multiplicative random effects)	5	0.093048	0.115539	0.420624	-0.13341	0.319504	1.097514	0.875108	1.376445	
	UC	Inverse variance weighted	5	0.013269	0.121404	0.912968	-0.22468	0.25122	1.013357	0.79877	1.285593	
		Weighted median	5	-0.07302	0.023375	0.001785	-0.11884	-0.02721	0.929581	0.887952	0.973161	
		MR Egger	5	-0.13496	0.208714	0.563946	-0.54403	0.274123	0.873754	0.580402	1.315376	
		Penalised weighted median	5	-0.07446	0.024569	0.002441	-0.12261	-0.0263	0.928246	0.884605	0.974039	
		Weighted mode	5	-0.07119	0.022481	0.033962	-0.11525	-0.02713	0.931283	0.89114	0.973235	
		Inverse variance weighted (multiplicative random effects)	5	0.013269	0.121404	0.912968	-0.22468	0.25122	1.013357	0.79877	1.285593	
PSA	IBD	Inverse variance weighted	10	-0.13788	0.090547	0.127829	-0.31535	0.039595	0.871205	0.729534	1.040389	
		Weighted median	10	-0.10997	0.041657	0.008292	-0.19162	-0.02832	0.895859	0.825621	0.972073	
		MR Egger	10	-0.23799	0.257341	0.382123	-0.74237	0.266401	0.788213	0.475983	1.305259	
		Penalised weighted median	10	-0.08832	0.041719	0.034266	-0.17009	-0.00655	0.91547	0.843591	0.993474	
		Weighted mode	10	-0.08254	0.059978	0.202054	-0.20009	0.03502	0.920777	0.818654	1.03564	
		Inverse variance weighted (multiplicative random effects)	10	-0.13788	0.090547	0.127829	-0.31535	0.039595	0.871205	0.729534	1.040389	
	CD	Inverse variance weighted	10	-0.11965	0.091054	0.188818	-0.29812	0.058813	0.887229	0.742214	1.060577	
		Weighted median	10	-0.11926	0.051722	0.021119	-0.22064	-0.01789	0.887574	0.802007	0.982271	

		MR Egger	10	-0.26649	0.25485	0.326278	-0.76599	0.233017	0.766065	0.464872	1.262403	
		Penalised weighted median	10	-0.12476	0.052869	0.018285	-0.22838	-0.02114	0.882708	0.795818	0.979085	
		Weighted mode	10	-0.07556	0.086871	0.407034	-0.24582	0.094711	0.927228	0.78206	1.099341	
		Inverse variance weighted (multiplicative random effects)	10	-0.11965	0.091054	0.188818	-0.29812	0.058813	0.887229	0.742214	1.060577	
	UC	Inverse variance weighted	10	-0.14996	0.094117	0.111078	-0.33443	0.034506	0.860741	0.715746	1.035109	
		Weighted median	10	-0.18685	0.048636	0.000122	-0.28218	-0.09153	0.829565	0.754137	0.912536	
		MR Egger	10	-0.19614	0.270337	0.488804	-0.726	0.333722	0.821899	0.483841	1.396156	
		Penalised weighted median	10	-0.17668	0.047042	0.000173	-0.26889	-0.08448	0.838045	0.764231	0.918989	
		Weighted mode	10	-0.19626	0.080164	0.036866	-0.35338	-0.03914	0.821801	0.702311	0.96162	
		Inverse variance weighted (multiplicative random effects)	10	-0.14996	0.094117	0.111078	-0.33443	0.034506	0.860741	0.715746	1.035109	
OA_Knee	IBD	Inverse variance weighted	10	-0.13788	0.090547	0.127829	-0.31535	0.039595	0.871205	0.729534	1.040389	
		Weighted median	10	-0.10997	0.042745	0.010089	-0.19375	-0.02619	0.895859	0.823863	0.974148	
		MR Egger	10	-0.23799	0.257341	0.382123	-0.74237	0.266401	0.788213	0.475983	1.305259	
		Penalised weighted median	10	-0.08832	0.043876	0.044128	-0.17431	-0.00232	0.91547	0.840033	0.997683	
		Weighted mode	10	-0.08254	0.058421	0.191353	-0.19704	0.031969	0.920777	0.821155	1.032485	
		Inverse variance weighted (multiplicative random effects)	10	-0.13788	0.090547	0.127829	-0.31535	0.039595	0.871205	0.729534	1.040389	
	CD	Inverse variance weighted	10	-0.11965	0.091054	0.188818	-0.29812	0.058813	0.887229	0.742214	1.060577	
		Weighted median	10	-0.11926	0.052096	0.022062	-0.22137	-0.01716	0.887574	0.801419	0.982991	
		MR Egger	10	-0.26649	0.25485	0.326278	-0.76599	0.233017	0.766065	0.464872	1.262403	
		Penalised weighted median	10	-0.12476	0.053044	0.018671	-0.22873	-0.02079	0.882708	0.795545	0.97942	
		Weighted mode	10	-0.07556	0.088858	0.417201	-0.24972	0.098605	0.927228	0.779021	1.10363	
		Inverse variance weighted (multiplicative random effects)	10	-0.11965	0.091054	0.188818	-0.29812	0.058813	0.887229	0.742214	1.060577	
	UC	Inverse variance weighted	10	-0.14996	0.094117	0.111078	-0.33443	0.034506	0.860741	0.715746	1.035109	

		Weighted median	10	-0.18685	0.048781	0.000128	-0.28247	-0.09124	0.829565	0.753923	0.912796	
		MR Egger	10	-0.19614	0.270337	0.488804	-0.726	0.333722	0.821899	0.483841	1.396156	
		Penalised weighted median	10	-0.17668	0.045999	0.000123	-0.26684	-0.08652	0.838045	0.765794	0.917113	
		Weighted mode	10	-0.19626	0.079923	0.036421	-0.35291	-0.03961	0.821801	0.702644	0.961165	
		Inverse variance weighted (multiplicative random effects)	10	-0.14996	0.094117	0.111078	-0.33443	0.034506	0.860741	0.715746	1.035109	
OA_Coxa	IBD	Inverse variance weighted	5	0.162275	0.203036	0.42415	-0.23568	0.560226	1.176184	0.790037	1.751068	
		Weighted median	5	0.059474	0.12806	0.642347	-0.19152	0.31047	1.061278	0.8257	1.364067	
		MR Egger	5	-1.35914	0.761886	0.172438	-2.85244	0.134156	0.256881	0.057703	1.143571	
		Penalised weighted median	5	0.007113	0.131965	0.957012	-0.25154	0.265765	1.007139	0.777604	1.304428	
		Weighted mode	5	-0.02918	0.168681	0.871057	-0.3598	0.301435	0.971241	0.697819	1.351797	
		Inverse variance weighted (multiplicative random effects)	5	0.162275	0.203036	0.42415	-0.23568	0.560226	1.176184	0.790037	1.751068	
	CD	Inverse variance weighted	5	0.009415	0.187314	0.959913	-0.35772	0.376549	1.009459	0.699269	1.457248	
		Weighted median	5	-0.02433	0.160476	0.879482	-0.33886	0.2902	0.975961	0.712579	1.336695	
		MR Egger	5	-0.0672	1.084904	0.954509	-2.19361	2.059216	0.935013	0.111514	7.839823	
		Penalised weighted median	5	-0.08006	0.160746	0.618442	-0.39512	0.235001	0.92306	0.673597	1.26491	
		Weighted mode	5	-0.06546	0.205373	0.765877	-0.46799	0.337071	0.936637	0.62626	1.400839	
		Inverse variance weighted (multiplicative random effects)	5	0.009415	0.187314	0.959913	-0.35772	0.376549	1.009459	0.699269	1.457248	
	UC	Inverse variance weighted	5	0.28589	0.472251	0.544929	-0.63972	1.211501	1.330946	0.527439	3.358522	
		Weighted median	5	-0.1594	0.148037	0.281577	-0.44956	0.130748	0.852652	0.637912	1.139681	
		MR Egger	5	-2.77692	2.054336	0.269346	-6.80342	1.249577	0.06223	0.00111	3.488865	
		Penalised weighted median	5	-0.18652	0.155416	0.230078	-0.49114	0.118092	0.829838	0.611928	1.125347	
		Weighted mode	5	-0.20697	0.149003	0.237165	-0.49901	0.085081	0.813048	0.607131	1.088805	
		Inverse variance weighted (multiplicative random effects)	5	0.28589	0.472251	0.544929	-0.63972	1.211501	1.330946	0.527439	3.358522	
Gout	IBD	Inverse variance weighted	4	0.06844	0.12801	0.59288	-	0.31936	1.07084	0.83321	1.37624	

				6	8	4	0.18247		3	1	7	
		Weighted median	4	0.01346	0.031059	0.664742	-0.04742	0.074336	1.013551	0.953691	1.077168	
		MR Egger	4	-0.00284	0.228748	0.991214	-0.45119	0.445504	0.997162	0.63687	1.561277	
		Penalised weighted median	4	0.004458	0.030179	0.882567	-0.05469	0.063609	1.004468	0.946776	1.065675	
		Weighted mode	4	0.006101	0.031423	0.858458	-0.05549	0.06769	1.00612	0.946023	1.070034	
		Inverse variance weighted (multiplicative random effects)	4	0.068446	0.128018	0.592884	-0.18247	0.31936	1.070843	0.833211	1.376247	
	CD	Inverse variance weighted	4	0.114178	0.177537	0.520146	-0.2338	0.462151	1.120951	0.791524	1.587485	
		Weighted median	4	-0.00297	0.038478	0.938534	-0.07838	0.072449	0.997037	0.92461	1.075138	
		MR Egger	4	-0.06908	0.282589	0.829664	-0.62296	0.484791	0.933249	0.536356	1.623836	
		Penalised weighted median	4	-0.00766	0.037745	0.839226	-0.08164	0.066323	0.992371	0.921605	1.068572	
		Weighted mode	4	-0.00847	0.036023	0.829272	-0.07907	0.062137	0.991567	0.923972	1.064108	
		Inverse variance weighted (multiplicative random effects)	4	0.114178	0.177537	0.520146	-0.2338	0.462151	1.120951	0.791524	1.587485	
	UC	Inverse variance weighted	4	0.031923	0.084428	0.705351	-0.13356	0.197401	1.032438	0.874979	1.218233	
		Weighted median	4	0.039561	0.039116	0.311842	-0.03711	0.116228	1.040354	0.963573	1.123252	
		MR Egger	4	0.05533	0.155299	0.755707	-0.24906	0.359716	1.056889	0.779536	1.432922	
		Penalised weighted median	4	0.024514	0.038224	0.521302	-0.0504	0.099432	1.024817	0.950845	1.104544	
		Weighted mode	4	0.035582	0.041598	0.455213	-0.04595	0.117113	1.036223	0.955091	1.124247	
		Inverse variance weighted (multiplicative random effects)	4	0.031923	0.084428	0.705351	-0.13356	0.197401	1.032438	0.874979	1.218233	
ReA	IBD	Inverse variance weighted	3	-0.09302	0.04232	0.027955	-0.17596	-0.01007	0.911179	0.838649	0.989982	0.227165
		Weighted median	3	-0.10548	0.024233	1.34E-05	-0.15298	-0.05798	0.899891	0.858148	0.943664	
		MR Egger	3	-0.20986	0.048925	0.145811	-0.30576	-0.11397	0.810696	0.736566	0.892285	
		Penalised weighted median	3	-0.10805	0.024396	9.47E-06	-0.15587	-0.06023	0.897581	0.855671	0.941543	
		Weighted mode	3	-0.10847	0.027007	0.056768	-0.1614	-0.05553	0.897207	0.850949	0.94598	
		Inverse variance weighted (multiplicative random effects)	3	-0.09302	0.04232	0.027955	-0.17596	-0.01007	0.911179	0.838649	0.989982	
	CD	Inverse variance weighted	3	0.00582	0.04274	0.89155	-	0.08959	1.00584	0.92501	1.09373	

				7			0.07794	9	4	6	5	
		Weighted median	3	-0.01999	0.035195	0.569959	-0.08898	0.048988	0.980204	0.914866	1.050207	
		MR Egger	3	0.056398	0.119853	0.720001	-0.17851	0.29131	1.058019	0.836513	1.338179	
		Penalised weighted median	3	-0.04145	0.033799	0.220114	-0.10769	0.024801	0.959402	0.897904	1.025111	
		Weighted mode	3	0.065642	0.043791	0.272627	-0.02019	0.151473	1.067844	0.980014	1.163546	
		Inverse variance weighted (multiplicative random effects)	3	0.005827	0.04274	0.89155	-0.07794	0.089599	1.005844	0.925016	1.093735	
	UC	Inverse variance weighted	3	-0.19108	0.127098	0.132729	-0.4402	0.05803	0.826064	0.64391	1.059746	
		Weighted median	3	-0.19759	0.035069	1.76E-08	-0.26633	-0.12886	0.820703	0.766187	0.879098	
		MR Egger	3	-0.51788	0.136197	0.163717	-0.78483	-0.25093	0.595782	0.456199	0.778074	
		Penalised weighted median	3	-0.14349	0.040619	0.000411	-0.22311	-0.06388	0.866325	0.800028	0.938117	
		Weighted mode	3	-0.2045	0.037021	0.031243	-0.27706	-0.13194	0.815053	0.758008	0.876392	
		Inverse variance weighted (multiplicative random effects)	3	-0.19108	0.127098	0.132729	-0.4402	0.05803	0.826064	0.64391	1.059746	
PA	IBD	NA	NA	NA	NA	NA	NA	NA	NA	NA	NA	
	CD	NA	NA	NA	NA	NA	NA	NA	NA	NA	NA	
	UC	NA	NA	NA	NA	NA	NA	NA	NA	NA	NA	

Supplementary Table 15. MR analysis for IBD, CD and UC to metabolites of gut microbiota

Exposure	Mediator	nSNP	OR	OR_low	OR_hi	p	pleiotropy (MR-Egger)	heterogeneity
IBD	Acetate levels	130	0.992	0.981	1.003	0.155		
	Butyrate levels	127	1.027	1.003	1.051	0.025	0.5170	0.6467
	Serotonin	87	0.988	0.977	0.998	0.023	0.2205	0.0003
	Tryptophan levels	126	1.003	0.991	1.016	0.623		
	Uridine levels	125	0.982	0.964	1.002	0.070		
	Taurine levels	126	0.989	0.975	1.004	0.144		
	lipopolysaccharide activity	129	0.998	0.973	1.024	0.896		
	Hyodeoxycholate	87	1.012	0.994	1.030	0.181		
	Kynurenine levels	125	1.005	0.983	1.026	0.680		
CD	Acetate levels	114	0.992	0.982	1.002	0.133		
	Butyrate levels	111	1.016	0.995	1.037	0.139		
	Serotonin	80	0.998	0.990	1.006	0.609		
	Tryptophan levels	112	1.003	0.992	1.014	0.621		
	Uridine levels	112	0.984	0.967	1.001	0.061		
	Taurine levels	111	0.998	0.985	1.011	0.731		
	lipopolysaccharide activity	113	1.005	0.984	1.028	0.627		
	Hyodeoxycholate	87	1.012	0.994	1.030	0.181		
	Kynurenine levels	111	1.005	0.983	1.026	0.740		
UC	Acetate levels	84	1.005	0.996	1.014	0.244		
	Butyrate levels	80	1.011	0.986	1.037	0.377		
	Serotonin	54	0.990	0.978	1.002	0.097		
	Tryptophan levels	79	1.002	0.989	1.016	0.769		
	Uridine levels	79	0.991	0.971	1.012	0.401		
	Taurine levels	80	1.003	0.988	1.019	0.707		
	lipopolysaccharide activity	83	0.991	0.964	1.018	0.494		
	Hyodeoxycholate	54	1.016	0.996	1.036	0.110		
	Kynurenine levels	80	1.006	0.984	1.030	0.586		

Supplementary Table 16. MR analysis for IBD, CD and UC to serum biochemical indicators

Exposure	Mediator	nSNP	OR	OR_low	OR_hi	p	pleiotropy (MR-Egger)	heterogeneity
IBD	Total cholesterol levels	128	1.000	0.988	1.013	0.956		
	Low density lipoprotein cholesterol levels	130	0.999	0.985	1.013	0.840		
	Triglyceride levels	120	1.004	0.995	1.013	0.350		
	Serum uric acid levels	127	1.003	0.991	1.015	0.668		
	Serum albumin levels	129	0.988	0.975	1.000	0.056		
	Polyunsaturated fatty acids	130	1.006	0.989	1.023	0.489		
	Omega-3 fatty acids	130	1.006	0.990	1.022	0.486		
CD	Total cholesterol levels	111	0.999	0.988	1.010	0.866		
	Low density lipoprotein cholesterol levels	114	0.999	0.987	1.012	0.921		
	Triglyceride levels	107	1.005	0.998	1.012	0.198		
	Serum uric acid levels	111	1.003	0.992	1.015	0.610		
	Serum albumin levels	112	0.991	0.979	1.003	0.146		
	Polyunsaturated fatty acids	114	1.008	0.993	1.022	0.314		
	Omega-3 fatty acids	114	1.008	0.994	1.022	0.270		
UC	Total cholesterol levels	84	1.003	0.995	1.012	0.480		
	Low density lipoprotein cholesterol levels	84	1.000	0.990	1.009	0.935		
	Triglyceride levels	82	0.993	0.984	1.001	0.094		
	Serum uric acid levels	83	1.002	0.994	1.010	0.602		
	Serum albumin levels	84	0.986	0.977	0.995	0.002	0.6034	1.65E-37
	Polyunsaturated fatty acids	84	0.996	0.984	1.009	0.576		
	Omega-3 fatty acids	84	0.997	0.984	1.010	0.609		

Supplementary Table 17. MR analysis for IBD, CD and UC to inflammatory factors and immune molecules

Exposure	Mediator	nSNP	OR	OR_low	OR_hi	p	pleiotropy (MR-Egger)	heterogeneity
IBD	PCT	130	1.018	0.972	1.067	0.443		
	CRP	130	1.028	1.011	1.045	0.001	0.4158	< 0.001
	Neutrophil percentage of white cells	130	1.025	1.006	1.043	0.009	0.8675	< 0.001
	Lymphocyte percentage of white cells	130	0.974	0.957	0.991	0.003	0.7235	< 0.001
	Interleukin-6 levels	130	1.036	1.011	1.061	0.005	0.4895	0.0249
	Interleukin-12	130	0.960	0.918	1.004	0.071		
	TNF-α	119	1.004	0.959	1.051	0.856		
	IFN-γ	120	1.010	0.979	1.042	0.538		
CD	PCT	114	0.999	0.961	1.040	0.973		
	CRP	108	1.021	1.006	1.037	0.007	0.5104	< 0.001
	Neutrophil percentage of white cells	114	1.024	1.006	1.042	0.009	0.8542	< 0.001
	Lymphocyte percentage of white cells	114	0.979	0.962	0.996	0.013	0.9220	< 0.001
	Interleukin-6 levels	114	1.026	1.005	1.048	0.015	0.4228	0.0711
	Interleukin-12	114	0.957	0.920	0.997	0.034	0.0076	< 0.001
	TNF-α	106	1.005	0.965	1.047	0.807		
	IFN-γ	106	1.000	0.973	1.027	0.975		
UC	PCT	84	1.016	0.967	1.068	0.528		
	CRP	82	1.014	1.002	1.026	0.018	0.9262	< 0.001
	Neutrophil percentage of white cells	84	1.007	0.989	1.026	0.451		
	Lymphocyte percentage of white cells	84	0.987	0.968	1.005	0.161		
	Interleukin-6 levels	84	1.031	1.004	1.058	0.022	0.3790	0.0588
	Interleukin-12	84	0.962	0.917	1.009	0.111		
	TNF-α	78	0.976	0.927	1.027	0.354		
	IFN-γ	78	1.018	0.983	1.054	0.313		

Supplementary Table 18. MR analysis for IBD, CD and UC to nutrition and metabolism

Exposure	Mediator	nSNP	OR	OR_low	OR_hi	p	pleiotropy (MR-Egger)	heterogeneity
IBD	Vitamin B12	128	0.996	0.986	1.006	0.439		
	Vitamin B6	128	0.992	0.982	1.002	0.106		
	Vitamin C	128	0.997	0.986	1.008	0.615		
	25-Hydroxyvitamin D	130	0.997	0.991	1.003	0.287		
	Calcium levels	130	0.999	0.987	1.012	0.911		
	Total body bone mineral density	129	0.982	0.968	0.996	0.014	0.1664	< 0.001
	Urolithiasis	129	1.053	1.009	1.098	0.017		
	Osteocalcin	130	1.013	0.964	1.065	0.616		
CD	Vitamin B12	113	0.995	0.986	1.003	0.226		
	Vitamin B6	113	0.996	0.987	1.004	0.322		
	Vitamin C	113	1.003	0.994	1.012	0.545		
	25-Hydroxyvitamin D	115	0.996	0.990	1.002	0.160		
	Calcium levels	114	1.001	0.991	1.012	0.829		
	Total body bone mineral density	112	0.991	0.977	1.005	0.210		
	Urolithiasis	113	1.047	1.014	1.081	0.005	0.8533	< 0.001
	Osteocalcin	114	1.025	0.981	1.070	0.267		
UC	Vitamin B12	82	1.001	0.989	1.012	0.930		
	Vitamin B6	82	0.992	0.980	1.004	0.177		
	Vitamin C	82	0.999	0.985	1.012	0.832		
	25-Hydroxyvitamin D	84	1.016	0.967	1.068	0.356		
	Calcium levels	84	0.996	0.987	1.004	0.345		
	Total body bone mineral density	84	0.974	0.959	0.990	0.002	0.8949	< 0.001
	Urolithiasis	84	1.024	0.981	1.070	0.279		
	Osteocalcin	84	0.975	0.923	1.030	0.374		

Supplementary Table 19. MR analysis for mediators to arthritis

Outcome	Mediator	nSNP	OR	OR_low	OR_hi	p
AS	Butyrate levels	36	0.9287	0.8255	1.0449	0.219
	Serotonin	11	1.6780	0.8343	3.3748	0.147
	Serum albumin levels	145	1.1786	0.9127	1.5220	0.208
	CRP	157	1.5548	0.9966	2.4258	0.052
	Neutrophil percentage of white cells	222	1.5193	0.9782	2.3599	0.063
	Lymphocyte percentage of white cells	234	0.8433	0.6716	1.0591	0.143
	Interleukin-6 levels	12	1.4490	0.4843	4.3354	0.507
	Total body bone mineral density	79	1.1259	0.9946	1.2745	0.061
	Urolithiasis	30	1.1216	0.9889	1.2722	0.074
PSA	Butyrate levels	36	0.9387	0.8456	1.0419	0.235
	Serotonin	11	1.5143	0.7831	2.9283	0.218
	Serum albumin levels	145	1.2886	1.0250	1.6199	0.030
	CRP	157	1.1493	0.9766	1.3527	0.094
	Neutrophil percentage of white cells	222	1.3632	1.1113	1.6723	0.003
	Lymphocyte percentage of white cells	235	0.8221	0.6962	0.9707	0.021
	Interleukin-6 levels	12	0.8696	0.7081	1.0679	0.183
	Total body bone mineral density	79	1.1306	0.9965	1.2828	0.057
	Urolithiasis	30	1.1187	0.9834	1.2726	0.088
ReA	Butyrate levels	36	0.9862	0.8862	1.0976	0.800
	Serotonin	11	0.7696	0.2389	2.4793	0.661
	Serum albumin levels	145	1.1473	0.9278	1.4187	0.205
	CRP	157	1.2912	0.9969	1.6723	0.053
	Neutrophil percentage of white cells	222	1.0471	0.8135	1.3478	0.721
	Lymphocyte percentage of white cells	235	1.0348	0.8633	1.2403	0.711
	Interleukin-6 levels	12	1.3805	0.8714	2.1868	0.170
	Total body bone mineral density	79	1.1799	1.0410	1.3375	0.010
	Urolithiasis	30	1.0049	0.9005	1.1213	0.931

Supplementary Figure 1. Figures for IBD to AS.

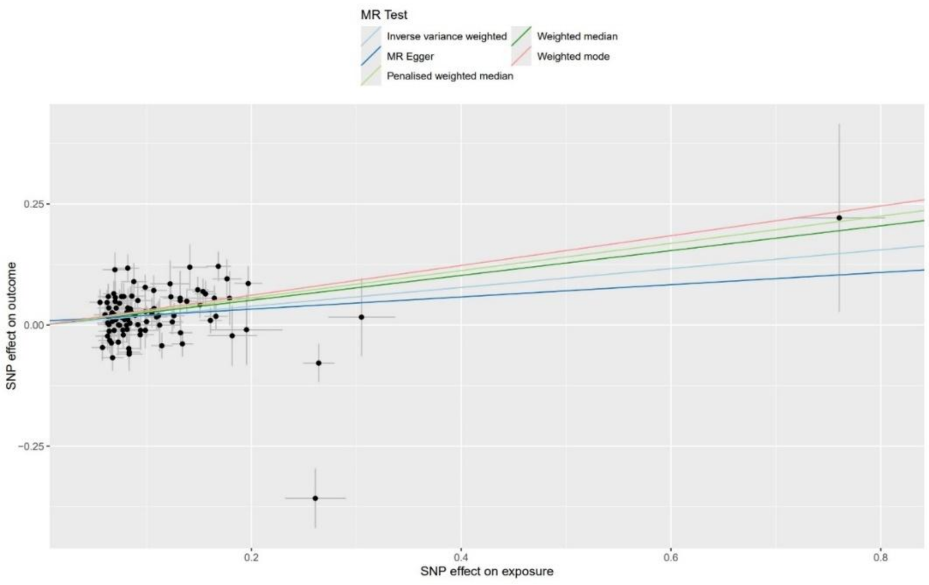

Scatter plot

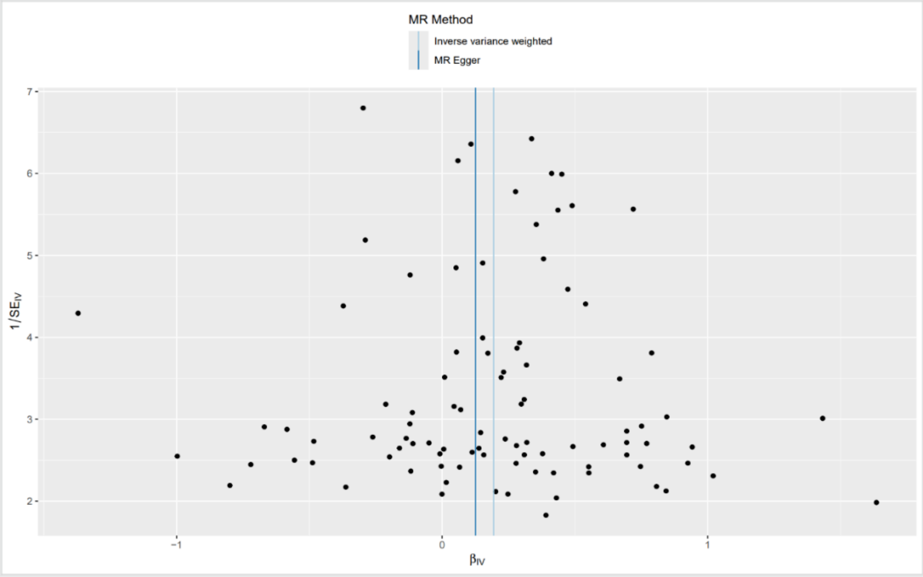

Funnel plot

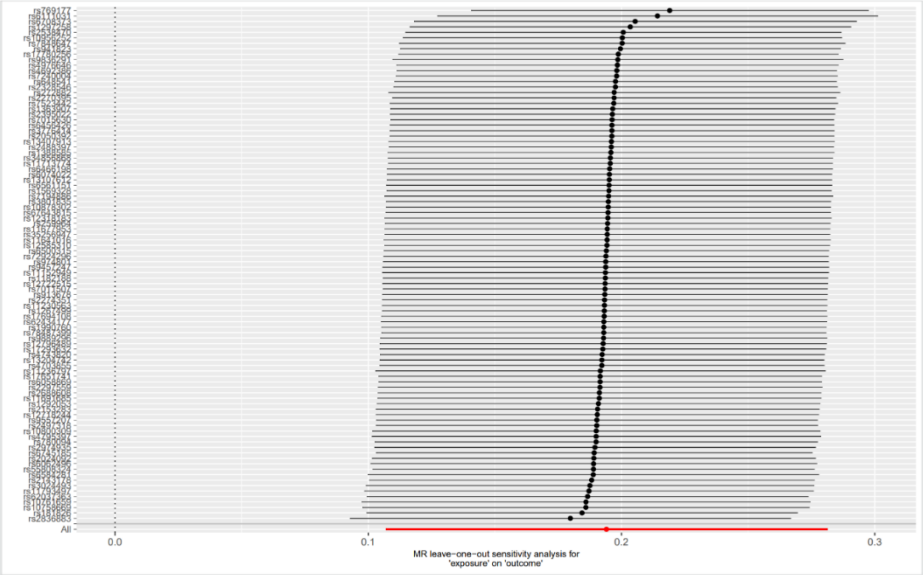

Leave-one-out plot

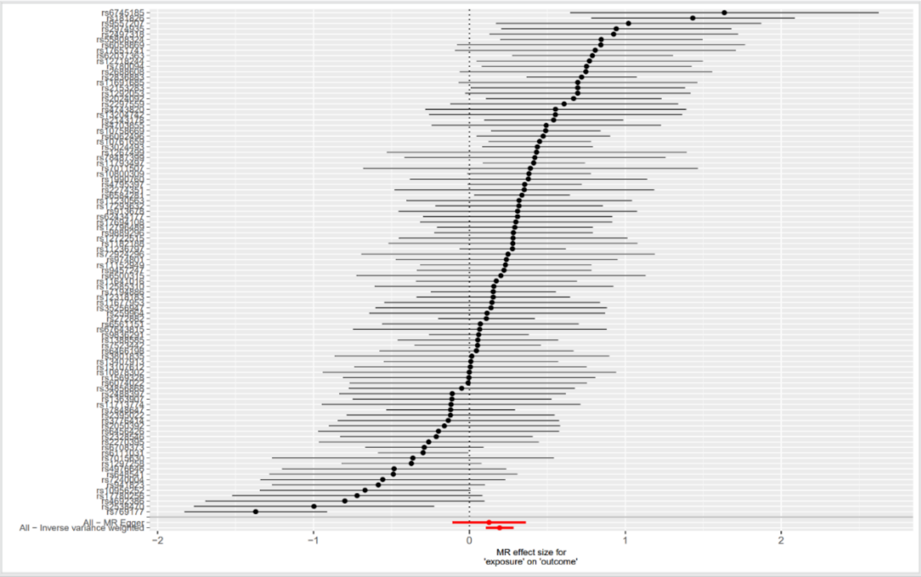

Forest plot

Supplementary Figure 2. Figures for IBD to PSA.

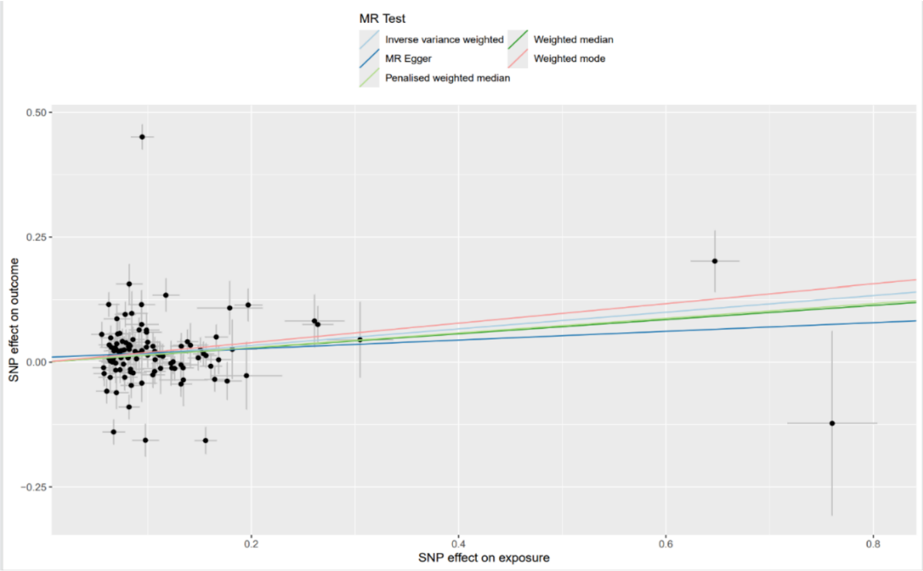

Scatter plot

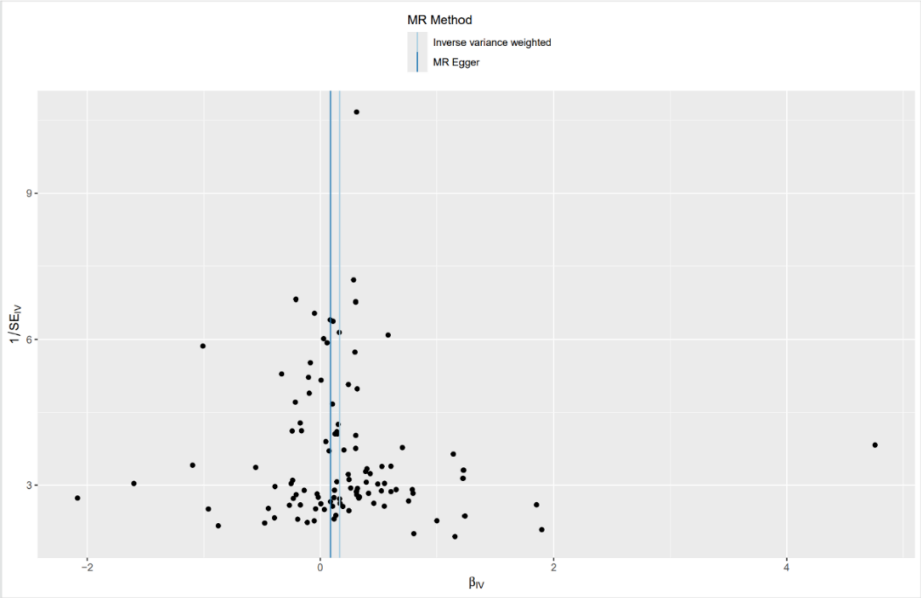

Funnel plot

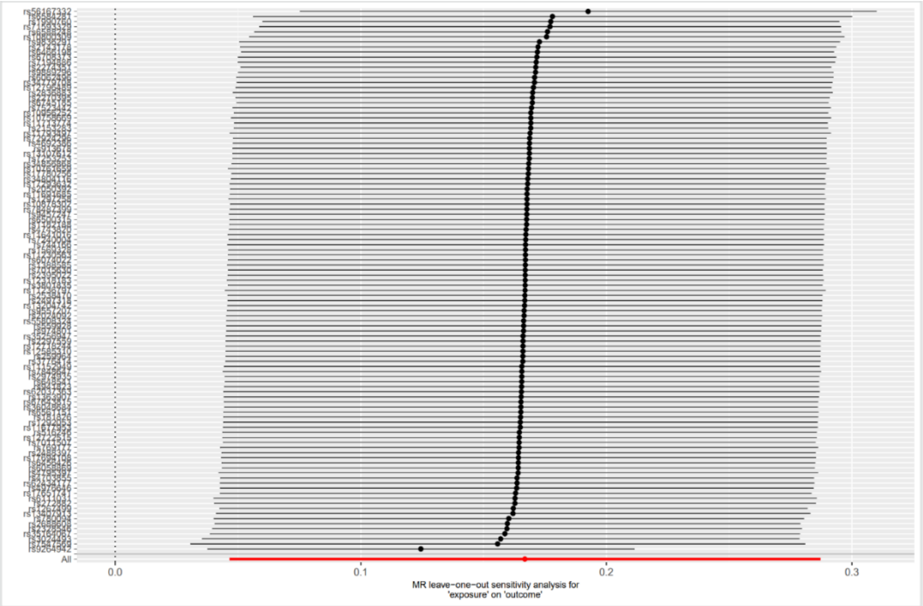

Leave-one-out plot

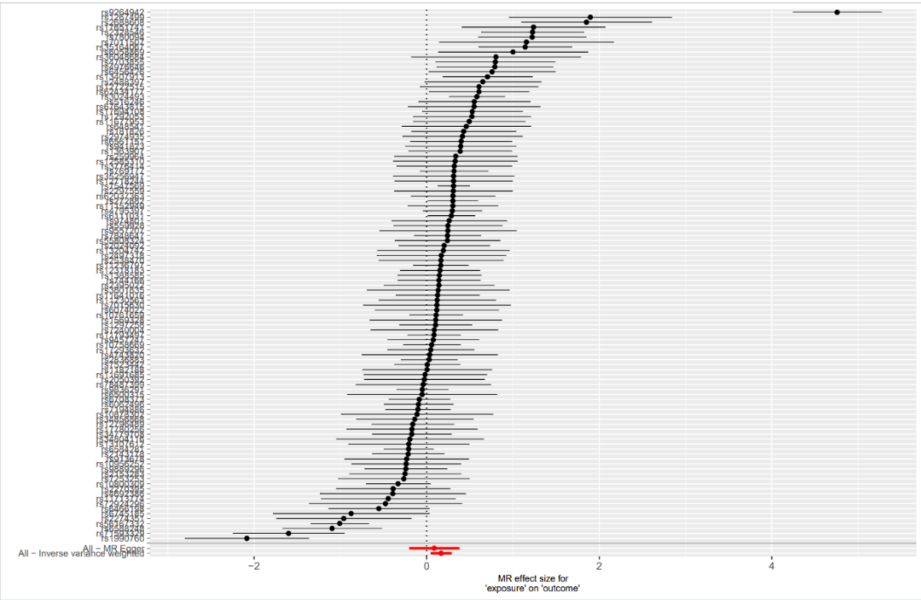

Forest plot

Supplementary Figure 3. Figures for IBD to ReA.

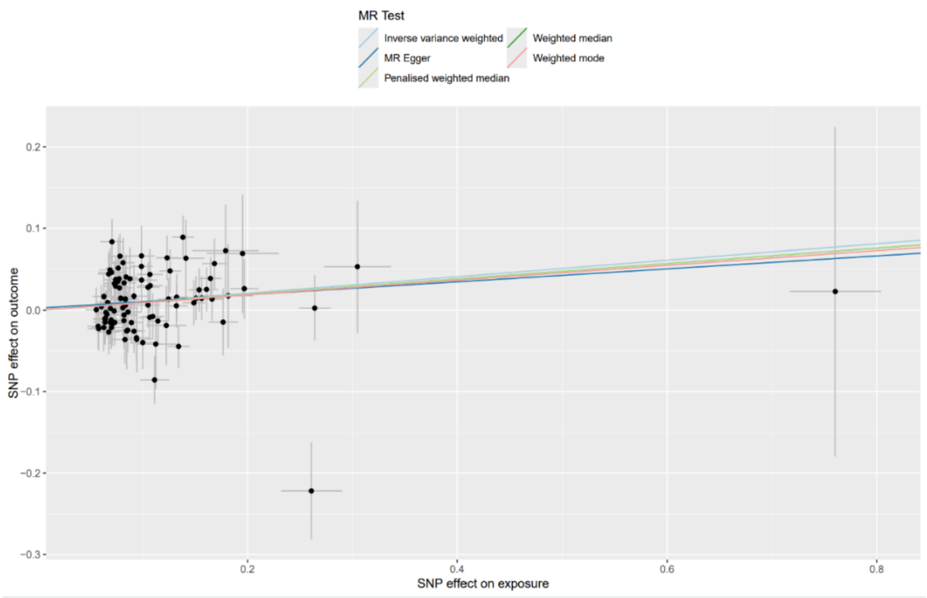

Scatter plot

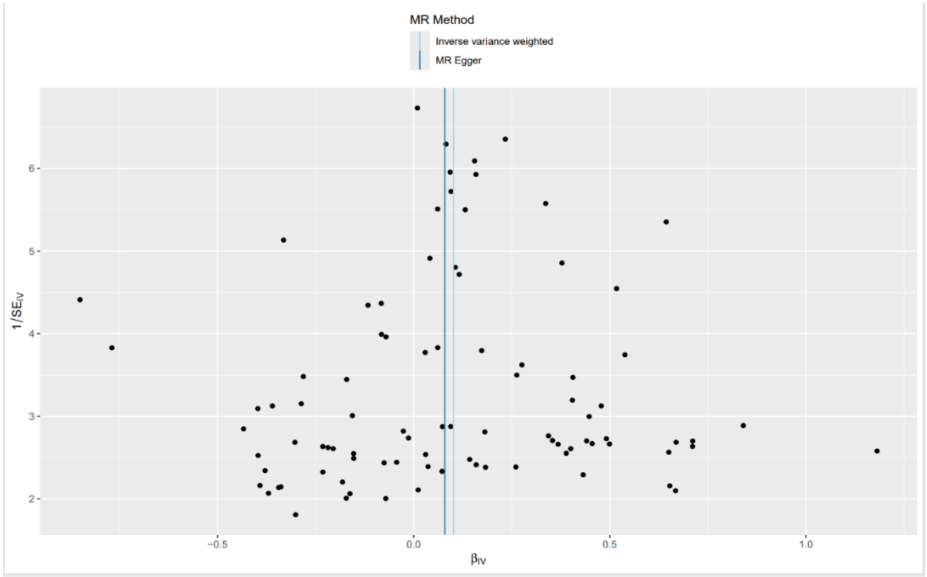

Funnel plot

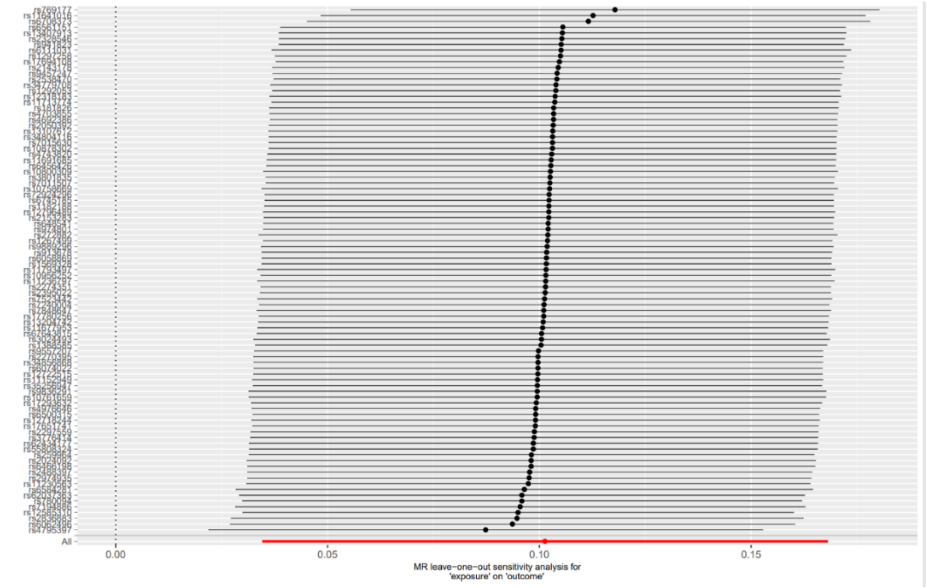

Leave-one-out plot

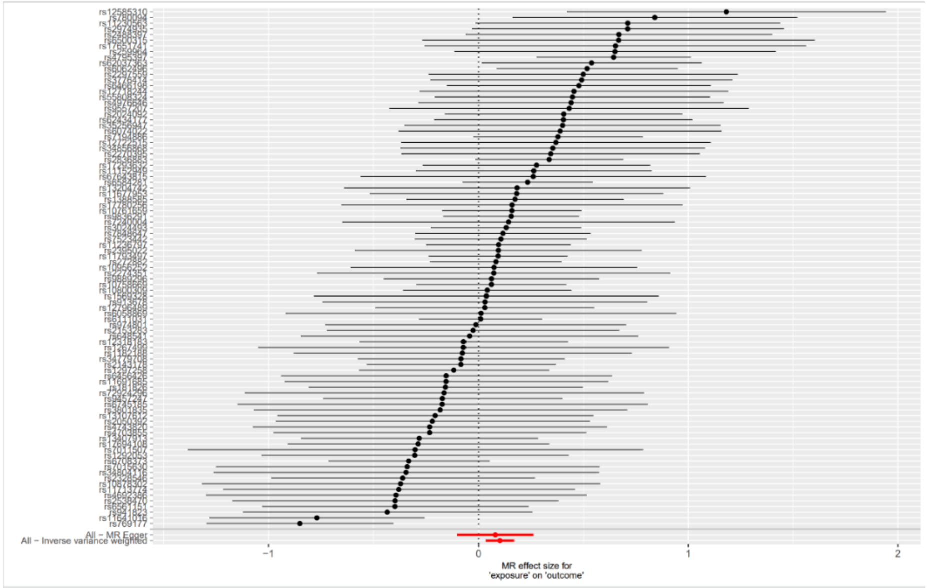

Forest plot

Supplementary Figure 4. Figures for CD to AS.

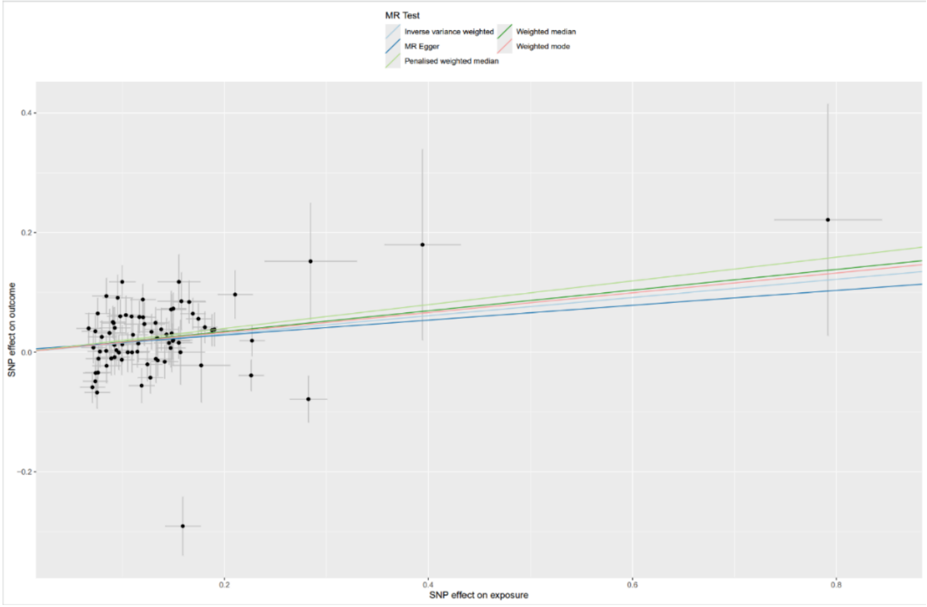

Scatter plot

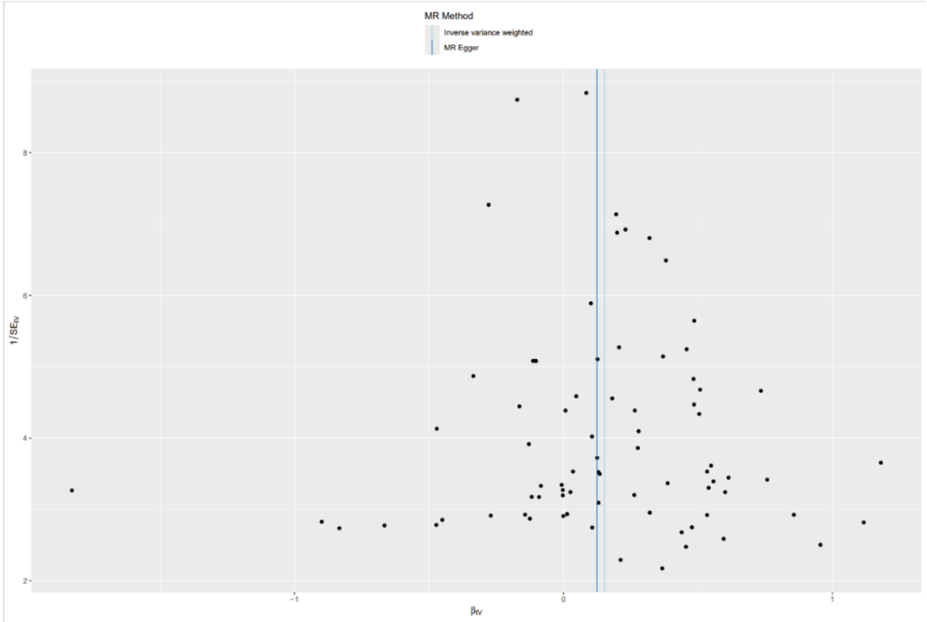

Funnel plot

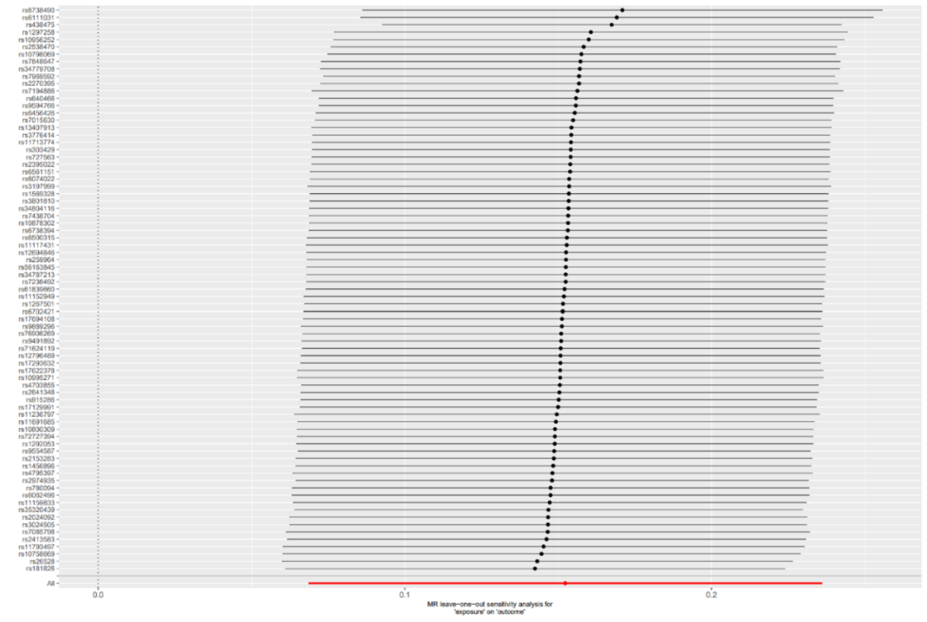

Leave-one-out plot

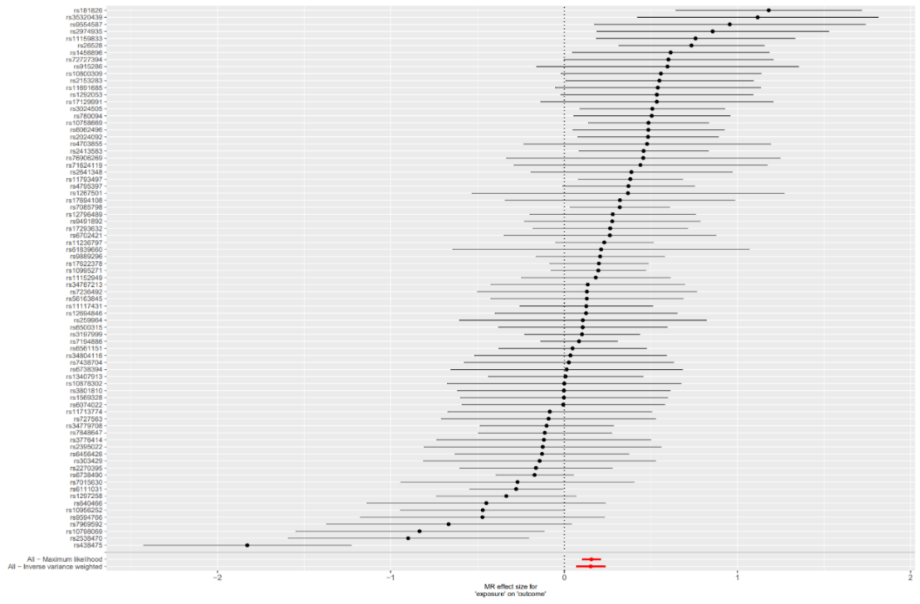

Forest plot

Supplementary Figure 5. Figures for UC to ReA.

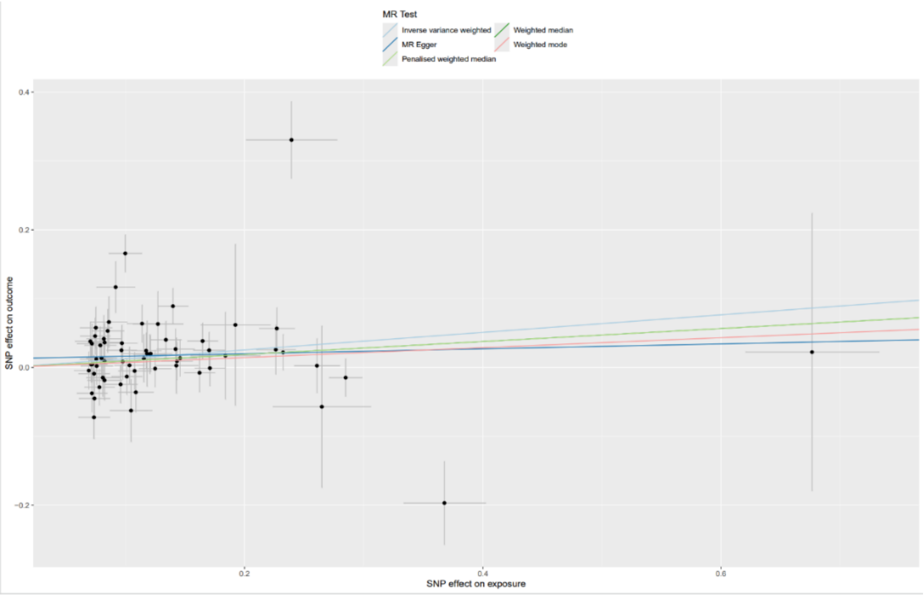

Scatter plot

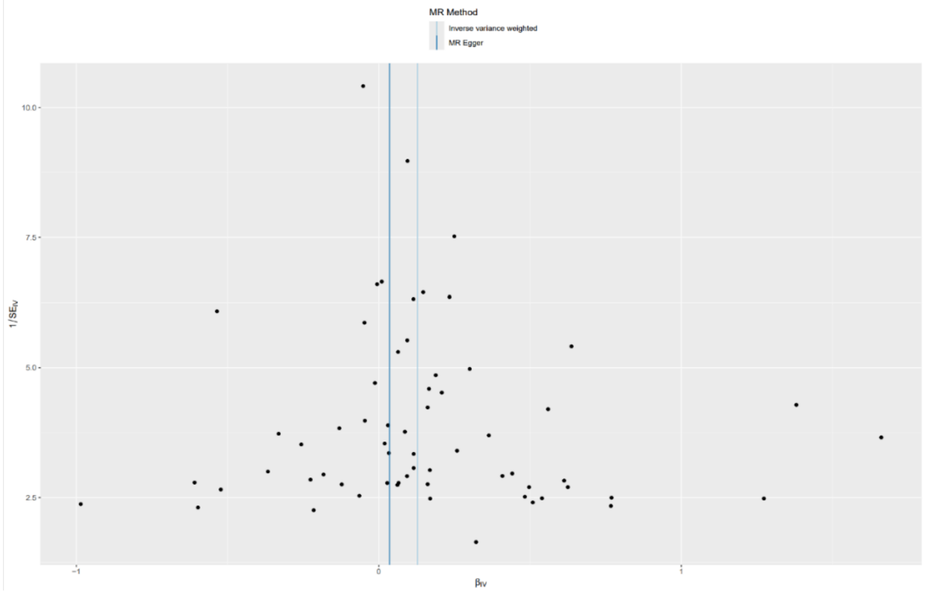

Funnel plot

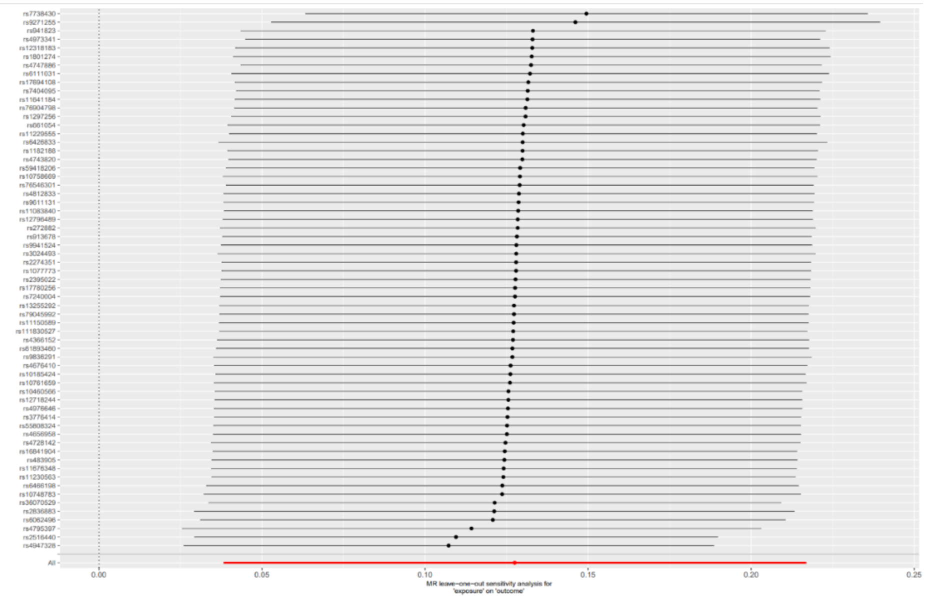

Leave-one-out plot

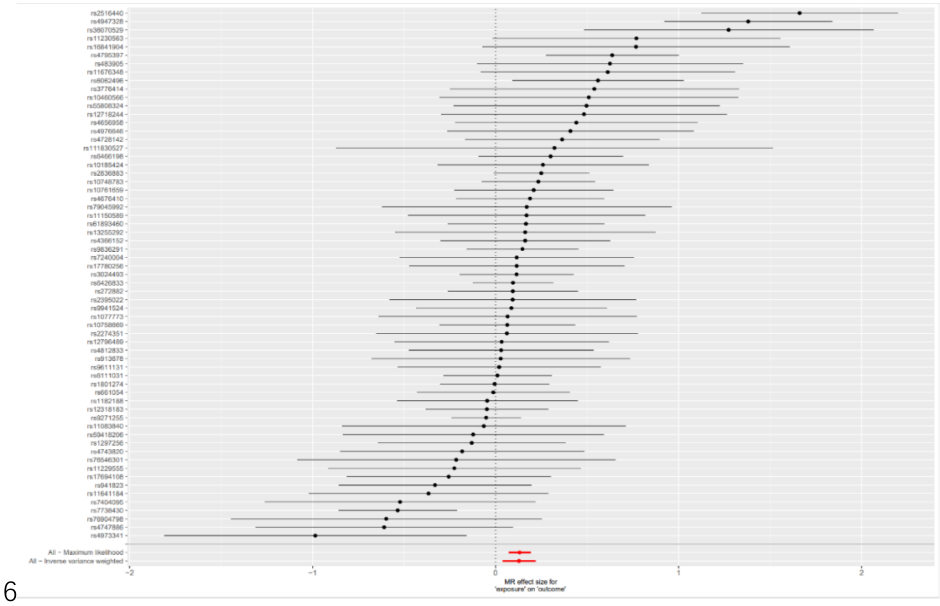

Forest plot
